# Spatially Resolved Greenhouse Gas Emissions of U.S. Milk Production in 2020

**DOI:** 10.1021/acs.est.5c01166

**Published:** 2025-05-07

**Authors:** Rylie Pelton, Juan Tricarico, Fabian Bernal, Mary Beth de Ondarza, Tim Kurt

**Affiliations:** † LEIF LLC, St. Paul, Minnesota 55113, United States; ‡ University of Minnesota, Institute on the Environment, St. Paul, Minnesota 55108, United States; § 6972 Dairy Management Inc., Rosemount, Illinois 60018, United States; ∥ Paradox Nutrition, LLC., Plattsburgh, New York 12901, United States

**Keywords:** milk production, LCA, greenhouse gas emissions

## Abstract

This study provides a comprehensive and spatially resolved assessment of greenhouse gas (GHG) emissions from the U.S. dairy industry, spanning from cradle to farm gate. The primary goal is to refine emission estimates for key sources, including enteric fermentation, manure, and feed production, using the latest climate science, predictive models, and updated industry data, including a broader range of dietary rations across 12 distinct dairy regions. Compared with previous studies that employed generalized models and less granular data, this approach offers greater accuracy and regional specificity. In addition to establishing the 2020 GHG emissions baseline, we compare the results to 2007 estimates to highlight trends and improvements in emission intensities and production practices. U.S. raw milk production in 2020 generated 138.88 million tonnes of CO_2_e, corresponding to an average of 1.38 kg CO_2_e per kg of fat- and protein-corrected milk (FPCM), with regional emissions ranging from 1.24 to 1.87 kg CO_2_e/kg FPCM. Notably, the study shows that while enteric fermentation and manure emissions remain substantial contributors, their share of total emissions is less than in previous assessments. A sensitivity analysis explores the impact of key methodological choices, ensuring robust results that support mitigation strategies and inform the dairy sector’s path toward net neutrality by 2050.

## Graphical abstract

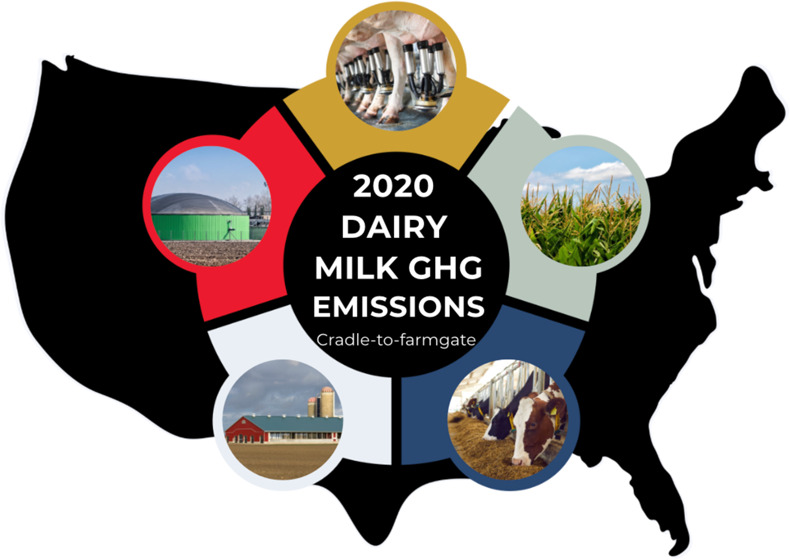

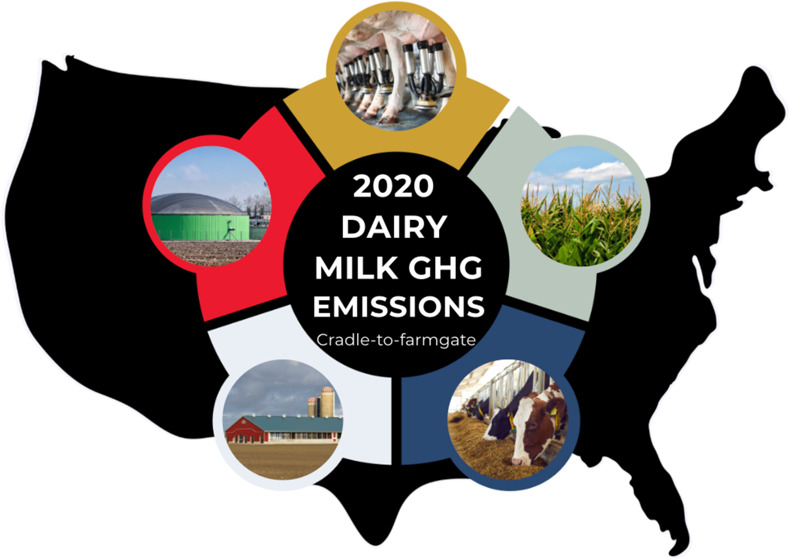

## 1 Introduction

As the second-largest milk producer in the world, the United States (U.S.) dairy industry has long been a vital component of the nation’s agricultural sector, providing essential nutrients and products to millions. However, the environmental impact of dairy production, particularly greenhouse gas (GHG) emissions, has come under increasing scrutiny. The U.S. dairy industry has committed to collectively achieving GHG neutrality by 2050. This ambitious goal necessitates a comprehensive understanding of the current emissions landscape, forming the foundation upon which effective mitigation strategies can be built.

Life cycle assessment (LCA) is a method used to track the inputs, outputs, and potential environmental impacts associated with product systems, from the extraction of resources through manufacturing, distribution, use, and disposal, depending on where the system scope and boundary are set. While several LCAs have been developed for U.S. dairy milk production,^1–6^ significant advancements in LCA methodologies, such as regionalization of life cycle inventories, changes to global warming potential (GWP) characterization factors, and a deeper understanding of emission sources have emerged, particularly in areas such as enteric fermentation, manure management, and crop production. Further, the U.S. dairy industry has seen meaningful changes in milk yields, farm sizes and herd locations, dry matter intake, body weights, and other performance parameters. These advancements highlight the need for an updated assessment to accurately reflect the current state of the industry and provide a new baseline for future GHG emission reduction efforts.

The goal of this study is to (1) estimate ISO-conforming baseline cradle-to-farm-gate GHG emissions (Figure 1) of raw milk production across the contiguous U.S. and 12 distinct dairy regions for 2020, both in terms of absolute emissions as measured in carbon dioxide equivalents (CO_2_e) and emission intensity (kg CO_2_e per kg of fat- and protein-corrected milk, FPCM); (2) identify updated emission hotspots across key sources and GHG types, highlighting priorities for achieving net-neutral emissions; (3) compare results to 2007 estimates to assess changes in emission intensity over time, and (4) investigate the sensitivity of the results to parameter uncertainty, use of alternative climate metrics, and changes in methods used for estimating methane (CH_4_) and nitrous oxide (N_2_O) emissions, reflecting those used in previous LCA studies. By leveraging the latest scientific advancements and data on diets, manure management, crop production practices, herd locations, and other growth and performance parameters, this study will offer a more accurate and comprehensive view of the industry’s GHG footprint across the U.S., capturing regional variations and enabling identification of targeted opportunities for mitigation. The results support the U.S. dairy industry’s goal of achieving GHG neutrality by 2050 by providing a robust baseline for measuring future reductions.

**Figure 1:**
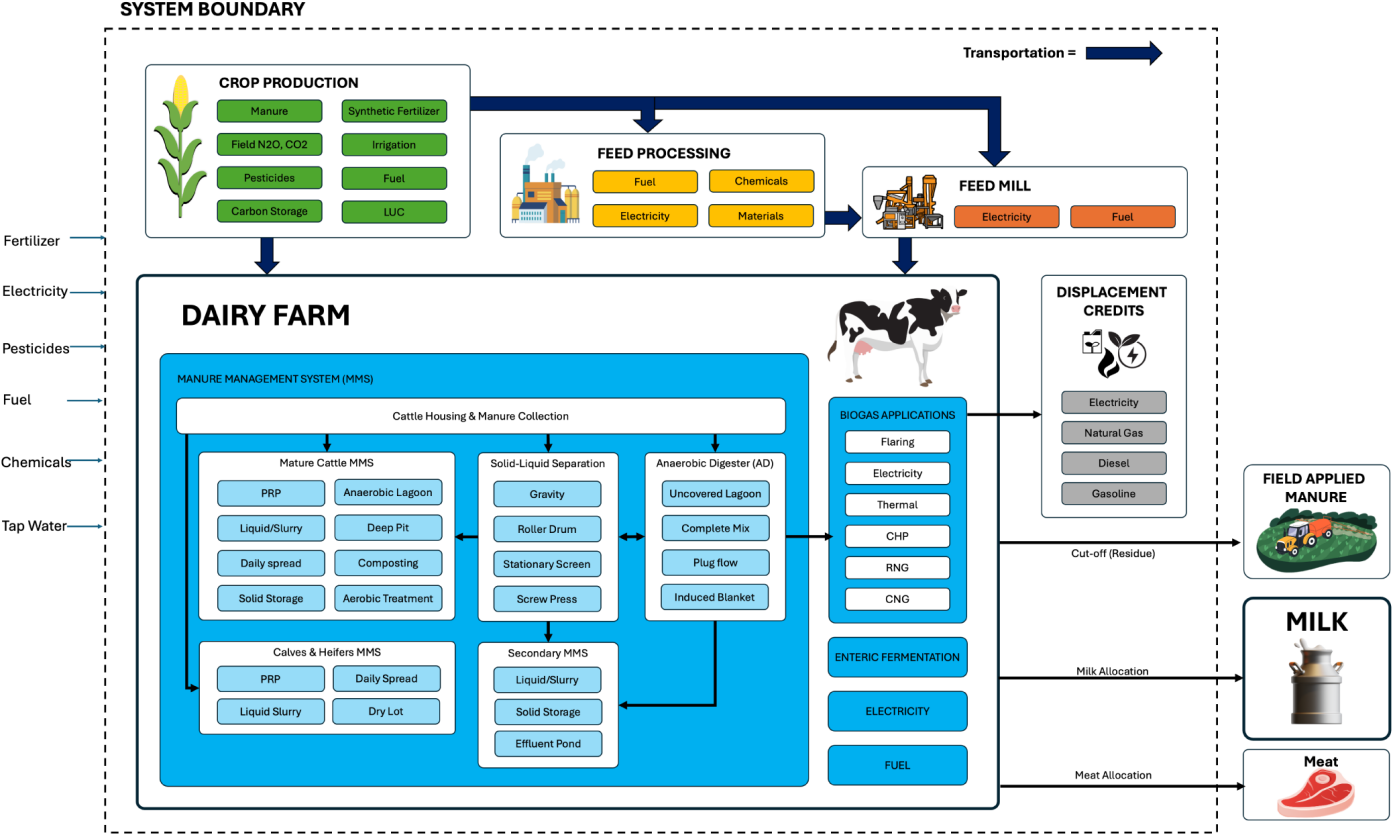
System boundary for cradle-to-farm-gate LCA.

## 2 Materials and Methods

This cradle-to-farm-gate analysis considers emissions across 12 U.S. dairy regions (Figure 2), selected to capture regional variations in diet, milk production rates, and other performance metrics impacting emissions from enteric fermentation, manure, and feed production. Emissions from dairy steers and emissions from breeding practices such as artificial insemination and genetic selections are excluded from the analysis, as dairy steer emissions are attributed to beef production systems and breeding practices are likely negligible and beyond this study’s scope. The analysis estimates the carbon dioxide equivalents (CO_2_e) based on the most recent IPCC Sixth Assessment Report available,^7^ which assumes 100-year global warming potentials (GWP100) of 27 and 30 for biogenic and fossil CH_4_, respectively, and 273 for N_2_O. However, due to limitations in data sources, some emission factors for feed still rely on the previous IPCC AR5 report.^8^

**Figure 2:**
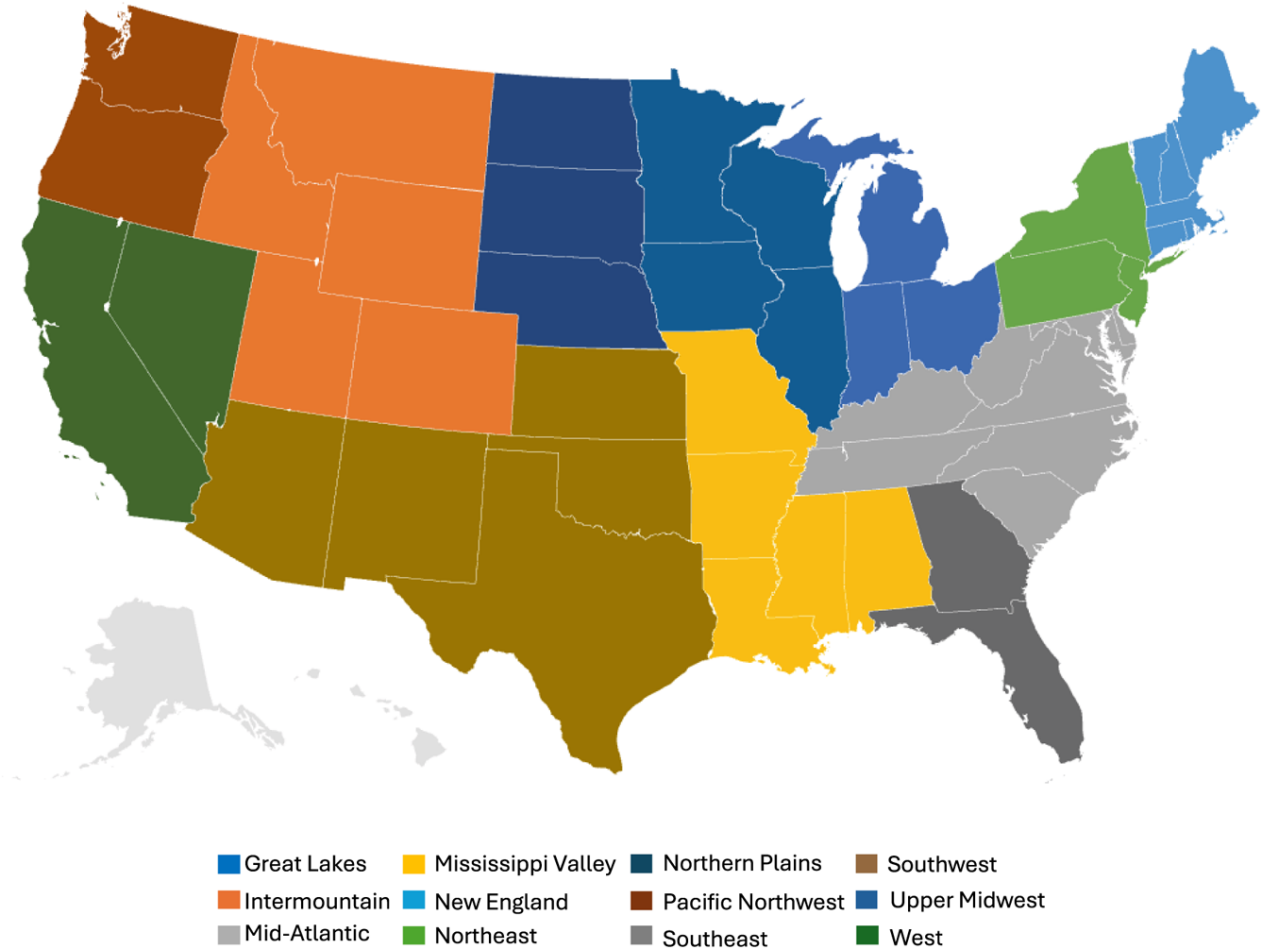
Geographic regions of U.S. dairy production considered in the analysis.

The functional unit for the study is 1 kg of fat- and protein-corrected milk (FPCM), assuming 3.3% protein and 4.0% fat. We estimate regional daily milk production rates using state annual milk production per cow, weighted by the inventory of milk cows per state in each region, and divide by the number of days in a year (Table S1).^9^ We then estimate the total amount of milk produced per cow over its lifetime based on the number of days in lactation, approximately 968 days in 2020 and 1087 days in 2007 (Table S2). The total raw milk produced per cow over its lifetime is converted to fat- and protein-corrected milk (FPCM) based on eq 1 ^10,11^ and the regional milk fat and crude protein content (e.g., kg crude protein/kg milk) data provided by Dairy Management Inc. based on input from industry experts.^12^ The lifetime cradle-to-farm-gate emissions (kg CO_2_e/head) for each cow, as calculated in subsequent sections, are divided by the lifetime FPCM production per cow to determine the region-specific emission intensity (kg CO_2_e/kg FPCM) (see Sections 2.1 through 2.6 for more details). Total absolute emissions are finally estimated based on the total annual quantity of FPCM produced in each region for 2020 and 2007, multiplied by each region’s unique emissions intensity for each respective year.

1 $$\frac{\left(9.29\times \%\mathrm{Fat}\right)+\left(5.47\times \%\mathrm{CP}\right)+0.192}{\left(9.29\times 4\%\mathrm{Fat}\right)+\left(5.47\times 3.3\%\mathrm{CP}\right)+0.192}=\mathrm{FPCM factor}$$

The following sections provide details for estimating the lifetime emissions intensity per head across feed, enteric fermentation, manure, and farm energy.

### 2.1 Feed

Feed diets are estimated across 12 dairy production regions, reflecting typical regional ingredients and balanced to meet daily milk production rates. Diets were formulated using the Nutritional Dynamic System (NDS) Professional software, based on the CNCPS model,^13^ which references NASEM (2021) for 2020 and NRC (2001) for 2007 ^11,14,15^ to predict daily dry matter intake and nutrient requirements for four cattle categories (lactating dairy cows, dry cows, replacement heifers, and bulls). Diet ingredients were based on 2702 survey responses from the Farmers Assuring Responsible Management environmental stewardship program^16^ and literature.^17,18^ Diets are designed to meet nutrient requirements for milk production based on USDA data by state, growth rates, and body weight across cattle categories and include 68 ingredients, with 20 classified as agricultural byproducts. Additional information is provided in Section S2 and Tables S7–S9. Using region-specific diets, we calculated dietary parameters such as neutral detergent fiber (NDF), crude protein (CP), and energy values for each life stage/cattle category, as outlined in Tables S10–S16, to estimate emissions from enteric fermentation and manure.

Feed consumption per region was multiplied by emission factors per feed type, with spatially resolved factors for key feed ingredients (corn, alfalfa, soymeal, etc.) that include direct land use change (LUC) emissions.^19,20^ LUC calculations attribute carbon emissions from land conversion to cropland based on average annual conversion data (2008–2016), tracking land cover change at a 30 m resolution. Pairing this with the associated county-scale net carbon stocks (above, below, and soil organic carbon) lost from converting each land cover category results in annual average committed carbon estimates for producing crops on the newly converted lands, ensuring that regional differences in LUC emissions are integrated into the impacts of dairy production. Consumption-based emission factors integrate production and LUC emissions, weighted by sourcing regions estimated by the FoodS^3^ model, which uses linear optimization to approximate county-level sourcing.^19–21^ Processing emissions from feed derivatives (e.g., DDGS, soymeal) are economically allocated to reflect production motives, aligning with industry standards.^10,22^

For feeds without FoodS^3^ data, we use the freight analysis framework (FAF5) for state-to-state commodity flow data, combined with state-specific emission intensities,^23^ delineated based on differences in grid mixes, yields, and production practices, where available. Regional feed sourcing and emission differences are detailed in Table S17, while some factors use national averages from the GFLI^24^ or other LCA databases^25^ (Table S9). Emission factors for 2007 are adjusted for regional yield differences over the timespan, applying a yield-based ratio to estimate 2007 emissions (Table S18), as yields are the most significant factor changing across the timespan.^1,26^

Approximately 90% of large and medium-scale farms (i.e., operations with more than 500 head) use total mixed ration (TMR) diets, while about 20% of small farms (fewer than 500 head) use TMR.^27^ The proportion of cows in each farm size category is based on the USDA 2017 and 2022 census data to determine the overall average distribution.^9^ TMR diets involve mixing grains and roughage on the farm, primarily using mixer feeder wagons in the U.S. (assuming 90%), with the remaining 10% relying on automatic feeding systems. The energy consumption from TMR mixing is based on Tangorra and Calcante (2018),^28^ whereby mixer feeder wagons require diesel to power the mixers, while automatic feeding systems rely on electricity. For non-TMR diets, we assume that only processed coproduct feeds undergo pelletization at a feed mill before being delivered to the farm, with energy consumption based on Pehlken et al. (2014).^29^ The milling process includes grinding, mixing, and pelletizing, reflecting typical practices required for preparing feed suitable for dairy cattle.

For grazing-based grass inputs, emissions are based on state-scale fertilizer application. Because rangelands are less likely to be intensively managed with fertilization,^100^ fertilizer is attributed only to managed pasturelands, with the distinction based on 30 m pixel classifications from the U.S. Forest Service. Life cycle emissions from fertilizer application are combined with direct and indirect N_2_O emissions based on whether each location applying fertilizer is characterized by a wet or dry climate. Details of the estimation are given in Pelton et al. 2024.^20^

Lastly, emerging research suggests that N_2_O emissions associated with the storage of ensiled feeds such as corn and alfalfa may be significant. This new finding, if confirmed, could represent a substantial source of N_2_O in agriculture;^30^ however, additional research is needed to accurately characterize these emissions. We include initial estimates of N_2_O emissions from ensiled corn and alfalfa as part of the sensitivity analysis in Table S36, based on estimated emissions of 0.69 g N_2_O/kg DM and 0.64 g N_2_O/kg DM, respectively.^30^

### 2.2 Feed Transport

To estimate feed transport emissions, we apply the regional distribution of transport modes based on Freight analysis framework (FAF5) data, which reflects variability in transport practices by truck, rail, barge, and air across regions.^23^ Truck transport represents approximately 76–100% of the total feed transport due to its flexibility and farm access. Rail transport accounts for 1–24%, barge accounts for 1–12%, and air accounts for <1%.^23^ Regional transport data are combined with distances of 100 km for corn grains, hay, and mineral mixes; 80 km for byproduct blends; and 50 km for silage, along with emission factors per mode.^25^

### 2.3 Enteric Fermentation

Accurately modeling GHG emissions from enteric fermentation relies on key dietary and physiological parameters. Studies show that dry matter intake is crucial for broad-scale estimation, but including dietary composition and physiological details improves prediction accuracy.^31^ We utilize the most comprehensive model available, drawing on data from over 1,000 North American dairy cows, which predicts CH_4_ emissions per kg of dry matter intake for lactating dairy cows.^31^ This model (based on eq 65 in Niu et al., 2018) minimizes root-mean-square error (RMSE) by incorporating neutral detergent fiber (NDF) (% of DM), energy-corrected milk (ECM) (kg/day/cow), milk fat (MF), crude protein content (CP) (%), and average body weight (kg) (eq 2).

2 $${\mathrm{CH}}_{4}=13.3+\left(0.118\times \mathrm{NDF}\right)-\left(0.13\times \mathrm{ECM}\right)+\left(2.2\times \mathrm{MF}\right)-\left(1.71\times \mathrm{CP}\right)+\left(0.00521\times \mathrm{BW}\right)$$

For other cattle categories (dry cows, replacement heifers, calves, and bulls), we apply models for the U.S. from Moraes et al. (2014), which minimize RMSE to estimate methane production based on gross energy intake (GEI), crude fat, neutral detergent fiber, and body weight^32^ (eqs S5–S7). Methane energy (MJ) is converted to grams using a factor of 55.65 MJ/kg CH_4_, then divided by daily dry matter intake (DMI) to yield CH_4_ emissions per kilogram of dry matter (DMI).

For each cattle category, we multiply the CH_4_ emissions per kg of DMI by the total dry matter intake per cow, derived from daily intake and the number of days in each life stage (Table S2). Summing emissions across all life stages for cows and bulls provides the total enteric fermentation CH_4_ emissions over each animal’s lifetime.

### 2.4 Manure

To estimate the CH_4_ and N_2_O emissions from manure management, we first calculate the daily volatile solids (VS) and nitrogen excreted for each cattle category. Methane emissions are estimated using degradable volatile solids (dVS), which represent VS minus fecal lignin, as dVS specifically represents the biodegradable portion that converts to methane under anaerobic conditions, thereby improving methane production estimates.

Daily dVS (kg/day) for each cattle category is estimated using Appuhamy et al. (2018) models, which incorporate organic matter (% of DM), NDF (% of DM), and crude protein (% of DM) in the diet for higher accuracy^33^ (eq 3). Total dVS for each growth phase is then calculated based on the time spent in each phase.

3 dVS=0.364×OM+0.029×NDF−0.023×CP−1.017

Nitrogen excretion is calculated using fecal nitrogen (FN) and urinary nitrogen (UN) models from Reed et al. (2015), which incorporate dietary and growth factors as shown in Table 1 (eqs S8–S13), and provide improved nitrogen excretion estimates compared to previous models.^34^

**Table 1:** Parameters Considered for Estimating Urinary and Fecal Nitrogen excretion^34^

Cattle Category	Urinary N	Fecal N
Lactating Dairy Cows	• Nitrogen intake (g/day)	• Metabolizable energy (MJ/day)
	• % Neutral detergent fiber	• % Neutral detergent fiber
	• Digestible energy (MJ/kg DM)	• % Crude protein
	• % Crude protein	• % Forage
	• Days in lactation per head	• Dry matter intake (kg/day)
	• Metabolic body weight (kg)	• Days in lactation per head
Dry Cows	• Nitrogen intake (g/day)	• Nitrogen intake (g/day)
	• Metabolizable energy (MJ/day)	• Metabolizable energy (MJ/day)
	• % Acid detergent fiber	• % Dry matter
	• % Crude protein	• % Acid detergent fiber
	• Metabolic body weight (kg)	• % Ash
		• % Crude protein
Replacement Heifers and Calves		• Metabolic body weight (kg)
Bulls	• Nitrogen intake (g/day)	• Nitrogen intake (g/day)
	• % Crude protein	• Metabolizable energy (MJ/day)
	• Metabolic body weight (kg)	• % Dry matter
		• % Lignin
		• % Ash
		• Metabolic body weight (kg)

GHG emissions are estimated using 2020 state-level manure management systems (MMS) distributions from the U.S. EPA for dairy cows (lactating and dry) and heifers.^35^ For dairy cows, distributions are adjusted to account for the adoption of alternative treatments such as anaerobic digesters, composting, and aerobic treatment. Due to data limitations, national composting and aerobic treatment adoption rates from USDA APHIS surveys^36^ are applied uniformly across states and normalized to 100%. For anaerobic digesters (AD), we use county-level dairy population data from the EPA AgStar database,^37^ estimating remaining non-AD populations by comparing them to the average dairy population data (2007–2017). The non-AD population is then multiplied by the adjusted MMS distribution (considering composting and aerobic treatment) to produce updated distributions for each county and region (Tables S19 and S20). This approach is similarly applied to the 2007 MMS distribution (Tables S21 and S22).

In addition to the primary MMS, several regions employ solid–liquid separation (SLS) technologies to reduce the volume of VS and N processed. Regional SLS adoption rates (10–15%) and technology distributions were estimated based on Greene et al. (2024)^38^ and expert input^12^ (Table 23). Typically, SLS is applied before materials enter uncovered lagoons or liquid/slurry systems, but in AD systems, 80% of SLS is used postdigestion and 20% predigestion. SLS efficiency in removing VS and N depends on the technology (Table S24). For SLS implemented before the primary MMS, separated VS and N are directed to solid storage MMS; for AD systems using SLS postdigestion, separated VS is also directed to solid storage, while residual material is managed in lagoon effluent ponds for covered lagoon AD systems and through liquid/slurry systems for all other AD systems (e.g. complete mix, plug flow, and induced blanket reactor). In cases where no SLS is deployed, digestate from covered lagoon AD systems is similarly directed to lagoon effluent ponds, with digestate from all other AD systems managed in liquid/slurry systems.

Methane emissions are calculated by multiplying dVS per head by the maximum methane-producing capacity, *B*_0_ (kg of CH_4_/kg of VS) and the methane conversion factor for each MMS, influenced by system type and regional ambient temperatures^63^ (Table S25). For most MMS, dairy cows have a *B*_0_ of 0.14 m^3^CH_4_/kg VS and other cattle categories have 0.11 m^3^ CH_4_/kg VS, while AD systems produce 90% of maximum capacity.^35,39,40^ Total CH_4_ emissions are converted from m^3^ to kg using methane’s density (0.662 kg/m^3^).

Methane leakage from AD systems is estimated based on collection and destruction efficiencies, assuming 99% collection for plug flow, mixed, and induced blanket digester systems and 86.3% for covered lagoon systems, reflecting both bank-to-bank impermeable systems (97.5% collection efficiency) and modular impermeable systems (75% collection efficiency).^41^ Combustion efficiency is assumed to be 98%,^41^ with total leakage calculated as uncollected methane plus methane not destroyed during combustion.

N_2_O emissions from manure management occur in addition to methane, arising directly from microbial denitrification or indirectly from ammonia (NH_3_) and other nitrogen oxides (NOx). To estimate direct and indirect N_2_O emissions, we calculate the total nitrogen excretion across each life phase.

Total nitrogen excretion is combined with direct N_2_O conversion rates for each manure management system (Table S26). For indirect N_2_O emissions, we estimate the fraction of total urinary nitrogen that volatilizes as NH_3_ based on the manure management system (Table S27) and apply a conversion factor of 0.01 kg N_2_O–N/kg N volatilized.^39^ For indirect N_2_O emissions from runoff and leaching, we estimate the nitrogen losses across five U.S. regions, using a conversion factor of 0.0075 kg N_2_O–N/kg N lost,^39^ then apply the molar mass ratio (44 kg N_2_O/28 kg N) for total N_2_O.

Under anaerobic digestion, N_2_O emissions are negligible per IPCC guidance and are excluded. Land-applied manures to cropland systems are also excluded, as they are part of crop production emissions.^10^ N_2_O emissions from pasture, range, and paddock (PRP) systems are, however, included.^42^

We also account for methane from manure scraping, flushing, and vacuuming from dairy barns, which exclude pasture, range, paddock, and dry lot systems. Using county MMS distributions, ambient temperatures^63^, barn floor areas per animal (assuming 9.25 m^2^ for cows/bulls; 8.75 m^2^ for heifers/calves), and a scalar of 0.13 (eq S14),^40^ we estimate daily methane per head, which is multiplied by the number of days in each life phase.

To calculate total CO_2_e emissions from manure handling and storage, we multiply CH_4_ and N_2_O per head in each phase by their GWP factors, summing these for total CO_2_e per animal over its lifetime. Regional CO_2_e averages are then weighted by the dairy cow inventory in each county.

### 2.5 Dairy Farm

To estimate energy use on dairy farms, we apply a meta-analysis of thermal and electrical energy per kilogram of energy-corrected milk (ECM) for conventional and pasture-fed systems.^43^ We allocate electrical energy use between milk and meat products according to the proportion of electricity used for various operations, including water heating, milk cooling, milk harvesting, water pumping, and other miscellaneous uses, such as lighting.^43^ We attribute only the energy associated with miscellaneous uses (approximately 12% of total energy use) to both meat and dairy products, while the remainder is attributed solely to milk products. Total energy use per cow is calculated using daily ECM output, total lifetime before culling, and the proportion of cows in conventional versus PRP systems, derived from the state-scale manure management data (Figure S1). Emissions from energy use are estimated using life cycle emission factors.^25^ For electricity, area-weighted average emission factors are determined for each of the 12 dairy regions based on EPA eGRID subregion data using 2020 fuel mixes for the 26 eGRID subregions, including combustion, upstream extraction, and transmission losses.^25,44^

In addition to the energy use in conventional dairy systems, we also estimate the additional energy required to operate AD systems. Thermal energy for AD systems depends on the heat needed to maintain operating temperatures compared to ambient temperatures,^38^ estimated for covered lagoon systems and all other AD systems by eq S15. Electricity use for AD systems is based on GREET (2023).^45^ Details are provided in Table S28. When biogas is upgraded to renewable natural gas (RNG) or compressed natural gas (CNG), specific electricity energy demands and embedded emissions for compression and methanation are applied, with distribution emissions based on GREET.^45,46^

A portion of collected methane is used for onsite energy, with total use depending on biogas end use. AgStar data includes information on biogas applications, including flaring, onsite energy use, grid sales, or RNG and CNG upgrading.^37^ For digesters generating electricity for the grid (at 35% efficiency), we assume that a portion is used onsite, with grid displacement credits based on marginal emissions for the relevant eGRID subregion using the 2020 nonbaseload fuel mix.^44^ As turbines are used for electricity generation and not for thermal energy, the remaining onsite energy needs are assumed to be provided by natural gas purchases burned in boilers at 80% efficiency. For biogas used for thermal energy generation, a portion covers onsite energy demands (assuming 80% boiler efficiency), with the surplus displacing natural gas at colocated facilities. For thermal energy generators, digester electricity needs are assumed to be grid-sourced at eGRID emission rates. For digesters cogenerating electricity and thermal energy in combined heat and power (CHP) reciprocating engines (with a 44% thermal efficiency and a 35% electrical efficiency), we assume that a portion of the generated energy is used onsite, with the surplus sold to the grid or used to satisfy colocated facility demands.^47^ For digesters producing RNG or CNG, we assume that all energy inputs are purchased. While RNG receives displacement credits equivalent to the life cycle emissions from combusting natural gas, CNG is assumed to displace an energy-equivalent unit of diesel and gasoline vehicles, based on the portion of CNG vehicle miles traveled in light-duty vehicles (LDV – gasoline-powered) versus heavy-duty vehicles (HDV – diesel-powered) and the associated miles per gallon gasoline equivalence in LDV and HDV.^45,48^ For digesters flaring biogas, onsite energy needs are met with natural gas and grid electricity.

### 2.6 Allocation

Lifetime emissions from bull production are allocated to dairy cows based on a breeding ratio of 100 cows per bull, assuming 25 cows per bull per season and four breeding seasons.^1^

We then allocate dairy cow emissions between milk and meat production based on the relative biophysical energy requirements for milk production versus muscle (meat) production over the cow’s lifetime. Biophysical allocation is based on the total annual net energy for growth (MJ/kg liveweight) based on the IDF (2022) guidelines^10^ and total energy for lactation (MJ/kg FPCM)^11^ (eqs S1–S4). The estimated allocation of impacts attributed to milk in 2020 is 81% and 79% in 2007 (Table S6), which is consistent with other milk LCA studies.^49,50^

For manure, we apply a cutoff approach where emissions from manure management excluding field application are attributed to animal production, while emissions from field application are assigned to crop production, following FAO LEAP and IDF guidance.^10,22^

### 2.7 Sensitivity and Uncertainty Analysis

Methodological choices, assumptions, models, and data sources in LCA studies introduce variability and uncertainty in emissions estimates, influencing climate action priorities. The primary categories of uncertainty include parameter uncertainty, scenario uncertainty, and model uncertainty, each of which can be evaluated using various methods.^51,52^ Parameter and model uncertainty can be examined using methods such as parameter variation, sampling techniques (e.g., Monte Carlo or Latin hypercube simulations), and Bayesian statistics. These approaches help illustrate how uncertainties in input and model variables affect the range of possible outcomes. Scenario uncertainty, along with certain aspects of model uncertainty, can be explored using sensitivity analyses to assess the stability and potential range of outcomes under different scenarios.^52,52^ In our study, we apply discrete sensitivity analysis to investigate how methodological choices, such as selecting GWP factors (*scenario uncertainty*) and using alternative modeling frameworks for enteric fermentation and manure excretion (*model uncertainty*), influence overall emissions estimates.

For example, the estimation of carbon-equivalent emissions has evolved with advancements in climate science, affecting total CO_2_e emissions for the dairy sector. Understanding these variations helps stakeholders contextualize results relative to past studies. Thoma et al. (2013) used the IPCC Fourth Assessment Report (AR4),^3^ Rotz et al. (2021) used AR5 without climate-carbon feedback,^4^ Capper and Cady (2020) included both AR4 and AR5 with climate-carbon feedback,^1^ and the U.S. EPA (2022) continues to use AR4.^53^ Our study is the first to implement AR6 models for U.S. dairy cradle-to-farm-gate emissions; however, due to data limitations, certain feed emission factors rely on AR5 with climate-carbon feedback, as detailed in Table S31.

In addition to scenario uncertainty associated with GWP selections, we use sensitivity analyses to address model uncertainty, which arises from inherent limitations in modeling approaches that aim to represent real-world processes. The choice of models can introduce substantial variability.^39,54^ To capture this, we evaluate emissions estimates’ sensitivity by applying alternative models for enteric fermentation, manure VS, and N excretion. These models, used in previous LCA and GHG inventory studies,^1,2,4,6,35^ are applied using our study’s specific feed, growth, and performance parameters to explore potential variation in emissions estimates under alternative model selection scenarios (eqs S16–S30).

Moreover, uncertainties in both models and parameters impact emissions estimates,^55^ due to inherent standard errors and incomplete knowledge about key process parameters’ true values. For parameter uncertainty, such as variations in feed intake, emission factors, and model error, we use parameter variation methods combined with a bounded error propagation approach. We selected this method rather than more complex approaches such as Monte Carlo simulation due to limitations in our model structure. Using bounded error propagation, we present a plausible range of outcomes by combining parameter extremes, selecting either all lower-bound or all upper-bound values. Although this approach does not yield a full probability distribution of total emissions, it offers a clear and transparent illustration of the potential span of cradle-to-farm-gate emissions if all examined uncertainty parameters were to simultaneously skew high or low. Specifically, our analysis investigates how variations in the coefficient of variation in the enteric fermentation methane model and the manure management models (VS and N excretion), as detailed within each study, impact total GHG estimates. Additionally, we evaluate uncertainty in feed emissions by considering parameter uncertainty in dry matter intake (assuming ±10% variation) and in the emission intensity of feed inputs, using the standard deviation of emissions per dairy region and feed type. For national average feed emission factors, we apply the average standard deviation across the regionalized feed types.

## 3 Results and Discussion

### 3.1 Absolute Emissions and Contribution by Source

Total U.S. cradle-to-farm-gate GHG emissions attributed to milk production in 2020 are estimated to be 138.88 million metric tonnes (MMT) of CO_2_e on average, with 32.05 MMT attributed to the beef system. Concentrate feed production (grains and byproducts) contributes the largest share of emissions, at 48.1 MMT CO_2_e, or 35% of total emissions, with regional variations from 27% to 49% (Figures 3 and 4). This includes emissions from multiple feed sources influenced by regional production practices, environmental conditions, and sourcing. The high contribution from concentrate feeds in part reflects the inclusion of LUC estimates for 68 feed products, elevating emissions beyond prior estimates. Feed-related emissions, including concentrate, forage, milling/mixing, and transport, account for nearly twice the emissions of enteric fermentation but arise from multiple fragmented sources along the supply chain, making management more complex and necessitating significant coordination across multiple regions, crops of production, and various points along the supply chain. In contrast, enteric fermentation contributes about 38.1 MMT CO_2_e, or 27% of total emissions (23–33% range across regions), representing the largest single source of emissions. This emphasizes the importance of targeting methane emissions from digestive processes to meet net GHG neutrality targets.

**Figure 3:**
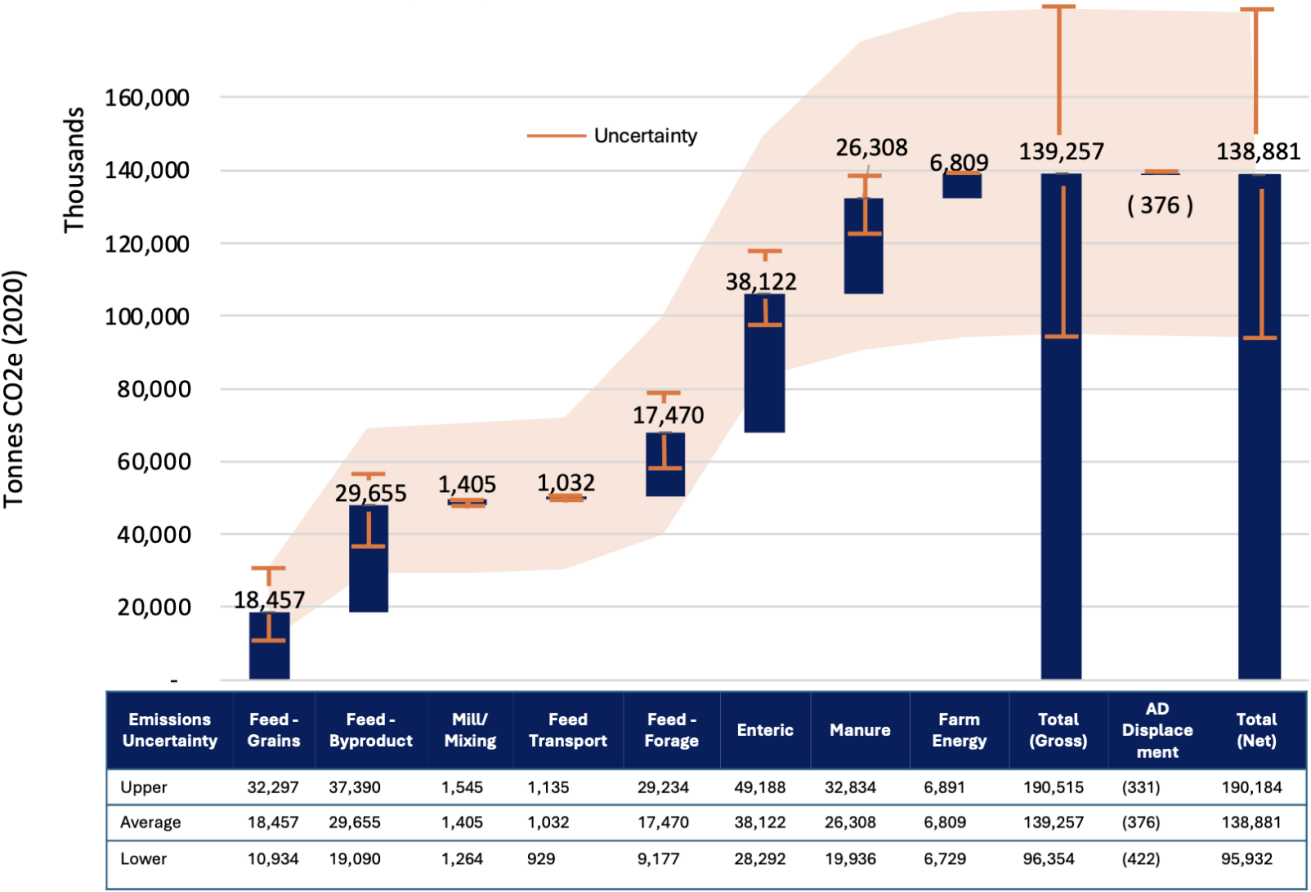
Total cradle-to-farm-gate greenhouse gas emissions from milk production across emission sources and uncertainty range. ^a^Considers standard error uncertainty in enteric methane formation, VS and N excretion models, and standard deviation estimates for feed emission factors and dry matter intake.

**Figure 4:**
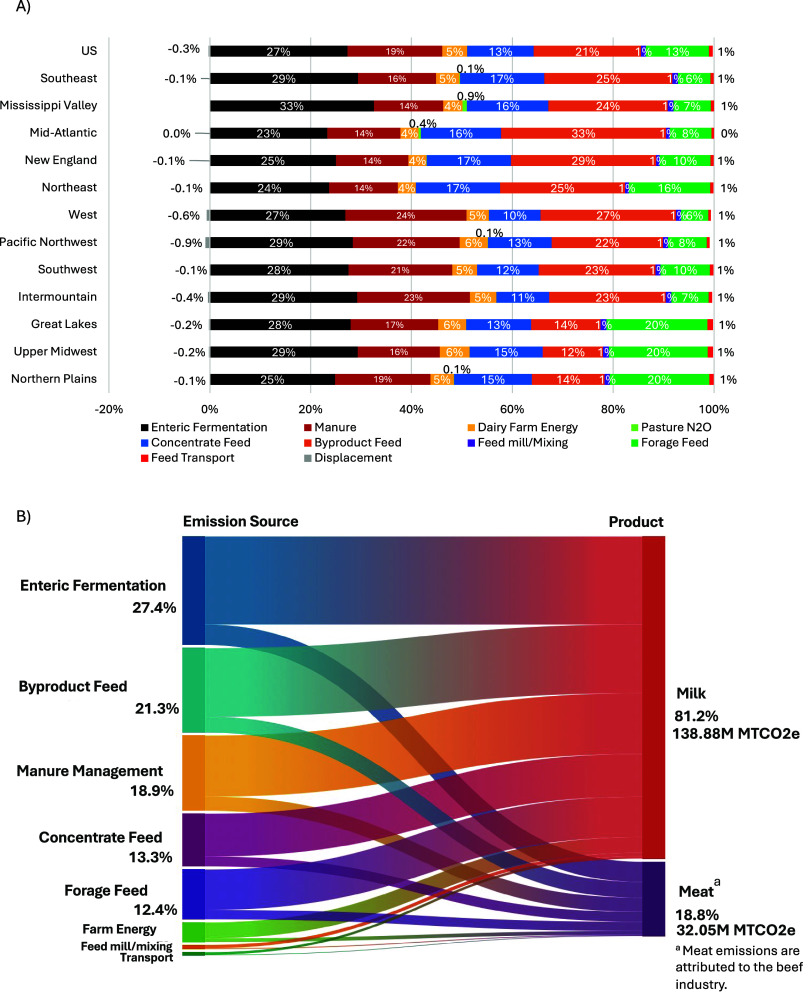
Regional contribution of emission sources (see Figure S4 for uncertainty) (A), and contribution of cradle-to-farm-gate emissions across sources and products (B).

While LUC had previously been excluded from analyses, as it was assumed not to be a major contributor within the U.S., the 2022 International Dairy Federation (IDF) guidelines,^10^ along with other key protocols such as the GHG Protocol Product Standard^51^ and FAO LEAP guidance^22^ now include LUC as a critical component in emissions reporting and assessments. This reflects the increased recognition of LUC’s contribution to total emissions and the necessity for comprehensive accounting in environmental analyses. This study underscores its substantial contribution in the context of feed crops used by the dairy industry, averaging 11% of total dairy emissions with regional ranges of 6–16%.

Manure emissions, from both CH_4_ and N_2_O, total approximately 26.3 MMT CO_2_e, or 19% of the total emissions (14–24% across regions). Methane represents about 73% of manure emissions, with 27% from N_2_O on average, ranging between 51% and 81% of total emissions across regions due to differences in management systems. Regions with higher use of pasture/range/paddock (PRP) systems, like the Mississippi Valley, have higher-than-average N_2_O emissions, while areas with more anaerobic lagoons, like the Northeast, have manure emissions driven mostly by CH_4_. Anaerobic digesters with biogas capture and utilization provide emissions offset credits, averaging 0.3% across the industry and reaching up to 0.8% in high-adoption areas. Emissions from forage feeds, farm energy, feed mills/mixing, and feed transport represent 13%, 5%, 1%, and 0.7%, respectively.

In 2020, U.S. cradle-to-farm-gate GHG emissions from the dairy sector were largely driven by biogenic methane, contributing approximately 41% of the total (Figure 5 and Table S29). Emissions from feed inputs (i.e., “aggregate CO_2_e”) follow closely but could not be broken down by specific GHGs due to data limitations; these likely include CO_2_ from manufacturing inputs (e.g., fertilizers and pesticides), fuel combustion, lime applications, LUC, and N_2_O from field-applied fertilizers. The distributions between CO_2_ and N_2_O likely differ across byproduct feeds. For example, for DDGS feed, CO_2_ represents around 93% of total emissions and N_2_O represents 1%, whereas for soybean meals, CO_2_ represents 50% of total emissions and N_2_O represents 42%.^25,56^ N_2_O (from manure and field-applied fertilizers) and LUC each account for about 11% of total dairy emissions, while fossil fuel CO_2_ from crop and dairy farm operations contributes around 6%. Feed practices that increase soil carbon storage, such as transitioning from intensive till to no-till practices and the addition of cover crops, lead to a baseline reduction of net emissions by approximately 2.0 MMT CO_2_e, or 1% of the total emissions.

**Figure 5:**
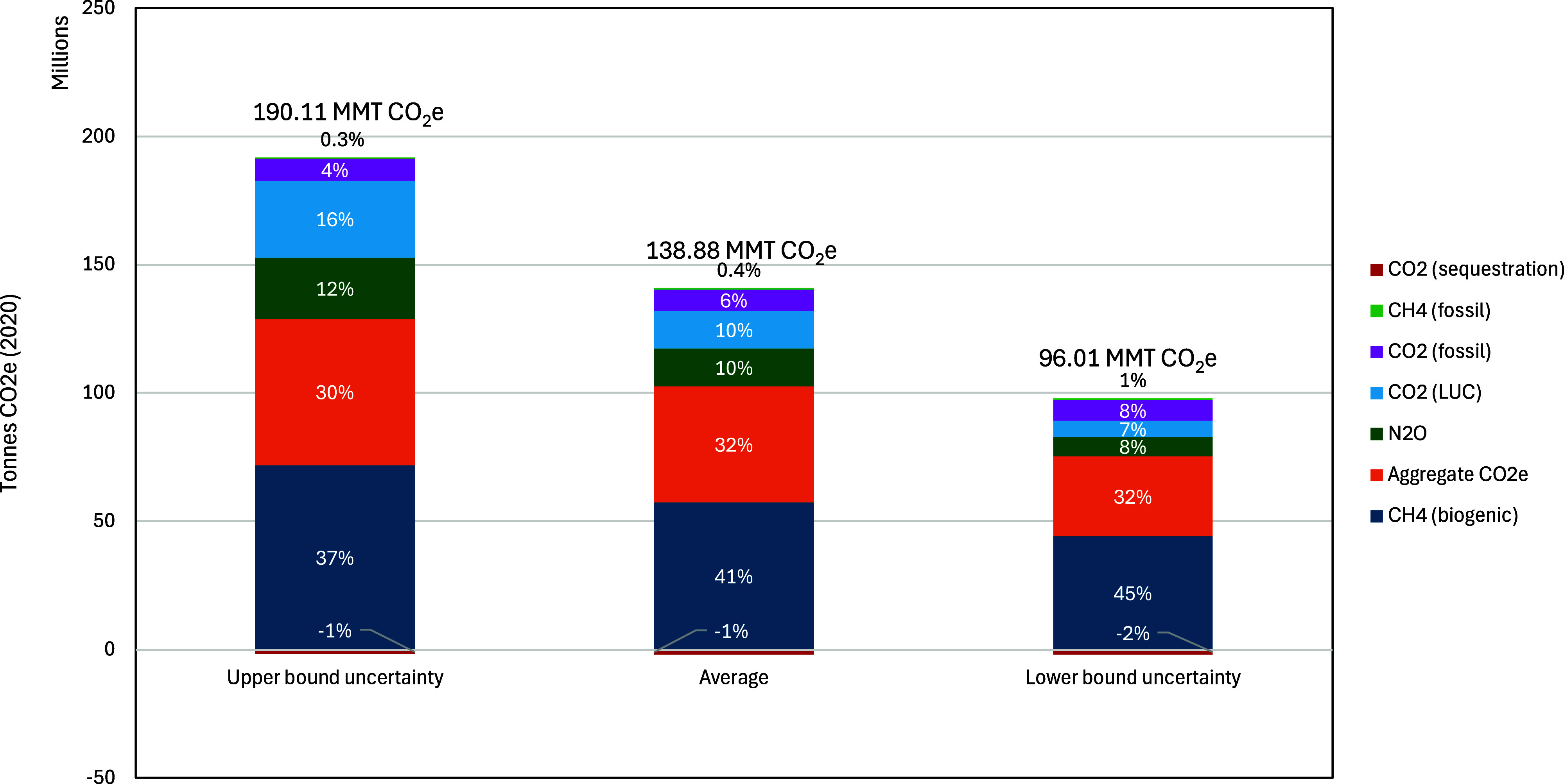
Contribution of greenhouse gases to 2020 U.S. dairy emissions (in CO_2_e). Note that aggregate CO_2_e cannot be disaggregated due to data limitations, however, because these emissions are from feed inputs, GHGs are likely to be split mostly between CO_2_ and N_2_O categories. ^a^Considers standard error uncertainty in enteric methane formation, VS and N excretion models, and standard deviation estimates for feed emission factors and dry matter intake.

### 3.2 Emission Intensity and Benchmarking Comparisons

The average U.S. emissions intensity allocated to milk is estimated at 1.38 kg CO_2_e per kg of FPCM, with regional variations ranging from 1.24 to 1.87 kg CO_2_e per kg of FPCM (Table S30). These differences stem from factors such as feed diets, sourcing regions, manure management, and milk production rates, where lower milk yields increase emissions intensity per kilogram of FPCM. Notably, the Upper Midwest and Western regions, which produce the greatest quantities of milk across the U.S., have lower-than-average emissions intensities, while regions with lower milk production are consistent with higher intensities. Some of these regions also exhibit higher prevalence of pasture/range/paddock grazing systems, however, which may be desired by certain dairy buyers.

The emissions range estimated in this study aligns with previous assessments, which reported values from 0.69 to 1.84 kg CO_2_e/kg FPCM.^1,4–6,57–61^ Although direct comparisons are complex due to differences in assumptions, data sources, model structures, and changes in industry practices over time, we isolated key components for comparative analysis. Figure 6a presents our sensitivity analysis, exploring the effects of alternative models for estimating VS, N excretion, and enteric fermentation. Using these alternative models, total 2020 emissions for U.S. dairy range from 124.5 to 156.9 MMT CO_2_e, with an emission intensity of 1.24 to 1.56 kg CO_2_e/kg FPCM, depending on the models used. Detailed comparisons between models are shown in Section S7, Tables S32, S33 and Figure S2. These results highlight how model choice impacts emissions estimates, underscoring the importance of using models that reflect the latest advancements in emission estimation and the potential limitations of harmonization when relying on outdated or less accurate models.

**Figure 6:**
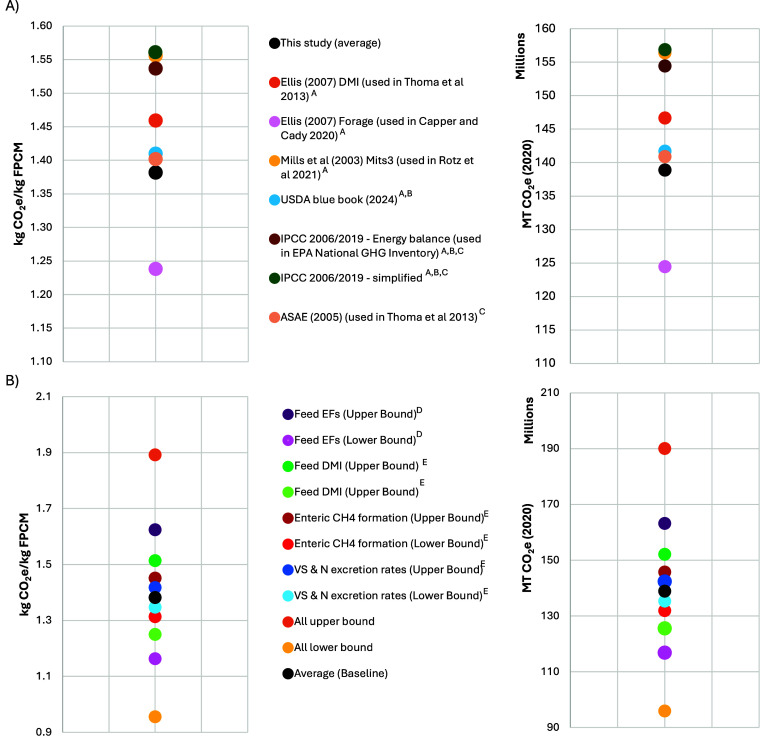
Sensitivity analysis of 2020 GHG emissions intensity and absolute GHG emissions considering (A) alternative models for estimating enteric methane and manure VS and N excretion rates and associated emissions, and (B) uncertainty (characterized by standard error and deviation) in key parameters, including feed emission factors, feed dry matter intake, enteric methane formation, and manure volatile solids and N excretion rates. ^A^Based on alternative enteric fermentation models. ^B^Based on alternative enteric fermentation and manure VS and N models. ^C^Based on alternative manure N models. ^D^Based on standard deviation of regional emission factors across feed types. ^E^Based on standard error in models.

In addition to sensitivity in model selection, our analysis includes an estimate of uncertainty within feed, enteric methane, and manure emissions sources, with uncertainty bounds determined based on the standard errors of prediction models (e.g., enteric methane, VS, and N excretion) and the standard deviation across regional data inputs (e.g., feed emission factors). Figures 3 and 6b indicate that our estimates range between 96 million MMT and 190 MMT CO_2_e or 0.96 and 1.89 kg CO_2_e/kg FPCM, when accounting for all evaluated sources of uncertainty (see Figure S3 for regional ranges). Figure 6b and Tables S34 and S35 present uncertainty ranges for each emission source, showing that the uncertainty in the selected manure VS and N excretion models has a smaller effect on the range in total emissions than the uncertainty in the selected enteric methane model. A ±10% uncertainty in dry matter intake (DMI) results in a corresponding 10% change in total emissions, as DMI directly scales emissions from feed inputs, enteric fermentation, and manure. Emission estimates are most sensitive to uncertainty in feed emission factors, highlighting the importance of detailed data to capture the spatially heterogeneous impacts of feed production combined with the need for enhanced supply chain transparency to identify relevant sourcing regions.

Using recent models and spatial data offers a more precise view of current dairy practices and associated emissions, although uncertainty remains. Enteric fermentation and manure continue to be significant, averaging 46% of total farm-gate emissions, though lower than previous estimates (see Figure S4 for ranges across regions and uncertainty). A key difference in this study is our inclusion of LUC emissions, historically assumed to be minimal in the U.S. Our findings, however, show LUC as a notable contributor, and addressing it is necessary to meet climate action targets. While the dairy industry drives demand for feed crops, among other industries, it accounts for only 9%, 1%, and 5% of total land conversion for corn, soy, and wheat, respectively.^19^

Table S36 examines the effects of excluding LUC or biogas displacement credits and including N_2_O emissions from silage storage. Table S37 further assesses how emissions vary under different GWP factors from IPCC Assessment Reports (AR4, AR5 with and without climate carbon feedback, and AR6). The analysis reveals notable variations in biogenic CH_4_ emissions; under AR6, biogenic CH_4_ contributes 57.4 MMT CO_2_e (41% of total emissions), while AR5 with climate carbon feedback increases this to 71.8 MMT CO_2_e (47%). This sensitivity to characterization factors highlights the importance of selecting appropriate factors when benchmarking emissions. Across the four GWP characterization methods, total emissions range from 134.9 MMT CO_2_e (AR4) to 154.1 MMT CO_2_e (AR5 with climate-carbon feedback), illustrating the significant impact that methodological choices can have on emission estimates.

Figure 7 shows our final analysis comparing the 2020 emissions with updated estimates for 2007. This comparison revisits the 2007 data using the latest models, system boundaries, and consistent assumptions, offering a more reliable basis for assessing changes over time rather than directly comparing with previous assessments.^2^ The analysis indicates that the emission intensity has decreased from an average of 1.59 kg CO_2_e/kg FPCM in 2007 to 1.38 kg CO_2_e/kg FPCM in 2020, reflecting improved efficiency and emissions management. However, despite this reduction in intensity, total emissions have risen from 127 million tonnes of CO_2_e in 2007 to 139 million tonnes in 2020, driven by a substantial rise in total milk production from 79 to 101 million tonnes of FPCM. This demonstrates progress in emission reductions per unit output but also underscores challenges posed by increased production for achieving GHG neutrality targets. The updated comparison to the 2007 baseline offers a consistent and refined view, providing valuable insights that cannot be captured through direct comparisons with earlier studies due to differences in their methodologies and assumptions.

**Figure 7:**
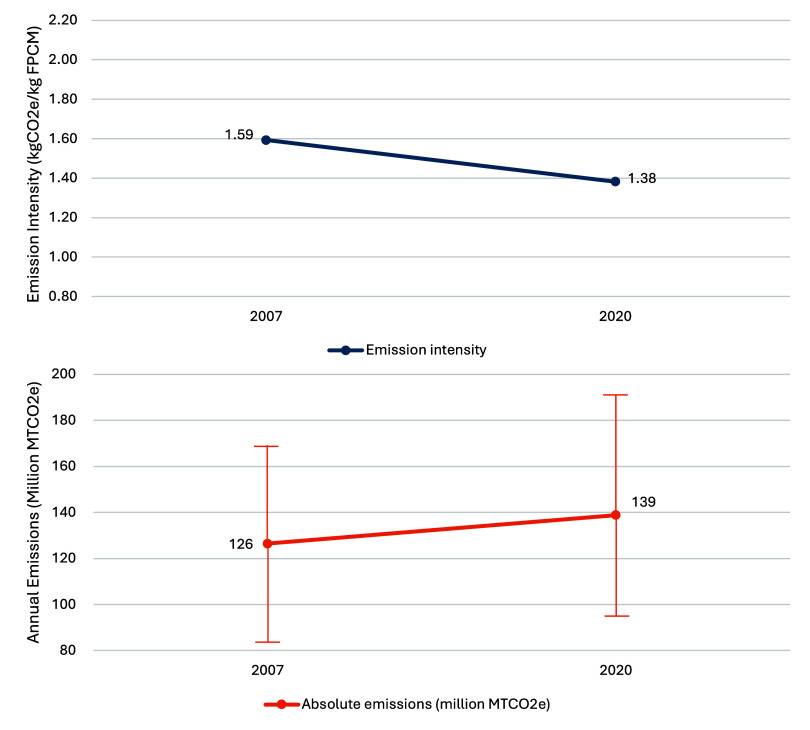
Comparing the emission intensity and annual total emissions in 2020 (with 101 million tonnes of FPCM) to 2007 (with 79 million tonnes of FPCM), with uncertainty bounds (see Supplementary Table S38). ^a^Considers standard error uncertainty in enteric methane formation, VS and N excretion models, and standard deviation estimates for feed emission factors and dry matter intake.

As the dairy industry pursues its 2050 GHG neutrality goal, the variability in emissions estimates across different models and assumptions highlighted in this study provides critical insights for the dairy industry to understand the implications of establishing a robust baseline for current emissions. To guide policy development and strategic planning efforts to achieve long-term sustainability goals, flexible LCA models are needed that allow for parametrized comparisons of alternative practices and can incorporate updated models and data upon availability, ensuring consistency in assumptions and system boundaries. By doing so, we can more accurately characterize mitigation potentials and avoid the noise and errors introduced by using disparate, nonintegrated data sets. While this study provides a comprehensive framework for assessing GHG emissions from dairy production, several limitations and areas requiring further research must be acknowledged. As noted earlier, some feed emission factors used in the analysis are based on GWP values from the IPCC AR5 report, and future work should align these with the updated AR6 GWP values to ensure consistency with the latest climate science. Additionally, methods for estimating LUC continue to evolve as improved ground-truth data become available, enabling better calibration and validation of LUC models. Refining spatial estimates of feed production impacts, particularly for feed ingredients less commonly studied, and enhancing the precision of assumptions regarding sourcing regions represent another critical area for further development. Beyond feed, future research could also address spatial differences arising from breed-specific characteristics, regional variations in animal body weight, and the influence of farm size on emissions. Addressing these limitations highlights the need for ongoing research and iterative methodological improvements to enhance the accuracy, regional specificity, and policy relevance of dairy LCA methods. Despite these limitations and the need for ongoing research, this study meets all of the ISO data quality criteria established as part of the goal of the study (see Table S39).

This study offers a comprehensive and spatially resolved assessment of cradle-to-farm-gate greenhouse gas emissions from the U.S. dairy industry. By integrating the latest climate science, diverse data sources, and advanced modeling, this work offers refined estimates of key emission sources, such as enteric fermentation, manure management, and feed production, emphasizing the importance of methodological consistency and regional specificity. This study serves as the foundation for an extended assessment that will examine the impacts of all dairy products from cradle to processing gate. Moreover, it establishes a baseline for understanding current emissions, which is crucial for developing and implementing strategies to achieve the industry’s 2050 net GHG neutrality targets.

## Supplementary Material



## Notes

Includes additional details and assumptions related to growth and performance, feed, enteric fermentation, manure management, and farm energy; uncertainty and sensitivity analyses, benchmark comparisons, and data quality (PDF)

## References

[1] Capper J., Cady R. (2020). The effects of improved performance in the U.S. dairy cattle industry
on environmental impacts between 2007 and 2017. J. Animal Sci..

[2] Thoma G., Popp J., Shonnard D., Nutter D., Matlock M., Ulrich R., Kellogg W., Kim S., Neiderman Z., Kemper N. (2013). Regional analysis of
greenhouse gas emissions from
USA dairy farms: A cradle to farm-gate assessment of the American
Dairy industry circa 2008. Int. Dairy J..

[3] Thoma G., Popp J., Nutter D., Shonnard D., Ulrich R., Matlock M., Kim D., Neiderman Z., Kemper N., East C. (2013). Greenhouse
gas emissions
from milk production and consumption in the United States: A Cradle-to-grave
life cycle assessment circa 2008. Int. Dairy
J..

[4] Rotz A., Stout R., Leytem A., Feyereisen G., Waldrip H., Thoma G., Holly M., Bjorneberg D., Baker J., Vadas P. (2021). Environmental
assessment
of United States dairy farms. J. Cleaner Prod..

[5] Rotz C., Stout R., Holly M., Kleinman P. (2020). Regional environmental
asesssment of dairy farms. J. Dairy Sci..

[6] Henderson A., Asselin-Balencon A., Heller M., Burek J., Kim D., Lessard L., Margni M., Saad R., Matlock M., Thoma G. (2023). Spatialized life cycle assessment of fluid milk production
and consumption in the United States. Sustainability.

[7] IPCC. Climate change 2021: the physical science basis; IPCC, 2021.

[8] IPCC. Climate change 2013: The physcial science basis. IPCC. 2013.

[9] USDA ERS, Dairy Data. 2024, https://www.ers.usda.gov/data-products/dairy-data/. [Accessed 3 January 2025].

[10] The IDF global Carbon Footprint standard for the dairy sector. The IDF global Carbon Footprint standard for the dairy sector, Bulletin Of The International Dairy Federation, 2022.

[11] NASEM. Nutrient Requirements for Dairy Cattle, 8thed; National Academies Press: 2021. 38386771

[12] Dairy Management Inc Personal Communications. Dairy Manage ment Inc. 2025.

[13] Van
Amburgh M., Collao-Saenez E., Higgs R., Ross D., Recktenwald E., Raffrenato E., Chase L., Overton T., Mills J., Foskolos A. (2015). The Cornell Net Carbohydrate and
Protein System: Updates to the model and evaluation of version 6.5. J. Dairy Sci..

[14] NRC. Nutrient Requirements of Dairy Cattle; National Academy Press: Washington, DC, 2001.

[15] Van
Amburgh M., Collao-Saenz E., Higgs R., Ross D., Recktenwald E., Raffrenato E., Chase L., Overton T., Mills J., Foskolos A. (2015). The Cornell Net Carbohydrate and
Protein System: Updates to the model and evaluation of version 6.5. J. Dairy Sci..

[16] FARM, Farmers Assuring Responsible Management Environmental Stewardship Survey, https://nationaldairyfarm.com. [Accessed 3 January 2025].

[17] de
Ondarza M., Tricarico J. (2021). Nutritional contributions and non-CO2
greenhouse gas emissions from human-inedible byproduct feeds consumed
by dairy cows in the United States. J. Cleaner
Prod..

[18] Asselin-Balencon A., Popp J., Henderson A., Heller M., Thoma G., Jolliet O. (2013). Dairy farm greenhouse
gas impacts: A parsimonius model
for a farmer’s decision support tool. Int. Dairy J..

[19] Pelton R. E., Spawn-Lee S. A., Lark T. J., Kim T., Springer N., Hawthorne P., Ray D. K., Schmitt J. (2021). Land use leverage
points
to reduce GHG emissions in U.S. agricultural supply chains. Environ. Res. Lett..

[20] Pelton R., Kazanski C., Keerthi S., Racette K., Gennet S., Springer N., Yacobson E., Wironen M., Ray D., Johnson K., Schmitt J. (2024). Greenhouse
gas emissions in US beef
production can be reduced by up to 30% with the adoption of selected
mitigation measures. Nat. Food.

[21] Smith T., Goodkind A., Kim T., Pelton R., Suh K., Schmitt J. (2017). Subnational mobility
and consumption-based environmental
accounting of US corn in animal protein and ethanol supply chains. Proc. Natl. Acad. Sci. U. S. A..

[22] Food and Agriculture Organization, Environmental performance of large ruminant supply chains: Guidelines for assessment. https://openknowledge.fao.org/items/143aa579-884c-4c0e-8bbd-3a6cc7866636. [Accessed 3 January 2025].

[23] Oakridge national Laboratory, Freight Analysis Framework Version 5. 2024 https://faf.ornl.gov/faf5/. [Accessed 3 January 2025].

[24] GFLI. Global Metrics for Sustainable Feed 2024. https://globalfeedlca.org. [Accessed 5th October 2024].

[25] Sphera. LCA for Experts Software. 2024. https://about.sphera.com/. [Accessed 3 January 2025].

[26] USDA. Agricultural Census Fertilizer Use and Price; United States Department of Agriculture, 2019.

[27] Schingoethe D. (2017). A 100-yr review:
Total mixed ration feeding of dairy cows. J.
Dairy Sci..

[28] Tangorra F., Calcante A. (2018). Energy consumption and technical-economic analysis
of automatic feeding system for dairy farms: results from a field
test. J. Agric. Eng. Res..

[29] Pehlken, A. ; Kirchner, A. ; Thoben, K. . Manufacturing with minimal energy consumption - a product perspective. In Technology And Manufacturing Process Selection-the Product Life Cycle Perspective; Springer, 2014, 175–191. 10.1007/978-1-4471-5544-7_9

[100] Toledo D., Sanderson M., Goslee S., Herrick J., Fults G. (2016). An integrated grazing
land assessment approach for range and pasturelands. Journal of Soil and Water Conservation.

[30] Yang S., Mahmood M., Baral R., Wu H., Almloff M., Stanton L., Min D., Smiley B., Iiams C., Yu J. (2024). Forage conservation
is a neglected nitrous oxide source. PNAS Nexus.

[31] Niu M., Kebreab E., Hristov A., Oh J., Arndt C., Bannink A., Bayat A., Brito A., Boland T., Casper D. (2018). Prediction of enteric
methane production, yield, and
intensity in dairy cattle using an intercontinental database. Global Change Biol..

[32] Moraes L., Strathe A., Fadel J., Casper D., Kebreab E. (2014). Prediction
of enteric methane emissions from cattle. Global
Change Biol..

[33] Appuhamy J., Moraes L., Wagner-Riddle C., Casper D., Kebreab E. (2018). Predicting
manure volatile solid output of lactating dairy cows. J. Dairy Sci..

[34] Reed K., Moraes L., Casper D., Kebreab E. (2015). Predicting nitrogen
excretion from cattle. J. Dairy Sci..

[35] US EPA. Inventory of U.S. Greenhouse Gas Emissions and Sinks: 1990–2020: Annex 3. United States Environmental Protection Agency. 2022.

[36] USDA. Dairy 2014: Dairy cattle management practices in the United States, 2014, USDA. 2016.

[37] EPA ″Livestock Anaerobic Digester Database. 2024 https://www.epa.gov/agstar/livestock-anaerobic-digester-database. [Accessed 3 January 2025].

[38] Greene J., Wallace J., Williams R., Leytem A., Bock B., McCully M., Kaffka S., Rotz A., Quinn J. (2024). National Greenhouse
Gas Emissions Reduction Potential from Adopting Anaerobic Digestion
on Large-Scale Dairy Farms in the United States. Environ. Sci. Technol..

[39] Gavrilova, O. ; Leip, A. ; Dong, H. ; MacDonald, J. ; Bravo, C. ; Amon, B. ; Rosale, R. ; Prado, A. ; Lima, M. ; Oyhantcabal, W. ; van der Weerden, T. Chapter 10: Emissions from Livestock and Manure Management; IPCC, 2019.

[40] Leytem, A. ; Archibeque, S. ; Cole, N. ; Gunter, S. ; Hristov, A. ; Johnson, K. ; Kebreab, E. ; Kohn, R. ; Liao, W. ; Toureene, C. , ; Chapter 4: Quantifying greenhouse gas sources and sinks in animal production systems. In Quantifying greenhouse gas fluxes in agriculture and forestry: methods for entity-scale inventory; USDA, 2024.

[41] US EPA. Title 40, Chapter 1, Subchapter C, Part 98, Subpart JJ- Manure Management; US EPA, 2024.

[42] Hergoualc’h, K. ; Akiyama, H. ; Bernoux, M. ; Chirinda, N. ; Prado, A. ; Kasimir, A. ; MacDonald, J. ; Ogle, S. ; Regina, K. ; van der Weerden, T. Chapter 11: N2O emissions from managed soils, and CO2 emissions from lime and urea application. 2019 Refinement To The 2006 IPCC Guidelines For National Greenhouse Gas Inventories: volume 4 - Agriculture, Forestry, And Other Land Use, Intergovernmental Panel On Climate Change; IPCC, 2019.

[43] Shine P., Upton J., Sefeedpari P., Murphy M. (2020). Energy Consumption
on Dairy Farms: A Review of Monitoring, Prediction Modelling, and
Analyses. Energies.

[44] EPA Emissions & Generation Resource Integrated Database. 2024, https://www.epa.gov/egrid. [Accessed 3 January 2025].

[45] Argonne National Laboratory, R&D GREET Model, 2024, https://greet.anl.gov. [Accessed 3 January 2025].

[46] Collet P., Flottes E., Favre A., Raynal L., Pierre H., Capela S., Peregrina C. (2017). Techno-economic
and life cycle assessment
of methane production via biogas upgrading and power to gas technology. Appl. Energy.

[47] US EPA. Catalogue of CHP Technologies; US EPA, 2017.

[48] Le Fevre, C. A review of prospects for natural gas as a fuel in road transport; The Oxford Institute for Energy Studies, 2019.

[49] Kytta V., Roitto M., Astaptsev A., Saarinen M., Tuomisto H. (2022). Review and
expert survey of allocatin methods used in life cycle assessment of
milk and beef. Int. J. Life Cycle Assess..

[50] Ineichen S., Schenker U., Nemecek T., Reidy B. (2022). Allocation of environmental
burdens in dairy systems: Expanding a biophysical approach for application
to larger meat-to-milk ratios. Livestock Science.

[51] World Resources Institute. GHG Protocol Product Life Cycle Accounting and Reporting Standard; World Resources Institute, 2011.

[52] Heijungs, R. Probability, Statistics, and Life Cycle Assessment: guidance for dealing with uncertainty and sensitivity; Springer Nature: Switzerland, 2024.

[53] US EPA. Inventory of U.S. Greenhouse Gas Emissions and Sinks: 1990–2020: Annex 6; United States Environmental Protection Agency. 2022.

[54] Hristov A. N., Kebreab E., Niu M., Oh J., Melgar A., Bannink A., Bayat A. R., Boland T., Brito A. F., Casper D. (2018). Uncertainties in enteric
methane inventories, measurement
techniques, and prediction models. J. Dairy
Sci..

[55] Basset-Mens C., Kelliher F., Ledgard S., Cox N. (2009). Uncertainty of global
warming potential for milk production on a New Zealand farm and implications
for decision making. Int. J. Life Cycle Assess..

[56] Wernet G., Bauer C., Steubing B., Reinhard J., Moreno-Ruiz E., Weidema B. (2016). The ecoinvent database
version 3 (part 1): overview
and methodology. Int. J. Life Cycle Assess..

[57] Mazzetto A., Falconer S., Ledgard S. (2022). Mapping the Carbon footprint of milk
production from cattle: A systematic review. J. Dairy Sci..

[58] Gerber, P. ; Vellinga, T. ; Dietze, K. ; Opio, C. Greenhouse gas emissions from the dairy sector - a life cycle assessment; Food and Agriculture Organization: Rome, Italy, 2010.

[59] Rotz C., Beegle D., Bernard J., Leytem A., Feyereisen G., Hagevoort R., Harrison J., Aksland G., Thoma G. (2024). Fifty year
of environmental progress for United States dairy farms. J. Dairy Sci..

[60] Rotz, C. ; Thoma, G. Assessing the carbon footprint of dairy production systems. In Large Dairy Herd Management; American Dairy Science Association, 2017, Vol. 3, pp. 19–31.

[61] Capper J., Cady R., Bauman D. (2009). The environmental
impact of dairy
production: 1944 compared with 2007. J. Animal
Sci..

[63] NCEI, County MappingNational Centers for Environmental Information. 2024. https://www.ncei.noaa.gov/access/monitoring/climate-at-a-glance/county/mapping/110/tavg/200712/12/value. [Accessed 3 January 2025].

