# Correction to “Spatially Resolved Greenhouse Gas Emissions of U.S. Milk Production in 2020”

**DOI:** 10.1021/acs.est.5c12673

**Published:** 2025-10-21

**Authors:** Rylie Pelton, Juan Tricarico, Fabian Bernal, Mary Beth de Ondarza, Tim Kurt

Several values in the results and figures have been corrected. Revised data are supplied in Figures 3–7 and the corrected Supporting Information (Tables S1, S6, and S9–S17 and Sections S6 and S7). These amendments adjust the absolute greenhouse gas emission totals and emission intensity metrics for individual regions and for the national aggregate but do not alter the qualitative conclusions of the study.

- *Milk Protein Specification*. Protein values in Table S1 represented true protein. Because the fat and protein corrected milk (FPCM) metric is standardized to 3.3% crude protein, the values have been converted using CP (%) = TP (%) + 0.19%, which accounts for nonprotein nitrogen in dairy milk. This conversion revises the FPCM total for 2007 and 2020 in Table S6.
- *Replacement Rate Data Source*. Replacement rates have been updated to use USDA National Agricultural Statistics Service (NASS) annual series, replacing Capper and Cady’s 2020 point-in-time averages from the Dairy Metrics Database, to provide a consistent, reliable basis for year-over-year national tracking of emissions. Revised rates and corresponding total lifetimes before cull estimates are reported in Tables S2 and S5.
- *Table Title Clarifications*. The titles of Tables S7, S8, and S10–S16 have been revised for the sake of clarity.
- *Regional Byproduct Feed Distribution*. The West, South, and Northeast distributions in Table S9 were inadvertently interchanged; the correct regional assignments have now been restored.
- *Byproduct Feed Percentage Basis*. Percentages in Table S9 were compiled on an as-fed basis but were interpreted in the LCA model as dry matter values. The distributions have been recalculated on a dry matter basis, and the model inputs updated.
- *Feed Nutrient Composition*. Dietary nutrient composition values in Tables S10–S16 have been aligned with those generated by the feed-formulation software used to construct the diets, resolving a minor inconsistency. Previous estimates drew on generalized French feed tables and, in places, referenced % organic matter digestibility rather than % organic matter; the updated tables now reflect the specific feed mixes (e.g., alternative grass types) and the correct OM metric.
- *Allocation Basis for Whey Emission Factors*. The emission factors for whey feed inputs in Table S17 were previously based on a milk-solids allocation basis, which is appropriate for whey destined for human consumption. However, consistent with International Dairy Federation guidance for coproducts destined for animal feed, the emissions have been recalculated on the basis of economic allocation.

Corrected excerpts from the Results and Discussion are provided below. All other regional inputs, model parameters, and assessment boundaries remain unchanged from the original publication.

## Results and Discussion

### Absolute Emissions and Contribution by Source

Total U.S. cradle-to-farm-gate GHG emissions attributed to milk production in 2020 are estimated to be 131.97 million metric tonnes (MMT) of CO_2_e on average, corresponding to 30.03 MMT attributed to the beef system. Concentrate feed production (grains and byproducts) contributes an almost equivalent share of emissions to enteric fermentation, at 36.8 MMT CO_2_e, or *28%* of total emissions, with regional variations from *24%* to *41%* (Figures 3 and 4). This includes emissions from multiple feed sources influenced by regional production practices, environmental conditions, and sourcing. The large contribution from concentrate feeds in part reflects the inclusion of LUC estimates for 68 feed products, increasing emissions beyond prior estimates. Feed-related emissions, including concentrate, forage, milling/mixing, and transport, account for about *1.5 times* the emissions of enteric fermentation but arise from multiple fragmented sources along the supply chain, making management more complex and necessitating significant coordination across multiple regions, crops of production, and various points along the supply chain. In contrast, enteric fermentation contributes about 37.9 MMT CO_2_e, or *29%* of total emissions (*24–32%* range across regions), representing the largest single source of emissions. This emphasizes the importance of targeting methane emissions from digestive processes to meet net GHG neutrality targets.

**Figure 3:**
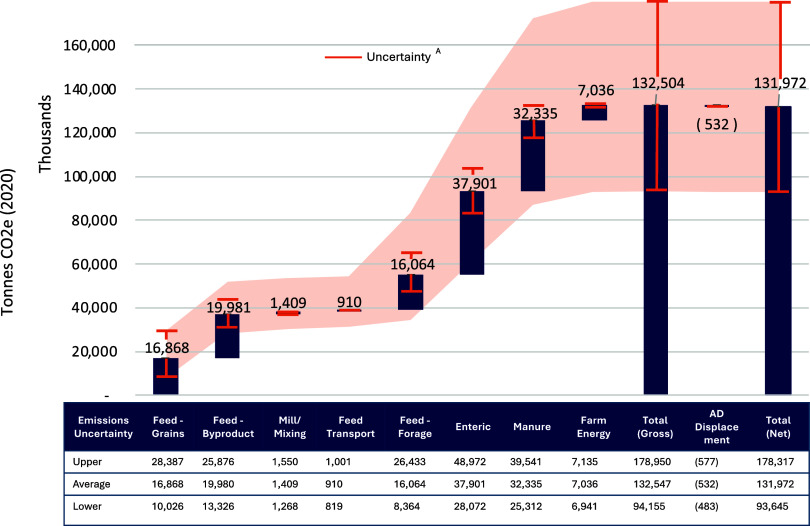
Total cradle-to-farm gate greenhouse gas emissions from milk production across emission sources and uncertainty ranges. ^A^Considers standard error uncertainty in enteric methane formation, VS and N excretion models, and standard deviation estimates for feed emission factors and dry matter intake.

**Figure 4:**
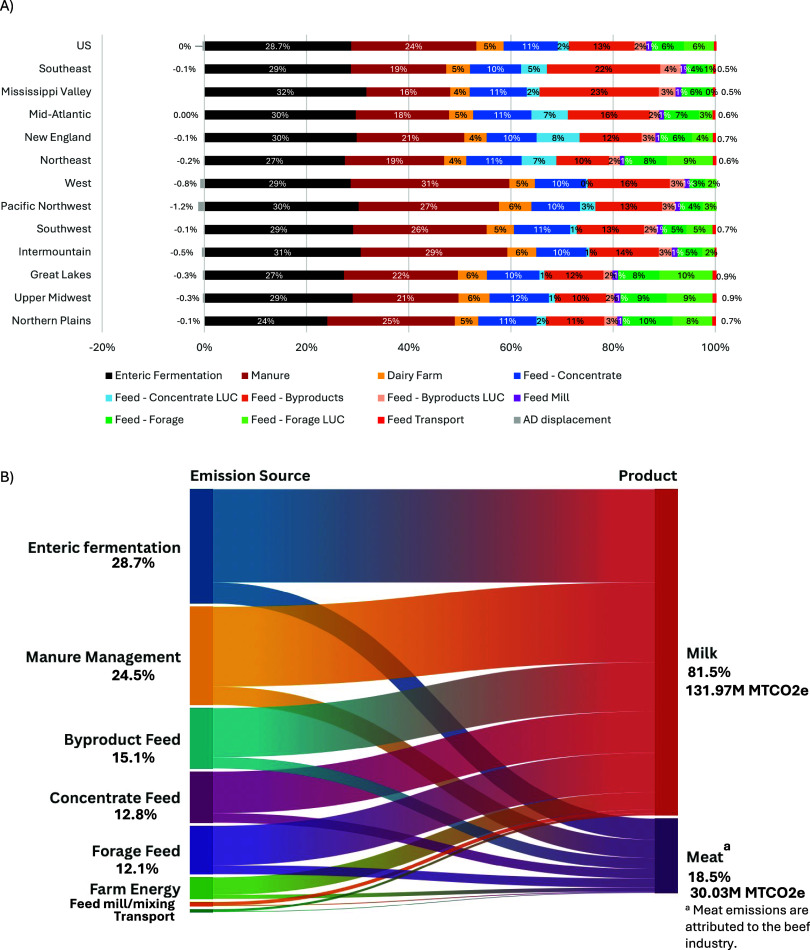
(A) Regional contribution of emission sources (see Figure S4 for uncertainty) and (B) contribution of cradle-to-farm-gate emissions across sources and products.

While LUC had previously been excluded from analyses, as it was assumed not to be a major contributor within the United States, the 2022 International Dairy Federation (IDF) guidelines,^10^ along with other key protocols such as the GHG Protocol Product Standard^51^ and FAO LEAP guidance,^22^ now include LUC as a critical component in emissions reporting and assessments. This reflects the increased recognition of LUC’s contribution to total emissions and the necessity for comprehensive accounting in environmental analyses. This study underscores its substantial contribution in the context of feed crops used by the dairy industry, averaging *10%* of total dairy emissions with regional ranges of *6–18%*.

Manure emissions, from both CH_4_ and N_2_O, total approximately 32.3 MMT CO_2_e, or *25%* of total emissions (*16–31%* across regions). Methane represents about *78%* of manure emissions and ranges between *56%* and *84%* of total emission across regions due to differences in management systems, with *22%* from N_2_O on average. Regions with higher use of pasture/range/paddock (PRP) systems, like the Mississippi Valley, have higher than average N_2_O emissions, while areas with more anaerobic lagoons, like the West, have manure emissions driven mostly by CH_4_. Anaerobic digesters with biogas capture and utilization provide emissions offset credits, averaging *0.4%* across the industry and reaching up to *1.2%* in high-adoption areas. Emissions from forage feeds, farm energy, feed mills/mixing, and feed transport represent 12%, 5%, 1%, and 0.7%, respectively.

In 2020, U.S. cradle-to-farm-gate GHG emissions from the dairy sector were largely driven by biogenic methane, contributing approximately *48%* of the net total emissions (Figure 5 and Table S29). Emissions from feed inputs (i.e., “aggregate CO_2_e”) contribute around a quarter of total emissions but could not be broken down by specific GHGs due to data limitations; these likely include primarily CO_2_ from manufacturing inputs (e.g., fertilizers and pesticides), fuel combustion, lime applications and LUC, and N_2_O from field-applied fertilizers. The distribution between CO_2_ and N_2_O likely differs across byproduct feeds. For example, for DDGS feed, CO_2_ represents around 93% of total emissions and N_2_O represents 1%, whereas for soybean meals, CO_2_ represents 50% of total emissions and N_2_O represents 42%.^25,56^ N_2_O (from manure and field-applied fertilizers) and LUC account for 11% and *10%*, respectively, of total dairy emissions, while fossil fuel CO_2_ from crop and dairy farm operations contributes around 6%. Feed practices that increase soil carbon storage, such as transitioning from intensive tillage to no tillage practices and addition of cover crops, lead to a baseline reduction of net emissions by approximately 1.8 MMT CO_2_e, or *1.3%* of the total emissions.

**Figure 5:**
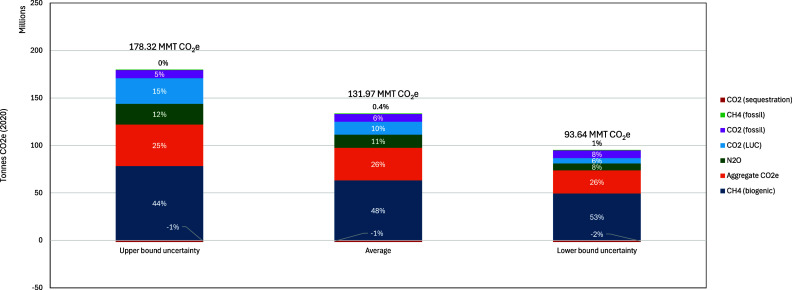
Contribution of greenhouse gases to 2020 U.S. dairy emissions (in CO_2_e), which considers standard error uncertainty in enteric methane formation, VS and N excretion models, and standard deviation estimates for feed emission factors and dry matter intake. Note that aggregate CO_2_e cannot be disaggregated due to data limitations; however, because these emissions are from feed inputs, GHGs are likely to be split mostly between CO_2_ and N_2_O categories.

### Emission Intensity and Benchmarking Comparisons

The average U.S. emissions intensity allocated to milk is estimated at 1.29 kg of CO_2_e/kg of FPCM, with regional variations from 1.17 and 1.88 kg of CO_2_e/kg of FPCM (Table S30). These differences stem from factors such as feed diets, sourcing regions, manure management, and milk production rates, where lower milk yields increase emissions intensity per kilogram of FPCM. Notably, the Upper Midwest and Western regions, which produce the largest quantities of milk across the United States, have emission intensities near or slightly below the national average, while regions that produce smaller quantities of milk are consistent with higher intensities. Some of these regions also exhibit higher prevalence of pasture/range/paddock grazing systems, however, which may be desired by certain dairy buyers.

The emissions range estimated in this study aligns with previous assessments, which reported values from 0.69 to 1.84 kg of CO_2_e/kg of FPCM.^1,4–6,57–61^ Although direct comparisons are complex due to differences in assumptions, data sources, model structures, and changes in industry practices over time, we isolated key components for comparative analysis. Figure 6A presents our sensitivity analysis, exploring the effects of alternative models for estimating VS, N excretion, and enteric fermentation. Using these alternative models, total 2020 emissions for U.S. dairy range from 117.8 to 150.2 MMT CO_2_e, with an average emission intensity of 1.16–1.47 kg of CO_2_e/kg of FPCM, depending on the models used. Detailed comparisons between models are available in Section S7 (Tables S32 and S33 and Figure 2). These results highlight how model choice impacts emissions estimates, underscoring the importance of using models that reflect the latest advancements in emission estimation and the potential limitations of harmonization when relying on outdated or less accurate models.

**Figure 6:**
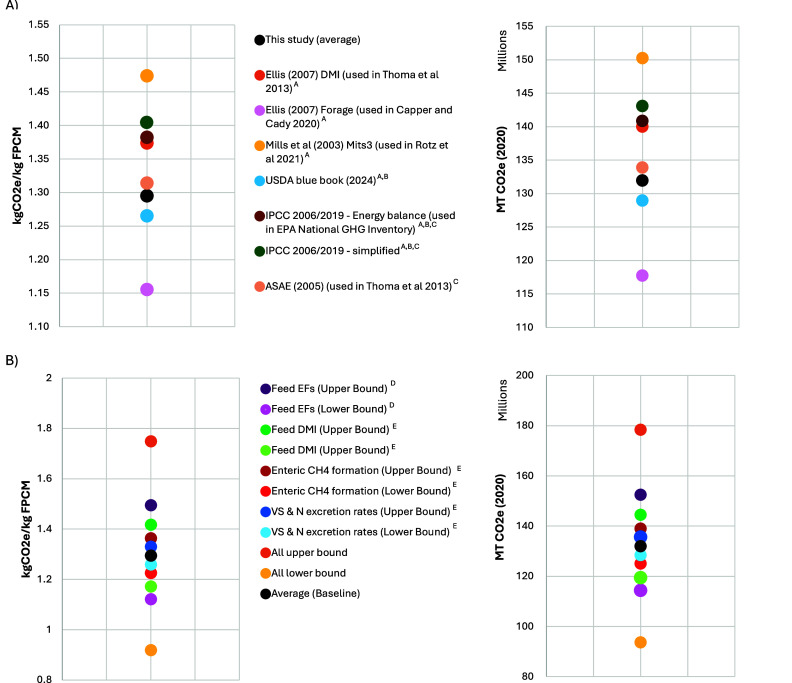
Sensitivity analysis of 2020 GHG emissions intensity and absolute GHG emissions considering (A) alternative models for estimating enteric methane and manure VS and N excretion rates and associated emissions and B) uncertainty (characterized by standard error and deviation) in key parameters, including feed emission factors, feed dry matter intake, enteric methane formation, and manure volatile solids and N excretion rates. ^A^Based on alternative enteric fermentation models. ^B^Based on alternative enteric fermentation and manure VS and N models. ^C^Based on alternative manure N models. ^D^Based on the standard deviation of regional emission factors across feed types. ^E^Based on the standard error in models.

In addition to sensitivity in model selection, our analysis includes an estimate of uncertainty within feed, enteric methane, and manure emissions sources, with uncertainty bounds determined based on the standard errors of prediction models (e.g., enteric methane, VS, and N excretion) and the standard deviation across regional data inputs (e.g., feed emission factors). Figures 3 and 6B indicate that our national estimates range from 93.6 to 178.3 MMT CO_2_e, or from 0.92 to 1.75 kg of CO_2_e/kg of FPCM, when accounting for all evaluated sources of uncertainty (see Figure S3 for regional ranges). Figure 6B and Tables S34 and S35 present uncertainty ranges for each emission source, showing the uncertainty in the selected manure VS and N excretion models has a weaker effect on the range in total emissions than the uncertainty in the selected enteric methane model. A ±10% uncertainty in dry matter intake (DMI) results in a corresponding 10% change in total emissions, as DMI directly scales emissions from feed inputs, enteric fermentation, and manure. Emission estimates are most sensitive to uncertainty in feed emission factors, highlighting the importance of detailed data to capture the spatially heterogeneous impacts of feed production combined with the need for enhanced supply chain transparency to identify relevant sourcing regions.

Using recent models and spatial data offers a more precise view of current dairy practices and associated emissions, though uncertainty remains. Enteric fermentation and manure continue to be significant, averaging *53.2%* of total farm-gate emissions, though lower than previous estimates (see Figure S4 for ranges across regions and uncertainty). A key difference in this study is our inclusion of LUC emissions, historically assumed to be minimal in the United States. Our findings, however, show LUC as a notable contributor, and addressing it is necessary to meet climate action targets. While the dairy industry drives demand for feed crops, among other industries, it accounts for only 9%, 1%, and 5% of total land conversion for corn, soy, and wheat, respectively.^19^

Table S36 examines the effects of excluding LUC or biogas displacement credits and including N_2_O emissions from silage storage. Table S37 further assesses how emissions vary under different GWP factors from IPCC assessment reports (AR4 and AR5 with and without climate carbon feedback, respectively, and AR6). The analysis reveals notable variations in biogenic CH_4_ emissions; under AR6, biogenic CH_4_ contributes 63.1 MMT of CO_2_e (*48%* of total emissions) while AR5 with climate carbon feedback increases this to 78.9 MMT of CO_2_e (53%). This sensitivity to characterization factors highlights the importance of selecting appropriate factors when benchmarking emissions. Across the four GWP characterization methods, total emissions range from *127.5* MMT of CO_2_e (AR4) to *148.6* MMT of CO_2_e (AR5 with climate carbon feedback), illustrating the significant impact that methodological choices can have on emission estimates.

Figure 7 shows our final analysis comparing the 2020 emissions with updated estimates for 2007. This comparison revisits the 2007 data using the latest models, system boundaries, and consistent assumptions, offering a more reliable basis for assessing changes over time rather than directly comparing with previous assessments.^2^ The analysis indicates that emission intensity has decreased from an average of 1.50 kg of CO_2_e/kg of FPCM in 2007 to 1.29 kg of CO_2_e/kg of FPCM in 2020, reflecting improved efficiency and emissions management. However, despite this reduction in intensity, total emissions have increased from 120 million tonnes of CO_2_e in 2007 to 132 million tonnes in 2020, driven by a substantial increase in total milk production from 81 to 102 million tonnes of FPCM. This demonstrates progress in emission reductions per unit output but also underscores challenges posed by increased production for achieving GHG neutrality targets. The updated comparison to the 2007 baseline offers a consistent and refined view, providing valuable insights that cannot be captured through direct comparisons with earlier studies, due to differences in their methodologies and assumptions.

**Figure 7:**
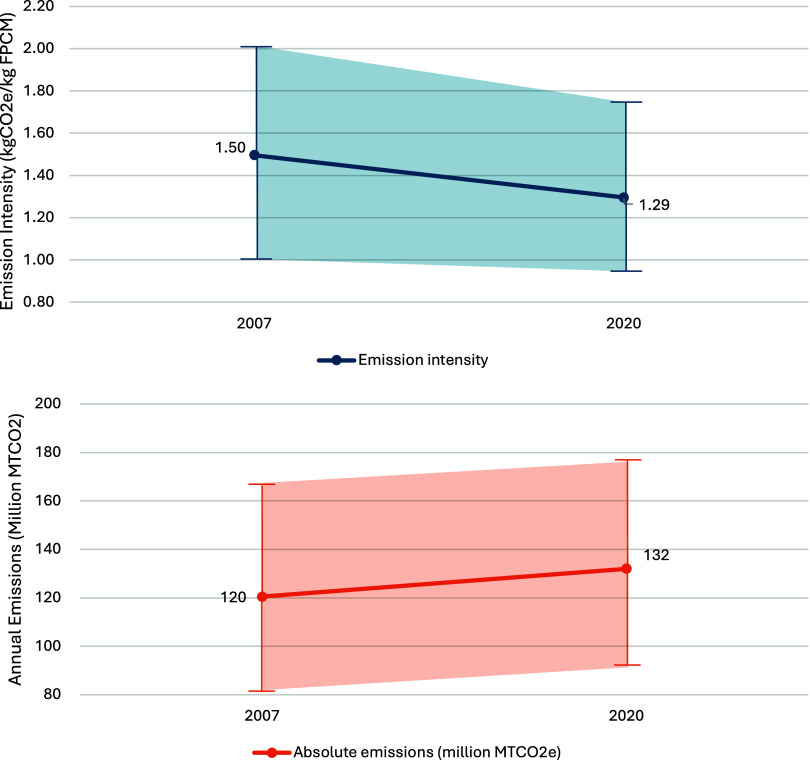
Comparison of the emission intensity and annual total emissions in 2020 (with 102 million tonnes of FPCM) to 2007 (with 81 million tonnes of FPCM), with uncertainty bounds (see Table S38), considering the standard error uncertainty in enteric methane formation, VS and N excretion models, and standard deviation estimates for feed emission factors and dry matter intake.

## Supplementary Material



## Notes

Includes additional details and assumptions related to growth and performance, feed, enteric fermentation, manure management, and farm energy, and additional information about uncertainty and sensitivity analyses, benchmark comparisons, and data quality (PDF)

