# Supplementary material for: Spatially Resolved Greenhouse Gas Emissions of U.S. Milk Production in 2020
Source: Environ Sci Technol. 2025 May 7;59(19):9552–64. doi: 10.1021/acs.est.5c01166 (PMC12096441; doi:10.1021/acs.est.5c01166)
Supplement: Supplementary file 1 [file es5c01166_si_001.pdf]

Supplemental Information for:

Spatially Resolved Greenhouse Gas Emissions of U.S. Milk Production in 2020

Rylie Pelton[1,2], Juan Tricarico[3], Fabian Bernal[3], Mary Beth de Ondarza[4], Tim Kurt[3]

Affiliations:
[1]LEIF, LLC, St. Paul, MN 55113, USA
[2] University of Minnesota, Institute on the Environment, St. Paul, MN 55108, USA
[3] Dairy Management Inc., Rosemount, IL, 60018, USA
[4] Paradox Nutrition, LLC, Plattsburgh, NY, 12901, USA

Summary: 43 pages, 39 tables, 4 figures, 30 equations.

Table of Contents

Section S1. Growth and Performance 2
Section S2: Feed 5
Section S3. Enteric Fermentation 17
Section S4. Manure 17
Section S5. Farm Energy 24
Section S6. Results 26
Section S7. Sensitivity & Uncertainty Analysis 27
Section S8. Data quality 40

Section S1. Growth and Performance

Table S1. Lactation rates, milk fat and protein contents, and total annual FPCM produced in 2020 and 2007 (as indicated in brackets [ ]) by region.

Region	Kg milk/cow/day	Kg FPCM/cow/day	Milk protein %	Milk fat %	Total tonnes FPCM
Northern Plains	29.0 [22.2]	29.7 [21.3]	**3.2% [3.1%]**	**4.2% [3.7%]**	**2.3M [1.4M]**
Upper Midwest	29.7 [23.5]	29.8 [22.6]	**3.2% [3.0%]**	**4.1% [3.7%]**	**21.9M [16.7M]**
Great Lakes	30.9 [25.0]	30.3 [23.8]	**3.2% [3.1%]**	**3.9% [3.7%]**	**9.6M [6.8M]**
Intermountain	31.2 [27.5]	30.8 [26.0]	**3.2% [3.1%]**	**3.9% [3.6%]**	**10.8M [7.0M]**
Southwest	30.4 [25.8]	30.3 [24.6]	**3.3% [3.1%]**	**4.0% [3.7%]**	**14.8M [9.7M]**
Pacific Northwest	28.9 [27.0]	29.3 [25.9]	**3.3% [3.1%]**	**4.1% [3.7%]**	**4.3M [3.3M]**
West	29.8 [27.1]	29.5 [25.8]	**3.2% [3.1%]**	**4.0% [3.7%]**	**18.9M [17.3M]**
Northeast	28.7 [23.7]	28.1 [22.7]	**3.2% [3.1%]**	**3.9% [3.7%]**	**11.4M [9.9M]**
New England	26.7 [22.9]	26.6 [22.1]	**3.2% [3.1%]**	**4.1% [3.8%]**	**1.8M [1.8M]**
Mid-Atlantic	24.5 [20.1]	24.5 [19.3]	**3.2% [3.1%]**	**3.9% [3.7%]**	**2.3M [2.8M]**
Mississippi Valley	17.8 [18.6]	17.5 [17.7]	**3.2% [3.1%]**	**3.9% [3.6%]**	**0.7M [1.2M]**
Southeast	26.0 [21.3]	24.7 [20.2]	**3.2% [3.1%]**	**3.7% [3.6%]**	**1.8M [1.5M]**

Table S2. Estimated time spent in each lifecycle stage in 2020 and 2007

Year	Days in Calving[A]	Days spent as Heifer Replacement (15 months)[A]	Days spent as Heifer Replacement (24 months)[A]	Total days before first calving[B]	Days as Dry Cow[B,C]	Days in Lactation[B]	Total Lifetime Days/Cow
2020	255	199	235	771	155	968	1893
2007	255	201	244	780	178	1087	2045

[A] Based on daily average gain (kg/day) for each phase of the lifecycle (0.76 kg/day for calves, 0.82 for heifers) and typical animal mass (see Supplemental Table 4) associated with each life phase (e.g., [weight of heifer at 24 mo - weight of heifer at 15 mo] / [weight gain/day]).

[B] Average age at first calving, calving intervals, dry period length, and lifetime lactations based on Dairy Metrics Database in Capper and cady (2019) [1], see Supplemental Table 5

[C] Total time spent in dry period over lifetime based on average dry period and average number of lifetime lactations from Dairy Metrics Database in Capper and Cady (2019) [1], see Supplemental Table 5.

Table S3. Herd population size across regions (thousands of dairy cows) [2]

Region	2020	2007
Northern Plains	209	173
Upper Midwest	2008	2023
Great Lakes	867	776
Intermountain	958	741
Southwest	1337	1081
Pacific Northwest	406	353
West	1755	1840
Northeast	1113	1187
New England	190	223
Mid-Atlantic	256	397
Mississippi Valley	102	192
Southeast	194	202

Table S4. Typical liveweights (kg) by growth phase and cattle category.

Cattle Category	Liveweight kg
Dairy Cows	680
Heifer Replacements (15 months)	397
Heifer Replacements (24 months)	590
Weaned Calf	234
Calf birthweight	40
Bulls	959

Table S5. Growth and performance parameters based on the Dairy Metrics Database [1]

Key parameters	2020	2007
Dry period per lactation cycle (days)	57	60
Calving Interval (days)	414	426
Number of lifetime lactation	2.71	2.97
Cull rate (%)	36.9%	33.7%
Average age at first calving (months)	25.7	26.0
Cow/bull ratio	25	25
Number breeding seasons for bulls	4	4
Dairy cow mortality	5.2%	5.3%
Heifer (weaning to breeding) mortality	1.9%	1.9%
Calf (live birth to wean) mortality	6.8%	6.8%

Equation S1. Estimation of the total lifetime net energy for milk production (based on NASEM 2021) [3].

$$NE_{milk} = (9.29 \times MF + 5.5 \times CP + 0.192) \times 4.184$$

Where NE_L is the net energy for milk production per kg of FPCM (MJ/kg FPCM), MF is the concentration of fat in FPCM milk (4 kg fat/kg milk), CP is the concentration of crude protein in FPCM milk (3.3 kg crude protein/kg milk).

Equation S2. Estimation of the total lifetime net energy for growth (based on IPCC 2019) [4].

$$NE_{G,P} = 22.02 \times \left(\frac{ABW_P - PBW_{P-1}}{C \times MW_P}\right)^{0.75} \times ADG_P^{1.097} \times D_P$$

Where $NE_{G,P}$ is the net energy required for growth (MJ) in each growth phase over the lifetime , ABW_P is the average live body weight of the animals in each growth phase (kg), PBW is the target body weight of the previous growth phase, MW_P is the mature body weight of the animal in the current growth phase, C is a constant with a value of 0.8 for heifers and 1.2 for bulls, ADG_P is the average daily weight gain for each growth phase (kg/day), and D_P is the total days of growth in each growth phase. For cows (lactating & dry), only the number of days required to grow to target weight is considered.

Equation S3. Estimation of the net energy required for gestational pregnancy (based on IPCC 2019) [4].

$$NE_{Preg} = \frac{0.1 \times 0.386 \times {BW_{cow}}^{0.75} \times G}{BW_{calf}}$$

Where NE_{preg} is the net energy (MJ) required for pregnancy over the lifetime of the cow, BW_{cow} is the animal mass of the cow (kg), G is the total gestational days (assumed to be 280), and BW_{calf} is the body weight of the calf at birth (kg).

Equation S4. Estimation of the allocation factor for attributing impacts to milk outputs (IDF 2022) [5].

$$AF_{milk} = \frac{NE_{milk} \times milk}{NE_{milk} \times milk + NE_{Preg} \times meat_{calves} \frac{\sum_P NE_{G,P}}{BW_{cow}} \times meat_{cull}}$$

Where AF_{milk} is the allocation factor for milk, NE_{milk} is the net energy required to produce milk (MJ/kg FPCM), milk is the total annual fat and protein corrected milk produced (kg FPCM), NE_{Preg} is the net energy for pregnancy to grow the calves, $Meat_{calves}$ is the total annual liveweight

(kg) of calves sold to the beef system for finishing, $NE_{G,P}$ is the lifetime net energy for growth in each growth phase associated with producing a cow (MJ/kg of meat), including the lifetime net energy of producing bulls allocated to each cow serviced over lifetime, and $Meat_{cull}$ is the total annual liveweight of culled cows.

Table S6. Total annual dairy meat production in 2020 and 2007 [6].

Category	2020	2007
Total commercial slaughter head	32,785,700	34,264,000
Percent Steers	49.3%	51.3%
Percent Heifers	29.4%	30.3%
Estimated % Dairy Steers	19.9%	21%
Dairy Cows	9.5%	7.4%
Total Culled Dairy Cows (tonnes liveweight)	2,119,170	1,610,141
Total Dairy Calves sold to feedlots (tonnes liveweight)	1,524,091	1,680,857
Tonnes FPCM	101,249,111	79,347,504
Milk allocation factor	81%	78%

Section S2: Feed

Lactating Diets

Regional lactating diets were modeled using the NDS Professional tool and the CNCPS 6.55 framework [7]. Dry matter intake (DMI) predictions used NRC (2001) (equation 1-2) and NASEM (2021) (equations 2-2) for 2007 and 2020 diets, respectively [8, 3]. Nutrient profiles for main feedstuffs were provided by the NDS model feed dictionary, while byproduct blends were taken from de Ondarza and Tricarico (2021) with the Northeast analysis used for the Mid-Atlantic, Northeast, and New England regions, the Midwest analysis used for the Great Lakes, Northern Plains, and Upper Midwest regions, the South analysis used for the Mississippi Valley and Southeast regions, and the West analysis used for the Intermountain, Pacific Northwest, Southwest, and West regions [9].

Lactating diet ingredients (%DM) (corn silage, alfalfa, grass, corn grain, byproduct feeds) for each region were based on ration information submitted between April, 2017 and June, 2022 from 2702 dairy farms which voluntarily participated in evaluations conducted by the Farmers Assuring Responsible Management environmental stewardship program [10]. Regional diet ingredient means were weighted based on the number of lactating cows per reporting dairy farm. The FARM survey represented lactating dairy diets from 40 U.S. states and 2,622,434 cows [10]. Due to limited survey data from the Mississippi Valley and Southeast regions, diet information was combined to provide the same lactating diet ingredient profile for both regions.

Diets were designed to provide metabolizable energy (ME) and metabolizable protein (MP) necessary to support mean regional production of milk and milk components in 2007 and in 2020

based on USDA data reported by state [11, 12, 13, 14]. True protein data was only available for seven Federal Milk Marketing Orders [15]. In this case, 2020 USDA data [13] on the population of FMMO's was used to calculate a weighted average for state true protein values. If true protein data was unavailable for a particular state, U.S. averages of 3.06% for 2007 and 3.21% for 2021 were used.

Diet nutrient analyses including ME predicted milk, ME (Mcal/d), ME (% requirement), MP predicted milk, MP (g/d), and MP (% requirement), NASEM Milk TP, kg, urine N, and wet feces N were calculated using the NDS Professional model based on the CNCPS 6.55 model [7]. NASEM calculated milk (kg) equaled NASEM Milk true protein (kg) divided by milk true protein (%). For 2007 diets, ME requirements were calculated using NRC (2001) equations (ME for maintenance (0.08* BW (body weight)$^{0.75}$) and ME for production (Milk kg *((0.0929* milk fat%) + (0.0563*milk true protein %) + (0.192)) (Equation 2-16) [16]. For 2020 diets, ME requirements were calculated using NASEM (2021) equations (ME for maintenance (0.10* BW$^{0.75}$) (Equation 3-13) and ME for production (Milk kg *((9.29 * (kg milkfat / kg milk)) + (5.85*(kg milk true protein /kg milk)) + (3.95*0.0485)) (Equation 3-14b) [3].

Dry Cow Diets

Regional dry cow diets were developed using the NDS Professional model (RUM&N Sas, Italy) based on the CNCPS 6.55 model [7]. Dry matter intakes were predicted for each region based on Table 14-9 from NRC (2001) for 2007 diets and based on Table 21-1 from NASEM (2021) for 2020 diets as a weighted average of the far-off (60-21 days before calving) (65%) and the pre-fresh period (21-0 days before calving (35%) [3]. Nutrient analyses for corn silage, alfalfa, grass, and corn grain were provided by the NDS Professional model feed dictionary. Nutrient analyses for regional feed byproduct blends were obtained from de Ondarza and Tricarico (2021) with the Northeast analysis used for the Mid-Atlantic, Northeast, and New England regions, the Midwest analysis used for the Great Lakes, Northern Plains, and Upper Midwest regions, the South analysis used for the Mississippi Valley and Southeast regions, and the West analysis used for the Intermountain, Pacific Northwest, Southwest, and West regions [9].

Dry cow diet ingredients (%DM) (corn silage, alfalfa, grass, corn grain, byproduct feeds) for each region were based Table 2 of Asselin-Balencon et al. (2013) using Region 1 for the Northeast and New England, Region 2 for the Mid-Atlantic, Southeast, and Mississippi Valley, Region 3 for the Upper Midwest and Great Lakes, Region 4 for the Northern Plains, Intermountain, and Southwest, and Region 5 for the Pacific Northwest and West [17].
Diet nutrient analyses including ME (Mcal/d), NEl (Net Energy for Lactation) (Mcal/d), MP (g/d), urine N, and wet feces N were calculated using the NDS Professional model based on the CNCPS 6.55 model [7]. Diets were designed to provide metabolizable energy and protein necessary for dry cows based on Table 14-9 from NRC (2001) for 2007 diets [16] and based on Table 21-1 from NASEM (2021) for 2021 diets as a weighted average of the far-off (60-21 days before calving) (65%) and the pre-fresh period (21-0 days before calving (35%) [3].

Heifer Diets

Regional heifer diets were developed using the NDS Professional model based on the CNCPS 6.55 model [7]. Dry matter intakes were predicted for each region for a 350 kg heifer gaining 0.8 kg/d based on Table 14-13 from NRC (2001) for 2007 diets and based on Table 21-1 from NASEM (2021) for 2020 diets [16]. Nutrient analyses for corn silage, alfalfa, grass, and corn

grain were provided by the NDS Professional model feed dictionary. Nutrient analyses for regional feed byproduct blends were obtained from de Ondarza and Tricarico (2021) with the Northeast analysis used for the Mid-Atlantic, Northeast, and New England regions, the Midwest analysis used for the Great Lakes, Northern Plains, and Upper Midwest regions, the South analysis used for the Mississippi Valley and Southeast regions, and the West analysis used for the Intermountain, Pacific Northwest, Southwest, and West regions [9].

Dry cow diet ingredients (%DM) (corn silage, alfalfa, grass, corn grain, byproduct feeds) for each region were based Table 3 of Asselin-Balencon et al. (2013) using Region 1 for the Northeast and New England, Region 2 for the Mid-Atlantic, Southeast, and Mississippi Valley, Region 3 for the Upper Midwest and Great Lakes, Region 4 for the Northern Plains, Intermountain, and Southwest, and Region 5 for the Pacific Northwest and West [17].
Diet nutrient analyses including ME (Mcal/d), MP (g/d), urine N, and wet feces N were calculated using the NDS Professional model based on the CNCPS 6.55 model [7]. Diets were designed to provide metabolizable energy and protein necessary for a 350 kg heifer gaining 0.8 kg/d based on Table 14-13 from NRC (2001) for 2007 diets [16] and based on Table 21-1 from NASEM (2021) for 2021 diets [3].

Table S7. Diet rations across dairy production regions and cattle categories in 2020.

Cattle Category	Diet Rations	Regions											
		**NP**	**UM**	**GL**	**IM**	**SW**	**PNW**	**West**	**NE**	**NEG**	**MA**	**MV**	**SE**
Lactating Dairy Cows	Corn silage [A]	37%	37%	37%	27%	23%	26%	24%	39%	40%	40%	31%	31%
	Alfalfa [A]	13%	18%	13%	21%	18%	18%	11%	14%	2%	6%	0%	0%
	Grass	1%	1%	2%	2%	6%	5%	6%	8%	20%	10%	9%	9%
	Corn Grain [A]	11%	15%	14%	17%	20%	19%	18%	14%	10%	15%	15%	15%
	Mineral Mix[C]	4%	3%	3%	3%	3%	4%	3%	3%	3%	4%	3%	3%
	Byproduct Blend	35%	25%	31%	30%	30%	29%	38%	22%	25%	26%	40%	40%
Dry Cows	Corn silage [A]	21%	34%	34%	21%	21%	17%	17%	41%	41%	10%	10%	10%
	Alfalfa [A]	18%	15%	15%	18%	18%	27%	27%	22%	22%	0%	0%	0%
	Grass	38%	28%	28%	38%	38%	32%	32%	22%	22%	63%	63%	63%
	Corn Grain [A]	3%	2%	2%	3%	3%	7%	7%	2%	2%	2%	2%	2%
	Mineral Mix[C]	2%	2%	2%	2%	2%	2%	2%	2%	2%	2%	2%	2%
	Byproduct Blend	19%	19%	19%	19%	19%	16%	16%	12%	12%	23%	23%	23%
Replacement Heifers	Corn silage [A]	17%	30%	30%	17%	17%	15%	15%	31%	31%	7%	23%	7%
	Alfalfa [A]	31%	26%	26%	31%	31%	19%	19%	36%	36%	4%	4%	4%
	Grass	26%	22%	22%	26%	26%	32%	32%	18%	18%	49%	51%	49%
	Corn Grain [A]	3%	4%	4%	3%	3%	5%	5%	3%	3%	8%	7%	8%
	Mineral Mix[C]	2%	3%	3%	2%	2%	3%	3%	3%	3%	3%	3%	3%
	Byproduct Blend	20%	15%	15%	20%	20%	25%	25%	10%	10%	30%	11%	30%
Bulls	Corn silage [A]	17%	30%	30%	17%	17%	15%	15%	31%	31%	23%	23%	23%
	Alfalfa [A]	31%	26%	26%	31%	31%	19%	19%	36%	36%	4%	4%	4%
	Grass	26%	22%	22%	26%	26%	32%	32%	18%	18%	51%	51%	51%
	Corn Grain [A]	3%	4%	4%	3%	3%	5%	5%	3%	3%	7%	7%	7%

	Mineral Mix[C]	2%	3%	3%	2%	2%	3%	3%	3%	3%	3%	3%	3%
	Byproduct Blend	20%	15%	15%	20%	20%	25%	25%	10%	10%	11%	11%	11%

Midwest byproduct blend = Northwest Plains (NP), Upper Midwest (UP), Great Lakes (GL); West byproduct blend = Intermountain (IM), Southwest (SW), Pacific Northwest (PNW), West; Northeast byproduct blend = Northeast (NE), New England (NEG), Mid-Atlantic (MA); South byproduct blend = Mississippi Valley (MV), Southeast (SE).
[A] Estimates based on FoodS^3 models considering county scale commodity flows and emission factors [18, 19]
[B] Estimated based on FAF5 commodity flows, supply and demand distributions; with spatially estimated emissions at state scale [20].
[C] Based on GFLI [21], literature, or other LCA databases reflecting national averages [22].

Table S8. Diet rations across dairy production regions and cattle categories in 2007.

Cattle Category	Diet Rations	Regions											
		**NP**	**UM**	**GL**	**IM**	**SW**	**PNW**	**West**	**NE**	**NEG**	**MA**	**MV**	**SE**
Lactating Dairy Cows	Corn silage [A]	37%	37%	37%	27%	23%	26%	24%	39%	40%	40%	31%	31%
	Alfalfa [A]	13%	18%	13%	21%	18%	18%	11%	14%	2%	6%	0%	0%
	Grass	1%	1%	2%	2%	6%	5%	6%	8%	20%	10%	9%	9%
	Corn Grain [A]	11%	15%	14%	17%	20%	19%	18%	14%	10%	15%	15%	15%
	Mineral Mix[C]	4%	3%	3%	3%	3%	3%	3%	3%	3%	4%	3%	3%
	Byproduct Blend	35%	25%	31%	30%	30%	29%	38%	22%	25%	26%	40%	41%
Dry Cows	Corn silage [A]	21%	34%	34%	21%	21%	17%	17%	41%	41%	10%	10%	10%
	Alfalfa [A]	18%	15%	15%	18%	18%	27%	27%	22%	22%	0%	0%	0%
	Grass	38%	28%	28%	38%	38%	32%	32%	22%	22%	63%	63%	63%
	Corn Grain [A]	3%	2%	2%	3%	3%	7%	7%	2%	2%	2%	2%	2%
	Mineral Mix[C]	2%	2%	2%	2%	2%	2%	2%	2%	2%	2%	2%	2%
	Byproduct Blend	19%	19%	19%	19%	19%	16%	16%	12%	12%	23%	23%	23%
Replacement Heifers	Corn silage [A]	17%	30%	30%	17%	17%	15%	15%	31%	31%	7%	7%	7%
	Alfalfa [A]	31%	26%	26%	31%	31%	19%	19%	36%	36%	4%	4%	4%
	Grass	26%	22%	22%	26%	26%	32%	32%	18%	18%	49%	49%	49%
	Corn Grain [A]	3%	4%	4%	3%	3%	5%	5%	3%	3%	8%	8%	8%
	Mineral Mix[C]	2%	3%	3%	2%	2%	3%	3%	3%	3%	3%	3%	3%
	Byproduct Blend	20%	15%	15%	20%	20%	25%	25%	10%	10%	30%	30%	30%
Bulls	Corn silage [A]	17%	30%	30%	17%	17%	15%	15%	31%	31%	23%	23%	23%
	Alfalfa [A]	31%	26%	26%	31%	31%	19%	19%	36%	36%	4%	4%	4%
	Grass	26%	22%	22%	26%	26%	32%	32%	18%	18%	51%	51%	51%
	Corn Grain [A]	3%	4%	4%	3%	3%	5%	5%	3%	3%	7%	7%	7%
	Mineral Mix[C]	2%	3%	3%	2%	2%	3%	3%	3%	3%	3%	3%	3%
	Byproduct Blend	20%	15%	15%	20%	20%	25%	25%	10%	10%	11%	11%	11%

Midwest byproduct blend = Northwest Plains (NP), Upper Midwest (UP), Great Lakes (GL); West byproduct blend = Intermountain (IM), Southwest (SW), Pacific Northwest (PNW), West; Northeast byproduct blend = Northeast (NE), New England (NEG), Mid-Atlantic (MA); South byproduct blend = Mississippi Valley (MV), Southeast (SE).
[A] Estimates based on FoodS^3 models considering county scale commodity flows and emission factors [18, 19]
[B] Estimated based on FAF5 commodity flows, supply and demand distributions; with spatially estimated emissions at state scale [20].
[C] Based on GFLI [21], literature, or other LCA databases reflecting national averages [22].

Table S9. Byproduct blends across regions.

Byproduct	Midwest	West	Northeast	South
Almond Hulls [A]	0.7%	0.0%	0.0%	7.3%
Bakery [A]	1.1%	4.2%	0.0%	0.5%
Beet Pulp [B]	0.8%	2.3%	0.0%	0.5%
Bloodmeal [B]	1.0%	1.7%	2.9%	0.8%
Brewer's Grain Dry [A]	0.0%	0.7%	0.0%	0.2%
Brewer's Grain Wet [A]	3.2%	3.9%	4.9%	2.5%
Candy [B]	0.0%	0.3%	0.0%	0.1%
Canola Meal [A]	7.1%	12.1%	1.3%	17.6%
Canola Meal TRT[A]	0.0%	3.0%	0.0%	0.1%
Cereal [A]	15.2%	0.0%	0.0%	0.4%
Chocolate [B]	0.0%	0.0%	0.0%	0.1%
Citrus Dry [A]	1.2%	1.0%	1.9%	0.2%
Citrus Wet [A]	0.0%	0.0%	0.0%	5.7%
Corn cannery [A]	20.6%	0.1%	0.0%	1.1%
DDGS[A]	6.4%	5.4%	9.3%	11.3%
DDG wet [A]	1.2%	0.1%	0.0%	11.2%
Corn germ [B]	0.0%	0.0%	0.0%	1.1%
Corn gluten feed dry [A]	1.9%	3.3%	14.8%	2.1%
Corn gluten feed wet [A]	2.8%	0.4%	0.0%	0.3%
Corn gluten meal [A]	0.2%	0.9%	0.0%	0.1%
Corn starch [B]	0.0%	2.1%	0.0%	0.0%
Corn steep [B]	0.2%	0.1%	0.0%	0.5%
Cottonseed Whole [A]	5.6%	2.5%	12.2%	10.0%
Cotton Hulls [B]	0.3%	0.0%	0.0%	0.1%
Cotton meal [B]	0.0%	0.1%	1.2%	0.3%
Animal fat [B]	0.1%	0.2%	0.0%	0.2%
Palm fat [B]	1.1%	1.9%	0.3%	0.5%
Vegetable fat [B]	0.0%	0.1%	0.0%	0.0%
Feather meal [B]	0.0%	0.1%	0.0%	0.1%
Fishmeal [B]	0.0%	0.0%	0.0%	0.0%
Hominy [B]	0.9%	0.1%	0.9%	0.4%
Linseed [B]	0.0%	0.0%	0.0%	0.8%
Malt sprouts [A]	0.6%	0.1%	0.0%	0.4%
Meat meal [B]	0.0%	0.0%	0.0%	0.0%
Meat & Bonemeal (MBM)[B]	0.5%	0.0%	0.0%	0.0%
Molasses (beet)[B]	1.7%	0.1%	0.0%	0.4%
Molasses (cane)[A]	2.5%	4.0%	0.8%	0.8%
Oat Hulls [A]	0.8%	0.2%	0.0%	0.0%
Oat Mill Feed [B]	0.0%	0.0%	0.0%	0.0%
Palm Kernel [B]	0.0%	0.0%	0.0%	0.1%
Peanut Hull [B]	0.1%	0.0%	0.0%	0.0%
Peanut meal [B]	0.0%	0.0%	0.0%	0.1%
Potato [B]	0.5%	0.0%	0.0%	0.4%
Rice Bran [B]	0.0%	0.0%	0.0%	0.2%
Rice Hull [B]	0.0%	0.0%	0.0%	0.0%
Rice Mill Feed [B]	0.0%	0.0%	0.0%	0.1%
Safflower [B]	0.0%	0.0%	0.0%	0.1%
Soy Hulls [A]	1.4%	3.7%	2.9%	1.8%
Soybean Meal [A]	9.1%	17.0%	17.0%	4.0%
Soybean Meal MRT[A]	3.9%	5.6%	1.8%	2.2%
Sugar [B]	0.0%	0.4%	0.0%	0.0%
Sunflower [B]	0.0%	0.0%	0.0%	0.1%
Wheat Bran [B]	0.0%	0.1%	0.0%	0.3%
Wheat Distillers Dry [B]	0.0%	0.0%	0.0%	0.4%
Wheat Distillers Wet [B]	0.0%	0.0%	0.0%	0.9%
Wheat flour [B]	0.0%	0.0%	0.0%	0.1%
Wheat Midds [B]	3.5%	4.9%	0.0%	0.6%
Wheat Mill Run [B]	0.2%	0.0%	0.0%	1.1%
Red dog [B]	0.0%	1.7%	0.0%	0.1%
Wheat short [B]	0.0%	0.0%	0.0%	0.0%
Whey Dry [A]	0.0%	0.0%	0.0%	0.0%
Whey Liquid [B]	2.4%	9.3%	0.0%	3.1%
Whey condensed [A]	1.0%	6.5%	27.8%	6.4%

Midwest = Northwest Plains (NP), Upper Midwest (UP), Great Lakes (GL); West = Intermountain (IM), Southwest (SW), Pacific Northwest (PNW), West; Northeast = Northeast (NE), New England (NEG), Mid-Atlantic (MA); South = Mississippi Valley (MV), Southeast (SE).
[A] See Table 17
[B] Emission factors based on GFLI [21], literature, or other LCA databases reflecting national averages [22].

Table S10. Key dietary parameters for lactating dairy cows across regions in 2020. Northwest Plains (NP), Upper Midwest (UP), Great Lakes (GL), Intermountain (IM), Southwest (SW), Pacific Northwest (PNW), West, Northeast (NE), New England (NEG), Mid-Atlantic (MA), Mississippi Valley (MV), Southeast (SE).

Dietary parameters	Regions											
	NP	UM	GL	IM	SW	PNW	West	NE	NEG	MA	MV	SE
Dry Matter Intake (kg/day)	24.1	23.8	24.1	24.7	24.2	23.9	24.8	23.3	22.3	21.6	20.0	22.9
% Dry matter	62.5%	63.4%	63.0%	75.7%	78.2%	76.6%	78.3%	65.4%	65.3%	65.1%	68.0%	68.0%
Organic Matter (OM) % DM	65.4%	67.2%	66.7%	73.2%	73.9%	73.6%	74.8%	72.6%	72.9%	73.5%	72.1%	72.1%
Crude protein (CP) % DM	14.2%	13.5%	13.8%	16.5%	16.6%	16.3%	17.3%	14.4%	14.6%	14.3%	15.0%	15.0%
Crude fat (CF) % DM	5.8%	5.1%	5.6%	7.9%	8.0%	7.8%	9.2%	4.8%	5.0%	5.1%	5.8%	5.8%
Neutral Detergent Fibers (NDF) % DM	37.4%	36.6%	36.7%	30.4%	29.9%	30.1%	28.9%	34.8%	37.0%	33.9%	32.6%	32.6%
Acid Detergent Fibers (ADF) % DM	20.3%	20.1%	19.8%	17.1%	16.6%	16.7%	15.6%	19.1%	19.7%	18.0%	17.2%	17.2%
Ash % DM	8.5%	8.5%	8.1%	9.0%	8.9%	9.0%	8.4%	8.6%	8.3%	8.3%	7.5%	7.5%
Forage % DM	51.0%	56.2%	51.3%	49.7%	46.8%	48.0%	41.1%	60.5%	61.6%	55.4%	40.8%	40.8%
Metabolizable energy (ME) [b] MJ/kg DM	10.3	10.3	10.4	11.0	11.1	11.1	11.4	10.6	10.7	10.8	11.2	11.2
Gross Energy (GE)[c] MJ/kg DM	17.6	17.6	17.6	17.6	17.6	17.6	17.6	17.6	17.6	17.6	17.6	17.6
Digestible Energy (DE) MJ/kg DM	11.9	11.9	12.0	12.6	12.8	12.7	13.0	12.3	12.3	12.5	12.9	12.9

[A] INRA-CIRAD-AFZ 2018 [23]
[B] Hales et al 2022 [24]
[C] Weiss and Tebbe 2019 [25]

Table S11. Key dietary parameters for lactating dairy cows across regions in 2007. Northwest Plains (NP), Upper Midwest (UP), Great Lakes (GL), Intermountain (IM), Southwest (SW), Pacific Northwest (PNW), West, Northeast (NE), New England (NEG), Mid-Atlantic (MA), Mississippi Valley (MV), Southeast (SE).

Dietary parameters	Regions											
	NP	UM	GL	IM	SW	PNW	West	NE	NEG	MA	MV	SE
Dry Matter Intake (kg/day)	21.4	22.2	22.8	22.8	22.6	23.3	23.3	21.8	21.6	20.9	19.5	21.2
% Dry matter	62.5%	63.4%	63.0%	75.7%	78.2%	76.6%	78.3%	65.4%	65.3%	65.1%	68.0%	68.0%
Organic Matter (OM) % DM	65.4%	67.1%	66.7%	73.2%	74.0%	73.7%	74.8%	72.6%	73.1%	73.5%	72.0%	72.0%
Crude protein (CP) % DM	14.2%	13.5%	13.8%	16.5%	16.6%	16.3%	17.3%	14.4%	14.6%	14.3%	14.9%	15.0%
Crude fat (CF) % DM	5.8%	5.0%	5.6%	7.9%	8.0%	7.8%	9.2%	4.8%	5.0%	5.1%	5.8%	5.8%
Neutral Detergent Fibers (NDF) % DM	37.4%	36.5%	36.7%	30.4%	29.9%	30.2%	28.9%	34.8%	37.0%	33.9%	32.6%	32.6%
Acid Detergent Fibers (ADF) % DM	20.3%	20.1%	19.8%	17.1%	16.6%	16.7%	15.6%	19.1%	19.8%	18.0%	17.2%	17.3%
Ash % DM	8.6%	8.6%	8.1%	9.0%	8.8%	8.8%	8.4%	8.6%	8.1%	8.3%	7.6%	7.4%
Forage % DM	51.0%	56.2%	51.3%	49.7%	46.8%	48.0%	41.1%	60.5%	61.6%	55.4%	40.8%	40.7%
Metabolizable energy (ME) [b] MJ/kg DM	10.3	10.3	10.4	11.0	11.1	11.1	11.4	10.6	10.7	10.8	11.2	11.3
Gross Energy (GE)[c] MJ/kg DM	17.6	17.6	17.6	17.6	17.6	17.6	17.6	17.6	17.6	17.6	17.6	17.6
Digestible Energy (DE) MJ/kg DM	11.9	11.9	12.0	12.6	12.8	12.7	13.0	12.3	12.4	12.5	12.9	12.9

[A] INRA-CIRAD-AFZ [23]
[B] Hales et al 2022 [24]
[C] Weiss and Tebbe 2019 [25]

Allocating Impacts of Feed Production

Animal feeds are often produced alongside several co-products, such as dried distiller grain with solubles (DDGS), which are generated alongside ethanol and corn oil co-products, or soymeal, which yield oil and hulls co-products. While some feeds are considered byproducts of the primary product (e.g. brewers' grains, almond hulls, etc.), they nonetheless contribute additional economic value to the processor, albeit modest in some cases. In concordance with FAO LEAP and IDF guidelines, the environmental impacts of crop production (e.g. corn and soybean) and subsequent processing are allocated among the resulting co-products based on their relative economic values [5, 26].

For instance, the economic values for DDGS co-products are derived from their average unit prices: $1.42 per gallon of ethanol, $113 per ton of DDGS, and $0.26 per pound of corn oil [27]. Multiplying these unit prices by the quantities produced per kilogram of processed corn grain

results in approximately $0.16/kg corn for ethanol, $0.03/kg corn for DDGS, and $0.001/kg corn for corn oil. The relative economic contribution for each product is therefore 82% for ethanol, 17% for DDGS, and 1% for corn oil. Based on these values, the shared production impacts are attributed proportionally. Where processes are specific to a particular product, such as drying DDGS, the associated impacts are directly assigned to that product.

Given the variability in product prices over time, average prices over a period of 3-5 years are recommended to ensure stability in economic allocation calculations. However, differences in emissions across studies may arise due to variations in assumed economic values, stemming from differing time spans or average methods. Despite these limitations, economic allocation is the preferred approach for the livestock and animal feed industries as it aligns impacts with the underlying purpose of production – maximizing economic value. If a co-product has no economic value, no impacts are allocated to it. Conversely, impacts are assigned in proportion to the economic value if any is realized.

Table S12. Key dietary parameters for dry dairy cows across regions in 2020

Dietary parameters	Regions											
	NP	UM	GL	IM	SW	PNW	West	NE	NEG	MA	MV	SE
Dry Matter Intake (kg/day)	13.3	13.3	13.3	13.3	13.3	13.3	13.3	13.3	13.3	13.3	13.3	13.3
% Dry matter	75.1%	66.8%	66.8%	79.3%	79.3%	81.1%	81.1%	65.1%	65.1%	83.7%	82.6%	82.6%
Organic Matter (OM) % DM	66.9%	66.7%	66.7%	70.8%	70.8%	70.7%	70.7%	69.7%	69.7%	72.8%	70.6%	70.6%
Crude protein (CP) % DM	18.7%	17.5%	17.5%	15.5%	15.5%	15.8%	15.8%	14.0%	14.0%	16.7%	17.7%	17.7%
Crude fat (CF) % DM	4.6%	4.5%	4.5%	6.1%	6.1%	5.6%	5.6%	3.9%	3.9%	5.1%	4.8%	4.8%
Neutral Detergent Fibers (NDF) % DM	45.0%	44.5%	44.5%	41.9%	41.9%	40.5%	40.5%	42.8%	42.8%	44.3%	45.3%	45.3%
Acid Detergent Fibers (ADF) % DM	25.8%	25.1%	25.1%	24.2%	5.3%	5.4%	5.4%	4.3%	4.3%	4.2%	4.4%	4.4%
Ash % DM	9.3%	8.7%	8.7%	9.4%	9.4%	9.8%	9.8%	8.9%	8.9%	9.1%	9.0%	9.0%
Forage % DM	76.8%	77.1%	77.1%	76.8%	76.8%	75.8%	75.8%	84.3%	84.3%	72.9%	72.9%	72.9%
Metabolizable energy (ME) [b] MJ/kg DM	7.7	7.5	7.5	7.7	7.7	8.0	8.0	8.2	8.2	7.4	7.4	7.4
Gross Energy (GE)[c] MJ/kg DM	17.6	17.6	17.6	17.6	17.6	17.6	17.6	17.6	17.6	17.6	17.6	17.6
Digestible Energy (DE) MJ/kg DM	9.3	9.1	9.1	9.3	9.3	9.7	9.7	9.8	9.8	9.0	9.0	9.0

Northwest Plains (NP), Upper Midwest (UP), Great Lakes (GL), Intermountain (IM), Southwest (SW), Pacific Northwest (PNW), West, Northeast (NE), New England (NEG), Mid-Atlantic (MA), Mississippi Valley (MV), Southeast (SE).

Table S13. Key dietary parameters for dry dairy cows across regions in 2007

Dietary parameters	Regions											
	NP	UM	GL	IM	SW	PNW	West	NE	NEG	MA	MV	SE
Dry Matter Intake (kg/day)	13.2	13.2	13.2	13.2	13.2	13.2	13.2	13.2	13.2	13.2	13.2	13.2
% Dry matter	75.1%	66.8%	66.8%	79.3%	79.3%	81.1%	81.1%	65.1%	65.1%	83.7%	82.6%	82.6%
Organic Matter (OM) % DM	66.9%	66.7%	66.7%	70.8%	70.8%	70.7%	70.7%	69.7%	69.7%	72.8%	70.6%	70.6%
Crude protein (CP) % DM	18.7%	17.5%	17.5%	15.5%	15.5%	15.8%	15.8%	14.0%	14.0%	16.7%	17.7%	17.7%
Crude fat (CF) % DM	4.6%	4.5%	4.5%	6.1%	6.1%	5.6%	5.6%	3.9%	3.9%	5.1%	4.8%	4.8%
Neutral Detergent Fibers (NDF) % DM	45.0%	44.5%	44.5%	41.9%	41.9%	40.5%	40.5%	42.8%	42.8%	44.3%	45.3%	45.3%
Acid Detergent Fibers (ADF) % DM	25.8%	25.1%	25.1%	24.2%	24.2%	23.8%	23.8%	24.6%	24.6%	24.5%	25.4%	25.4%
Ash % DM	9.3%	8.7%	8.7%	9.4%	9.4%	9.8%	9.8%	8.9%	8.9%	9.1%	9.0%	9.0%
Forage % DM	76.8%	77.1%	77.1%	76.8%	76.8%	75.8%	75.8%	84.3%	84.3%	72.9%	72.9%	72.9%
Metabolizable energy (ME) [b] MJ/kg DM	10.1	10.0	10.0	10.4	10.4	10.4	10.4	10.0	10.0	10.8	10.7	10.7
Gross Energy (GE)[c] MJ/kg DM	17.6	17.6	17.6	17.6	17.6	17.6	17.6	17.6	17.6	17.6	17.6	17.6
Digestible Energy (DE) MJ/kg DM	11.7	11.6	11.6	12.0	12.0	12.0	12.0	11.6	11.6	12.4	12.3	12.3

Northwest Plains (NP), Upper Midwest (UP), Great Lakes (GL), Intermountain (IM), Southwest (SW), Pacific Northwest (PNW), West, Northeast (NE), New England (NEG), Mid-Atlantic (MA), Mississippi Valley (MV), Southeast (SE).

Table S14. Key dietary parameters for heifer replacements & calves across regions for 2020.

Dietary parameters	Regions											
	NP	UM	GL	IM	SW	PNW	West	NE	NEG	MA	MV	SE
Dry Matter Intake (kg/day)	8.5	8.5	8.5	8.5	8.5	8.5	8.5	8.5	8.5	8.5	8.5	8.5
% Dry matter	77.0%	69.5%	69.5%	81.6%	81.6%	83.6%	83.6%	71.3%	71.3%	85.5%	75.3%	84.1%
Organic Matter (OM) % DM	65.6%	66.3%	66.3%	69.8%	69.8%	71.2%	71.2%	68.1%	68.1%	73.5%	70.4%	70.8%
Crude protein (CP) % DM	14.9%	14.5%	14.5%	15.5%	15.5%	15.7%	15.7%	15.3%	15.3%	14.6%	14.4%	14.4%
Crude fat (CF) % DM	4.7%	4.2%	4.2%	6.3%	6.3%	7.2%	7.2%	3.7%	3.7%	5.6%	3.9%	5.2%

Neutral Detergent Fibers (NDF) % DM	44.8%	44.8%	44.8%	45.6%	45.6%	45.1%	45.1%	42.6%	42.6%	45.6%	46.8%	46.8%
Acid Detergent Fibers (ADF) % DM	29.5%	29.2%	29.2%	30.6%	30.6%	29.3%	29.3%	28.5%	28.5%	28.5%	29.1%	29.1%
Ash % DM	10.4%	9.8%	9.8%	10.4%	10.4%	10.2%	10.2%	10.7%	10.7%	9.7%	9.2%	9.5%
Forage % DM	74.6%	78.2%	78.2%	74.6%	74.6%	66.3%	66.3%	84.6%	84.6%	59.9%	78.1%	59.9%
Metabolizable energy (ME) [b] MJ/kg DM	9.9	9.8	9.8	10.3	10.3	10.6	10.6	9.8	9.8	11.1	10.3	10.9
Gross Energy (GE)[c] MJ/kg DM	17.6	17.6	17.6	17.6	17.6	17.6	17.6	17.6	17.6	17.6	17.6	17.6
Digestible Energy (DE) MJ/kg DM	11.6	11.5	11.5	11.9	11.9	12.3	12.3	11.4	11.4	12.7	12.0	12.6

Northwest Plains (NP), Upper Midwest (UP), Great Lakes (GL), Intermountain (IM), Southwest (SW), Pacific Northwest (PNW), West, Northeast (NE), New England (NEG), Mid-Atlantic (MA), Mississippi Valley (MV), Southeast (SE).

Table S15. Key dietary parameters for heifer replacements & calves across regions for 2007.

Dietary parameters	Regions											
	NP	UM	GL	IM	SW	PNW	West	NE	NEG	MA	MV	SE
Dry Matter Intake (kg/day)	7.9	7.9	7.9	7.9	7.9	7.9	7.9	7.9	7.9	7.9	7.9	7.9
% Dry matter	77.0%	69.5%	69.5%	81.6%	81.6%	83.6%	83.6%	71.3%	71.3%	85.5%	84.1%	84.1%
Organic Matter (OM) % DM	65.6%	66.3%	66.3%	69.8%	69.8%	71.2%	71.2%	68.1%	68.1%	73.5%	70.8%	70.8%
Crude protein (CP) % DM	19.8%	17.3%	17.3%	16.4%	16.4%	16.2%	16.2%	15.1%	15.1%	17.1%	18.4%	18.4%
Crude fat (CF) % DM	4.7%	4.2%	4.2%	6.3%	6.3%	7.2%	7.2%	3.7%	3.7%	5.6%	5.2%	5.2%
Neutral Detergent Fibers (NDF) % DM	43.1%	42.9%	42.9%	39.7%	39.7%	39.7%	38.2%	38.2%	41.5%	41.5%	39.2%	40.5%
Acid Detergent Fibers (ADF) % DM	25.5%	24.9%	24.9%	23.7%	23.7%	22.1%	21.1%	24.9%	24.9%	21.6%	22.7%	22.7%
Ash % DM	10.4%	9.8%	9.8%	10.4%	10.4%	10.2%	10.2%	10.7%	10.7%	9.7%	9.5%	9.5%
Forage % DM	74.7%	78.2%	78.2%	74.7%	74.7%	66.3%	66.3%	84.6%	84.6%	59.9%	59.9%	59.9%
Metabolizable energy (ME) [b] MJ/kg DM	9.9	9.8	9.8	10.3	10.3	10.6	10.6	9.8	9.8	11.1	10.9	10.9
Gross Energy (GE)[c] MJ/kg DM	17.6	17.6	17.6	17.6	17.6	17.6	17.6	17.6	17.6	17.6	17.6	17.6
Digestible Energy (DE) MJ/kg DM	11.6	11.5	11.5	11.9	11.9	12.3	12.3	11.4	11.4	12.7	12.6	12.6

Northwest Plains (NP), Upper Midwest (UP), Great Lakes (GL), Intermountain (IM), Southwest (SW), Pacific Northwest (PNW), West, Northeast (NE), New England (NEG), Mid-Atlantic (MA), Mississippi Valley (MV), Southeast (SE).

Table S16. Key dietary parameters for bulls across regions for 2020.

Dietary parameters	Regions											
	NP	UM	GL	IM	SW	PNW	West	NE	NEG	MA	MV	SE
Dry Matter Intake (kg/day)	7.3	7.3	7.3	7.3	7.3	6.6	6.6	6.6	6.6	6.6	6.6	6.6
% Dry matter	77.0%	69.5%	69.5%	81.6%	81.6%	83.6%	83.6%	71.3%	71.3%	75.8%	75.3%	75.3%
Organic Matter (OM) % DM	65.6%	66.3%	66.3%	69.8%	69.8%	71.2%	71.2%	68.1%	68.1%	71.4%	70.4%	70.4%
Crude protein (CP) % DM	19.8%	17.3%	17.3%	16.4%	16.4%	16.2%	16.2%	15.1%	15.1%	14.3%	14.9%	14.9%
Crude fat (CF) % DM	4.7%	4.2%	4.2%	6.3%	6.3%	7.2%	7.2%	3.7%	3.7%	4.1%	3.9%	3.9%
Neutral Detergent Fibers (NDF) % DM	43.1%	42.9%	42.9%	39.7%	39.7%	38.2%	38.2%	41.5%	41.5%	43.6%	44.1%	44.1%
Acid Detergent Fibers (ADF) % DM	25.5%	29.2%	29.2%	30.6%	30.6%	29.3%	29.3%	28.5%	28.5%	28.5%	29.1%	29.1%
Ash % DM	10.4%	9.8%	9.8%	10.4%	10.4%	10.2%	10.2%	10.7%	10.7%	9.3%	9.2%	9.2%
Forage % DM	74.6%	78.2%	78.2%	74.6%	74.6%	66.3%	66.3%	84.6%	84.6%	78.1%	78.1%	78.1%
Metabolizable energy (ME) [b] MJ/kg DM	9.9	9.8	9.8	10.3	10.3	10.6	10.6	9.8	9.8	10.4	10.3	10.3
Gross Energy (GE)[c] MJ/kg DM	17.6	17.6	17.6	17.6	17.6	17.6	17.6	17.6	17.6	17.6	17.6	17.6
Digestible Energy (DE) MJ/kg DM	11.6	11.5	11.5	11.9	11.9	12.3	12.3	11.4	11.4	12.0	12.0	12.0

Northwest Plains (NP), Upper Midwest (UP), Great Lakes (GL), Intermountain (IM), Southwest (SW), Pacific Northwest (PNW), West, Northeast (NE), New England (NEG), Mid-Atlantic (MA), Mississippi Valley (MV), Southeast (SE).

Table S17. Feed consumption-based emission factors and land use change (in brackets [ ]) emission factors (kgCO2e/tonne) by dairy consumption region in 2020.

Feed Emission Factors[E]	Regions											
	NP	UM	GL	IM	SW	PNW	West	NE	NEG	MA	MV	SE
Corn[A,B]	701 [272]	433 [102]	432 [94]	507 [46]	602 [94]	507 [143]	536 [28]	1014 [667]	1331 [943]	963 [613]	650 [222]	816 [414]
Corn Silage[A,B]	254 [6]	168 [4]	156 [3]	83 [1]	108 [3]	95 [44]	87 [0.5]	163 [33]	132 [61]	182 [27]	185 [7]	159 [35]

Alfalfa[A,B]	246 [690]	179 [578]	227 [887]	134 [124]	134 [305]	129 [129]	104 [216]	165 [558]	129 [198]	168 [463]	164 [335]	127 [345]
Soymeal[A,B]	1114 [279]	1020 [134]	1101 [84]	1062 [119]	1224 [287]	1011 [127]	1082 [138]	1029 [90]	1024 [92]	1048 [208]	1048 [287]	1070 [392]
Soy Hulls[A,B]	229 [102]	245 [110]	250 [114]	254 [117]	249 [112]	270 [126]	253 [117]	242 [108]	240 107]	249 [114]	246 [110]	253 [116]
DDGS[A,B]	998 [125]	1012 [32]	1015 [31]	965 [99]	989 [46]	917 [105]	989 [66]	925 [111]	733 [206]	989 [16]	1070 [48]	932 [65]
WGS[B,D]	259 [9]	258 [9]	258 [8]	256 [9]	256 [9]	254 [10]	257 [9]	258 [9]	258 [9]	258 [8]	258 [9]	258 [7]
Canola Meal[C]	331 [214]	331 [206]	332 [213]	332 [210]	333 [207]	331 [213]	333 [215]	333 [208]	345 [219]	335 [152]	332 [136]	335 [169]
Corn Gluten Feed Meal (Wet)[B,D]	123 [3]	122 [3]	122 [3]	119 [3]	120 [3]	117 [4]	122 [3]	122 [3]	122 [2]	122 [3]	122 [3]	122 [3]
Corn Gluten Feed Dry[B,D]	353 [7]	350 [7]	350 [7]	343 [8]	345 [8]	338 [9]	350 [7]	350 [7]	349 [5]	350 [7]	350 [7]	350 [7]
Cottonseed[C,D]	302 [6]	291 [6]	293 [6]	296 [5]	292 [7]	296 [6.7]	294 [7]	295 [7]	297 [5]	291 [6]	290 [6]	291 [6]
Cereal/Bakery[C,D]	46	45	45	45	45	43	45	45	45	45	45	45
Molasses Cane[C,D]	386 [304]	467 [302]	467 [302]	470 [302]	468 [300]	467 [302]	468 [302]	468 [302]	569 [298]	468 [302]	468 [302]	468 [302]
Brewers Grain Wet[C,D]	11 [0.8]	12 [1.0]	12 [1.0]	11 [0.9]	11 [0.9]	11 [0.9]	11 [0.9]	12 [1.0]	12 [1.0]	12 [1.0]	11 [0.9]	11 [0.9]
Brewers Grain Dry[C,D]	420 [5]	426 [6]	426 [6]	420 [5]	422 [5]	422 [6]	424 [6]	426 [6]	427 [6]	426 [6]	423 [6]	425 [6]
Citrus Pulp Wet[C,D]	119 [.01]	119 [.01]	121 [.01]	120 [.01]	121 [.01]	119 [.01]	122 [.01]	122 [.01]	119 [.01]	122 [.01]	123 [.01]	122 [.01]
Corn Cannery Residue[C,D]	196	189	189	184	187	187	189	190	192	190	190	190
Almond Hulls[C,D]	79 [64]	79 [64]	79 [64]	79 [64]	79 [64]	79 [64]	79 [64]	79 [64]	79 [64]	79 [64]	79 [64]	79 [64]
Malt Sprouts[C,D]	411 [34]	452 [39]	452 [38]	416 [36]	426 [37]	427 [37]	442 [38]	452 [39]	459 [39]	456 [39]	431 [37]	449 [38]
Whey Condensed[C,D]	5408 [520]	5405 [520]	5405 [520]	5406 [520]	5405 [520]	5405 [520]	5405 [520]	5405 [520]	5414 [520]	5406 [520]	5406 [520]	5405 [520]
Whey Dry[C,D]	9551 [867]	9545 [867]	9545 [867]	9546 [867]	9545 [867]	9544 [867]	9545 [867]	9545 [867]	9559 [867]	9546 [867]	9546 [867]	9545 [867]

[A] Uses AR6 for direct and indirect N_2O, and AR5 for all upstream manufacturing. Note that upstream manufacturing emissions are primarily driven by CO2 emissions which do not change between AR5 and AR6.

[B] Based on county-scale LCA of crop production [19, 18] with supply chain models from FoodS[3.]

[C] Based on national LCA of crop production adjusted for state-specific yields with supply chain models based on FAF5 [20].

[D] Processing emissions estimated based on DMI-commissioned study.

[E] Transport emissions from feed sourcing included separately. Total emissions intensity from feed purchases (excluding transport) based on addition of unbracketed and bracketed values.

Table S18. Percent change in 5-year moving average crop yields (2020 vs 2007) [28].

Region	% Change (2020 compared to 2007)					
	Corn Silage	Alfalfa	Corn	Soymeal	Cotton	Canola
Great Lakes	9%	-2%	13%	13%	3%[A]	27%[A]
Intermountain	5%	5%	-2%	33%	3%[A]	29%
Mid-Atlantic	15%	4%	21%	38%	13%	27%[A]
Mississippi Valley	17%	0%	28%	38%	18%	27%[A]
New England	-3%	24%	14%[A]	18%[A]	3%[A]	27%[A]
Northeast	9%	5%	18%	14%	3%[A]	27%[A]
Northern Plains	49%	8%	23%	19%	3%[A]	29%

Pacific Northwest	-4%	-4%	18%	18%[A]	3%[A]	27%[A]
Southeast	9%	-	53%	26%	6%	27%[A]
Southwest	-2%	-3%	1%	15%	10%	27%[A]
Upper Midwest	21%	-1%	18%	19%	3%[A]	57%
West	4%	-4%	1%	18%[A]	15%	27%[A]
Average	16%	0%	14%	18%	3%	27%

[A] Based on U.S. average

Section S3. Enteric Fermentation

Equation S5. Estimation of dry cow enteric fermentation CH_4 (MJ/day) (from Moraes et al 2014) [29].

$$CH4 = 2.88 + 0.053 \times GEI - 0.19 \times CF$$

Where GEI is gross energy intake (MJ/day), and CF is crude fat (% of DM)

Equation S6. Estimation of heifer replacement enteric fermentation CH_4 (MJ/day) (from Moraes et al 2014) [29].

$$CH4 = -1.487 + 0.046 \times GEI + 0.032 \times NDF + 0.006 \times BW$$

Where GEI is gross energy intake (MJ/day), NDF is neutral detergent fiber (% of DM), and BW is average body weight (kg).

Equation S7. Estimation of bull enteric fermentation CH_4 (MJ/day) (from Moraes et al 2014) [29].

$$CH4 = -0.221 + 0.048 \times GEI + 0.005 \times BW$$

Where GEI is gross energy intake (MJ/day), and BW is average body weight (kg)

Section S4. Manure

Equation S8. Estimation of urinary nitrogen excretion for lactating dairy cows [30].

$$UN = -260 + 0.293 \times N + 3.99 \times DE + 0.906 \times NDF + 8.39 \times CP + 0.161 \times DIM + 0.37 \times MBW$$

Where UN is urinary nitrogen output (g/day), N is nitrogen intake (g/day), DE is digestible energy (MJ/kg DM), NDF is neutral detergent fiber (% of DM), CP is crude protein (% of DM), DIM is days in milking per head, MBW is metabolic body weight (kg).

Equation S9. Estimation of fecal nitrogen excretion for lactating dairy cows [30].

$$FN = 72.7 - 11.8 \times ME - 0.437 \times NDF + 3.52 \times CP + 0.161 \times F + 9.32 \times DMI - 0.0184 \times DIM$$

Where FN is fecal nitrogen output (g/day), N is nitrogen intake (g/day) ME is metabolizable energy (MJ/kg DM), NDF is neutral detergent fiber (% of DM), CP is crude protein (% of DM), F is portion of forage in diet (% of DM), DMI is daily dry matter intake (kg/day), DIM is days in milking per head (days).

Equation S10. Estimation of urinary nitrogen excretion for dry cows, replacement heifers, and calves [30].

$$UN = -43.8 + 0.429 \times N - 2.1 \times ME + 0.292 \times ADF + 3.35 \times CP + 0.284 \times MBW$$

Where UN is urinary nitrogen output (g/day), N is nitrogen intake (g/day) ME is metabolizable energy (MJ/kg DM), ADF is acid detergent fiber (% of DM), CP is crude protein (% of DM), MBW is average metabolic body weight (kg).

Equation S11. Estimation of fecal nitrogen excretion for dry cows, replacement heifers and calves [30].

$$FN = 107 + 0.359 \times N - 4.18 \times ME - 0.141 \times DM - 0.246 \times ADF - 1 \times ASH - 2.32 \times CP - 0.0792 \times MBW$$

Where FN is fecal nitrogen output (g/day), N is nitrogen intake (g/day) ME is metabolizable energy (MJ/kg dry matter), DM is dry matter portion of diet (%), ADF is acid detergent fiber (% of DM), Ash is ash content of diet (% of DM), CP is crude protein (% of DM), MBW is average metabolic body weight (kg).

Equation S12. Estimation of urinary nitrogen excretion for bulls [30].

$$UN = -71.2 + 0.265 \times N + 3.76 \times CP + 0.468 \times MBW$$

Where UN is urinary nitrogen output (g/day), N is nitrogen intake (g/day), CP is crude protein (% of DM), MBW is metabolic body weight (kg).

Equation S13. Estimation of fecal nitrogen excretion for bulls [30].

$$FN = 109.6 + 0.327 \times N - 6.41 \times ME - 0.276 \times DM - 1.37 \times L - 1.82 \times Ash + 0.0398 \times MBW$$

Where FN is fecal nitrogen output (g/day), N is nitrogen intake (g/day) ME is metabolizable energy (MJ/kg dry matter), L is lignin content (% of DM), Ash is ash content (% of DM), and MBW is average metabolic body weight (kg).

Equation S14. Estimation of methane from scraping, flushing and vacuuming manures from dairy barn floors [31].

$$CH4 = 0.13 \times T \times \frac{A_{barn}}{1000}$$

Where CH4 is the amount of methane generated per day (kg CH_4/day), T is the average daily barn temperature (°C) above 0°C, and A_{barn} is the area of the barn floor covered with manure (m^2).

Table S19. Estimated 2020 adjusted distribution of dairy cow manure management systems (MMS) across regions.

Regions	% of US Cows	PRP[A]	Daily Spread	Dry Lots	Solid Storage	Liquid/ Slurry	Anaerobic Lagoons	Deep Pit	Anaerobic Digester	Composting	Aerobic Treatment
Mississippi Valley	1%	30.3%	0.0%	0.0%	19.1%	1.9%	22.0%	13.8%	0.0%	9.7%	3.2%
Pacific Northwest	4%	6.1%	0.0%	2.2%	18.2%	2.4%	37.2%	7.6%	15.5%	8.1%	2.7%
Southwest	14%	9.5%	0.0%	6.3%	33.2%	4.3%	28.6%	3.6%	1.7%	9.6%	3.2%
West	19%	4.1%	0.0%	0.0%	20.6%	2.4%	43.4%	7.3%	10.5%	8.8%	2.9%
Intermountain	10%	6.3%	0.0%	0.8%	26.1%	2.6%	38.4%	6.9%	6.4%	9.4%	3.1%
New England	2%	12.2%	2.6%	0.0%	12.5%	4.0%	25.8%	21.0%	10.5%	8.5%	2.9%
Mid-Atlantic	3%	37.8%	0.7%	0.0%	11.1%	2.4%	22.4%	12.4%	0.3%	9.6%	3.2%
Southeast	2%	40.5%	0.0%	0.0%	6.6%	0.4%	32.4%	4.1%	3.6%	9.3%	3.1%
Upper Midwest	21%	13.3%	3.6%	0.0%	19.3%	4.5%	23.4%	17.9%	6.0%	9.0%	3.0%
Great Lakes	9%	13.7%	1.2%	0.0%	17.9%	3.3%	29.6%	15.6%	6.8%	8.9%	3.0%
Northern Plains	2%	12.6%	0.0%	0.0%	14.5%	1.8%	44.5%	12.4%	1.7%	9.4%	3.2%
Northeast	12%	16.4%	3.6%	0.0%	12.6%	4.1%	23.2%	21.8%	6.4%	8.9%	3.0%

[A] PRP = Pasture/Range/Paddock

Table S20. 2020 distribution of heifer replacement manure management systems across regions.

Regions	Daily Spread	Dry Lot	Liquid/Slurry	PRP[A]
Mississippi Valley	14.4%	63.9%	0.0%	21.7%
Pacific Northwest	0.0%	81.4%	0.8%	17.7%
Southwest	8.4%	91.2%	0.0%	0.4%
West	10.7%	87.5%	0.8%	1.0%
Intermountain	1.1%	98.5%	0.0%	0.4%
New England	44.1%	48.9%	0.0%	6.9%
Mid-Atlantic	20.6%	31.5%	0.0%	47.9%
Southeast	20.3%	53.0%	0.5%	26.3%
Upper Midwest	11.1%	82.4%	0.0%	6.5%
Great Lakes	9.9%	84.6%	0.0%	5.5%
Northern Plains	7.6%	87.7%	0.0%	4.8%
Northeast	45.9%	46.2%	0.0%	7.9%

[A] PRP = Pasture/Range/Paddock

Table S21. Estimated 2007 adjusted distribution of dairy cow manure management systems (MMS) across regions.

Regions	% of US Cows	PRP[A]	Daily Spread	Dry Lots	Solid Storage	Liquid/ Slurry	Anaerobic Lagoons	Deep Pit	Anaerobic Digester	Composting	Aerobic Treatment
Mississippi Valley	2%	19.2%	12.7%	0.0%	33.2%	13.3%	6.6%	3.3%	0.0%	9.9%	1.9%
Pacific Northwest	4%	15.3%	0.0%	0.0%	10.2%	18.9%	41.3%	1.2%	1.3%	9.7%	1.8%
Southwest	12%	0.3%	7.4%	0.0%	12.0%	20.4%	46.9%	1.2%	0.0%	9.8%	1.9%
West	20%	0.9%	9.5%	0.0%	7.9%	18.5%	51.1%	0.0%	0.6%	9.8%	1.8%
Intermountain	8%	0.3%	1.0%	0.0%	11.3%	20.8%	53.7%	1.2%	0.1%	9.8%	1.9%
New England	2%	5.9%	37.8%	0.0%	16.7%	14.7%	8.3%	1.8%	3.5%	9.5%	1.8%
Mid-Atlantic	4%	42.3%	18.2%	0.0%	12.7%	8.9%	4.6%	1.7%	0.0%	9.8%	1.9%
Southeast	2%	23.0%	17.8%	0.0%	7.7%	11.7%	27.6%	0.0%	0.7%	9.7%	1.8%
Upper Midwest	22%	5.7%	9.6%	0.0%	36.6%	20.9%	10.6%	3.7%	1.4%	9.6%	1.8%
Great Lakes	8%	4.7%	8.3%	0.0%	29.9%	23.8%	14.7%	3.2%	4.1%	9.4%	1.8%
Northern Plains	2%	4.1%	6.5%	0.0%	32.4%	24.4%	16.0%	3.5%	1.7%	9.6%	1.8%
Northeast	13%	6.9%	40.0%	0.0%	19.4%	12.3%	6.7%	1.7%	1.4%	9.7%	1.8%

[A] PRP = Pasture/Range/Paddock

Table S22. 2007 distribution of heifer replacement manure management systems across regions.

Regions	Daily Spread	Dry Lot	Liquid/Slurry	PRP
Mississippi Valley	14.3%	63.4%	0.0%	21.6%
Pacific Northwest	0.0%	82.1%	1.0%	17.9%
Southwest	8.4%	91.2%	0.0%	0.4%
West	10.8%	88.2%	1.0%	1.0%
Intermountain	1.1%	98.5%	0.0%	0.4%
New England	44.1%	48.9%	0.0%	6.9%
Mid-Atlantic	20.5%	31.7%	0.0%	47.6%
Southeast	20.4%	53.3%	0.6%	26.3%
Upper Midwest	11.2%	82.8%	0.0%	6.6%
Great Lakes	9.9%	84.6%	0.0%	5.5%
Northern Plains	7.6%	87.7%	0.0%	4.8%
Northeast	45.9%	46.2%	0.0%	7.9%

Table S23. Distribution of solids separation adoption by MMS and technology across regions.

	Adoption Rates in Solid-Liquid Separation			% deploying pre- or post-AD		Solids Separation type:			
**Regions**	Uncovered lagoon	liquid/slurry	AD	AD -post digestion	AD- pre digestion	Gravity (settling basin)	Stationary screen	Roller drum	Screw press
Northern Plains	10%		80%	80%	20%		100%		
Upper Midwest	50%	50%	80%	80%	20%		50%	25%	25%
Great Lakes	50%	50%	80%	80%	20%		50%	25%	25%
Intermountain	10%		80%	80%	20%		100%		
Southwest	10%		80%	80%	20%		100%		
Pacific Northwest	10%		80%	80%	20%		100%		
West	50%		80%	80%	20%	50%	50%		
Northeast	50%	50%	80%	80%	20%		50%	25%	25%
New England	50%	50%	80%	80%	20%		50%	25%	25%
Mid-Atlantic	10%		80%	80%	20%		100%		
Mississippi Valley	10%		80%	80%	20%		100%		
Southeast	50%		80%	80%	20%	50%	50%		

Table S24. Solid-liquid separation removal rates of volatile solids and nitrogen [32].

**Solid-liquid Separation**	**Volatile Solids % removal**	**Nitrogen % Removal**
Gravity (settling basin)	60%	60%
Stationary screen	20%	10%
Roller drum	25%	15%
Screw Press	25%	15%

Table S25. Total methane conversion factors by average annual ambient temperature (°C) and manure management system (MMS) adapted from [33]

Methane conversion factors by temperature °C and manure management system	Cool[C]					Temperate[C]											Warm[C]		
	≤10	11	12	13	14	15	16	17	18	19	20	21	22	23	24	25	26	27	≥28
Uncovered Anaerobic Lagoon	0.66	0.68	0.7	0.71	0.73	0.74	0.75	0.76	0.77	0.78	0.78	0.78	0.79	0.79	0.79	0.79	0.79	0.8	0.8
Liquid/Slurry[a]	0.17	0.19	0.2	0.22	0.5	0.22	0.235	0.26	0.285	0.315	0.34	0.375	0.405	0.47	0.51	0.56	0.61	0.64	0.65
Deep Pit[B]	0.17	0.19	0.2	0.22	0.25	0.15	0.16	0.175	0.19	0.21	0.225	0.245	0.265	0.29	0.315	0.34	0.3	0.3	0.3
Solids Storage	0.02	0.02	0.02	0.02	0.02	0.04	0.04	0.04	0.04	0.04	0.04	0.04	0.04	0.04	0.04	0.04	0.05	0.05	0.05
Dry lots	0.01	0.01	0.01	0.01	0.01	0.015	0.015	0.015	0.015	0.015	0.015	0.015	0.015	0.015	0.015	0.015	0.02	0.02	0.02
Pasture/range/paddock	0.01	0.01	0.01	0.01	0.01	0.015	0.015	0.015	0.015	0.015	0.015	0.015	0.015	0.015	0.015	0.015	0.02	0.02	0.02
Daily Spread	0.001	0.001	0.001	0.001	0.001	0.015	0.015	0.015	0.015	0.015	0.015	0.015	0.015	0.015	0.015	0.015	0.02	0.02	0.02
Aerobic Treatment	0	0	0	0	0	0	0	0	0	0	0	0	0	0	0	0	0	0	0
Compost	0.005	0.005	0.005	0.005	0.01	0.01	0.01	0.01	0.01	0.01	0.01	0.01	0.01	0.01	0.01	0.01	0.015	0.015	0.015

[A] Liquid/slurry systems assume no crust cover based on Greene et al 2024 [34].

[B] Warm climates assumed to store for <1 month before spreading, cool climates assumed to store manures >1 month before spreading due to winter conditions, and temperate climates exhibiting a mix of storage practices with an average of manures stored <1 month and >1 month.

C Average ambient temperatures for each county for 2020 and 2007 are based on the National Oceanic and Atmospheric Administration (NOAA) National Centers for Environmental Information

Table S26. Direct N_2O emission factors per quantity of N handled in each manure management system [4, 33]

Manure management system	Direct N2O Emission factor (kg N2O-N/kg N)
Uncovered anaerobic lagoon	0
Liquid/Slurry	0.005
Deep Pits	0.002
Solid storage	0.005
Dry lots	0.02
Pasture/Range/Paddock[A]	0.02
Daily spread	0
Anaerobic digester	0
Aerobic Treatment	0.0075
Composting[B]	0.0305

[A] No direct N_2O from management, but direct N_2O forms from manure sitting on grassland systems [35]
B Represents average across in vessel, intensive, passive, and static composting systems

Table S27. Indirect N_2O emissions based on percent of N loss to volatilization and runoff [33].

Cattle System	Manure Management System	NH3 Volatilization (% N Loss)	% of N loss to runoff/leaching				
			Central	Pacific	Mid-Atlantic	Midwest	South
Dairy Cattle	Daily Spread	10%	0.0%	0.0%	0.0%	0.0%	0.0%
	Deep Pit	24%	0.0%	0.0%	0.0%	0.0%	0.0%
	Dry lot	15%	0.6%	2.0%	1.8%	0.9%	2.2%
	Liquid/Slurry	26%	0.2%	0.8%	0.7%	0.4%	0.9%
	PRP[A]	21%[B]	0.0%	0.0%	0.0%	0.0%	0.0%
	Solid storage	27%	0.2%	0.0%	0.0%	0.0%	0.0%
	Anaerobic lagoon	43%	0.2%	0.8%	0.7%	0.4%	0.9%
	Anaerobic digester	0.0%	0.0%	0.0%	0.0%	0.0%	0.0%
	Aerobic Treatment	85%	0%	0%	0%	0%	0%
	Composting	45%	4%	4%	4%	4%	4%

[A] PRP = Pasture/Range/Paddock. Includes no volatilization from management, but indirect N_2O forms from manure sitting on grassland systems [35].
[B] 24% in areas prone to run-off, as indicated by average precipitation > evapotranspiration potential [29].

Section S5. Farm Energy

Figure S1. Electricity and thermal energy use estimated per cow across regions.

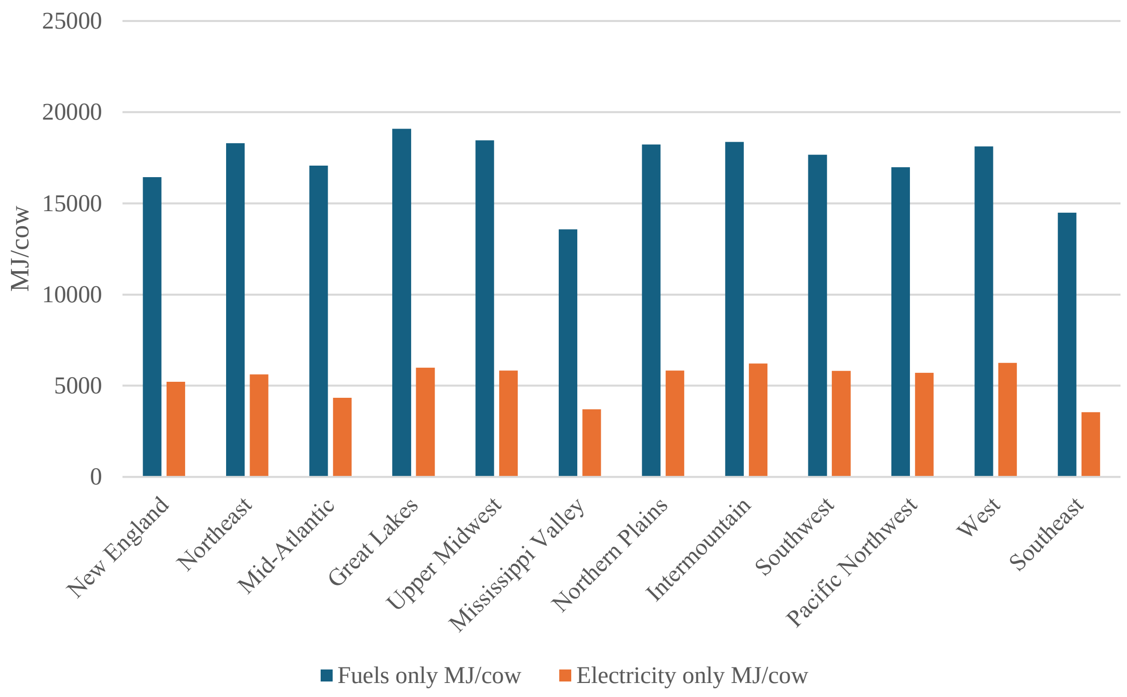

Table S28. Internal energy supply assumptions for anaerobic digester operation across biogas use scenarios.

Biogas use scenarios	Flaring		Onsite electricity generation, excess export to grid		Onsite/co-located thermal energy generation and use		On-site CHP cogeneration, excess electricity exported to grid		RNG	
Energy source[A]	Purchased Electricity	Boiler (80%)	Reciprocating engine (35%[B])	Boiler (80% [B])	Purchased Electricity	Boiler (80% [B])	Reciprocating engine CHP (35% [B])	Reciprocating engine CHP (44% [B])	Purchased Electricity	Boiler (80% [B])
Energy Consumption category	Electricity	Thermal[C]	Electricity	Thermal[C]	Electricity	Thermal[C]	Electricity	Thermal[C]	Electricity	Thermal[C]
Units	kWh/m^3 biomethane produced	m^3 biomethane/ m^3 biomethane produced	m^3 biomethane/ m^3 biomethane produced	m^3 natural gas/m^3 biomethane produced	kWh/m^3 biomethane produced	m^3 biomethane/ m^3 biomethane produced	m^3 biomethane/ m^3 biomethane produced	m^3 biomethane/ m^3 biomethane produced	kWh/m^3 biomethane produced	m3 natural gas/m^3 biomethane produced
Covered Lagoon	0.00	0.18 (0.08 - 0.68)	0.00	0.18 (0.08 – 0.68)	0.00	0.18 (0.08 – 0.68)	0.00	0.34 (0.14 – 1.24)	0.00	0.18 (0.08 – 0.68)
Complete Mix/Plug Flow	0.82	0.37 (0.16 – 1.36)	0.22	0.37 (0.16 – 1.36)	0.82	0.37 (0.16 – 1.36)	0.22	0.67 (0.28 – 2.48)	0.82	0.37 (0.16 – 1.36)

[A] For satisfying internal energy requirements for AD operation
[B] For internal energy generation, describes efficiency (output energy per unit of energy input)
[C] Based on sensible heat requirements for operation of digester systems, which depends on county average ambient temperatures [34], see equation 15
. Values in parentheses indicate range across counties.

Equation S15. Estimation of the thermal energy requirements for anaerobic digester systems to maintain constant operating temperatures.

$$E = \frac{M \times Q \times (O - T) \times (1 + (L - R)) \times S}{H \times B \times Eff \times 10^6}$$

Where E is the quantity of thermal energy inputs for operation per unit of biomethane generated (m^3 thermal requirements/m^3 CH_4 generated), M is the quantity of manure generated within the growth phases associated with anaerobic digestor usage (g/head), Q is the specific heat capacity of water (4.186 J/g C°), O is the operating temperature of the digester (36 C°), T is the ambient temperature, L is the heat loss (assuming 59% [34]), R is the heat recycle rate (assuming 50% [34]), B is the quantity of methane generated in the AD system, H is the heating value of methane (MJ/m^3), Eff is the efficiency of the boiler system, and S is the scalar for thermal energy required in covered lagoon vs. all other AD systems (0.67 for covered lagoons, 1.33 for all other AD; based on GREET 2023 [20]) and 10^6 is the conversion factor for converting J to MJ.

Section S6. Results

Table S29. Total estimated emissions by greenhouse gas (GHG) type for 2020 and 2007.

GHG contribution	Tonnes CO_2e (2020)	Tonnes CO_2e (2007)
CH_4 (biogenic)	57,423,342	50,734,785
Aggregate CO_2e[A]	45,148,497	43,227,582
N_2O	14,751,298	13,879,938
CO_2 (LUC)	14,705,366	13,864,761
CO_2 (fossil)	8,254,354	9,077,389
CH_4 (fossil)	580,962	482,069
CO_2 (sequestration)	(1,983,155)	(1,880,909)
Total	138,880,664	129,385,614
Total without sequestration	140,863,819	131,266,523

[A] Note that aggregate CO_2e cannot be disaggregated due to data limitations, however, because these emissions are from feed inputs, GHGs are likely to be split mostly between CO_2 and N_2O categories.

Table S30. Variation in emission intensity and total emissions across dairy production regions in 2020.

Regions	Kg CO_2e/kg FPCM	Tonnes FPCM	Tonnes CO2e
Northern Plains	1.51	2.3M	3.4M
Upper Midwest	1.26	21.9M	27.5M
Great Lakes	1.30	9.6M	12.5M
Intermountain	1.24	10.8M	13.3M
Southwest	1.33	14.8M	19.7M
Pacific Northwest	1.31	4.3M	5.7M
West	1.42	18.9M	26.8M
Northeast	1.65	11.4M	18.8M
New England	1.61	1.8M	3.0M
Mid-Atlantic	1.87	2.3M	4.3M
Mississippi Valley	1.86	0.7M	1.2M
Southeast	1.53	1.8M	2.7M
**US**	**1.38**	**100.50M**	**138.88M**

Section S7. Sensitivity & Uncertainty Analysis

Table S31. GWP100 carbon dioxide equivalents across IPCC assessment reports.

Gas Type	AR4	AR5 with climate carbon feedback	AR5 without climate carbon feedback	AR6
CO2	1	1	1	1
CH4 (bio)	25	34	28	27.2
CH4 (fossil)	25	36	30	29.8
N2O	298	298	265	273

Alternative models for estimating manure and enteric fermentation emissions, for comparison with the current study (Sections 3 and 4):

Manure

Equation S16. IPCC Method for Estimating Volatile Solids (for comparison to the current study) [4].

$$VS = [(GE - DE)] + (UE \times GE)] \times \frac{1 - ASH}{18.45}$$

Where VS is kg of volatile solids/day, GE is gross energy intake MJ/day, DE is digestible energy intake (MJ/day), Urinary Energy expressed as fraction of gross energy, ASH is the percent ash content expressed as the dry matter feed intake, and 18.45 is the conversion factor for dietary GE per kg of dry matter (MJ/kg).

Equation S17. IPCC Method for Estimating N excretion (for comparison to the current study) [4].

$$Nex = \left(\frac{GE}{18.45} \times \left(\frac{CP}{6.25}\right)\right) \times \left(1 - \left(\left[\frac{Milk \times (MP)}{6.38}\right] + \left[\frac{WG \times \left[268 - \left(\frac{7.03 \times NE_g}{WG}\right)\right]}{1000 \times 6.25}\right]\right)\right)$$

Where Nex is the N excretion rate (kg/day), GE is the gross energy intake (MJ/day), CP is the percent crude protein in diet, Milk is the rate of production (kg/cow/day), MP is the percent of protein in the milk, WG is the weight gain kg/day, NE is the net energy for growth (MJ/day), 6.25 is the conversion from dietary protein to dietary N (kg protein/kg N), and 6.38 is the conversion from milk protein to milk N (kg protein/kg N).

Equation S18. USDA Blue book (2024) method for estimating VS excretion [31]

$$VS = \frac{C_g}{1000} \times BW_g$$

Where VS is the volatile solids excretion rate (kg VS/day), BW is the animal mass (kg) of each growth phase, and C_g is the constant volatile solids excretion rates for each growth phase, including 7.3 kg/1000 kg animal mass/day for replacement heifers, and 11 kg/1000 kg animal mass/day for lactating dairy cows, and 5.6 kg/1000 kg animal mass/day for mature nonlactating cattle, based on the USDA Ag Waste Management Field Handbook

Equation S19. USDA Blue Book (2024) method for estimating N excretion from lactating cows [31] (for comparison to the current study).

$$Nex = \left(DMI \times \left(\frac{CP}{6.25}\right) \times 0.66\right) + 3.03$$

Where Nex is the N excretion rate (g/day) DMI is the dry matter intake (kg/day), CP is the dietary crude protein concentration (g/kg of DM), 6.25 is the conversion from g of dietary crude protein to g of dietary nitrogen.

Equation S20. USDA Blue Book (2024) method for estimating N excretion from nonlactating cows (for comparison to the current study) [31].

$$Nex = \left(DMI \times \left(\frac{CP}{6.25}\right) \times 0.828\right) + 15.1$$

Where Nex is the N excretion rate (g/day) DMI is the dry matter intake (kg/day), CP is the dietary crude protein concentration (g/kg of DM), 6.25 is the conversion from g of dietary crude protein to g of dietary nitrogen.

Equation S21. ASAE (2005) method for estimating N excretion from lactating cows (for comparison to the current study) [36].

$$Nex = 2.303 \times MP + 0.159 \times DIM + 70.138 \times DMI \times CP + 0.193 \times BW - 56.632$$

Where Nex is the nitrogen excretion rate (g/day), MP is the milk production rate (kg/day), DIM is the number of days in milk, DMI is the dry matter intake for the animal class (kg/day), BW is the animal live weight (kg), CP is the concentration of crude protein in the diet (% of DM).

Equation S22. ASAE (2005) method for estimating N excretion from dry cows (for comparison to the current study) [36].

$$Nex = 12.747 \times DMI + 1606.29 \times CP - 117.5$$

Where Nex is the nitrogen excretion rate (g/day), DMI is the dry matter intake for the animal class (kg/day), CP is the concentration of crude protein in the diet (% of DM).

Equation S23. ASAE (2005) method for estimating N excretion from heifers (for comparison to the current study) [36].

$$Nex = 78.39 \times DMI \times CP + 51.35$$

Where Nex is the nitrogen excretion rate (g/day), DMI is the dry matter intake for the animal class (kg/day), CP is the concentration of crude protein in the diet (% of DM).

Enteric Fermentation

Equation S24. Ellis (2007) method using DMI for estimating enteric fermentation methane emissions as used in Thoma et al 2013 (for comparison to the current study) [37, 38].

$$CH4 = 0.809 \times DMI + 3.23$$

Where CH4 is enteric fermentation emissions in MJ/day, and DMI is dry matter intake (kg/day).

Equation S25. Ellis (2007) method using forage for estimating enteric fermentation methane emissions as used in Capper and Caddy 2020 (for comparison to the current study) [37, 1].

$$CH4 = 0.139 \times F + 8.56$$

Where CH4 is enteric fermentation emissions in MJ/day, and F is the percent of forage in the diet (% on dry matter basis).

Equation S26. IPCC (2019) tier 2 energy balance method for estimating enteric fermentation methane emissions (for comparison to the current study) [4].

$$CH4 = GE \times Y_m$$

Where CH4 is MJ/day, GE is gross energy intake MJ/day, and Ym is the methane conversion factor (percent of gross energy in feed converted to methane). For high producing cows (>8500 kg/head/yr) Ym is specified as either 5.7% for NDF values less than or equal to 35% of diet dry matter (DM), and 6% for NDF values greater than 35% DM.

Equation S27. IPCC (2019) simplified tier 2 method for estimating enteric fermentation methane emissions (for comparison to the current study) [4].

$$CH4 = DMI \times \frac{MY}{1000} \times 55.65$$

Where CH4 is MJ/day, MY is the methane yield g CH4/kg DMI, and 55.65 is the energy content of methane (MJ/kg CH4).

Equation S28. USDA Blue Book (2024) method for estimating enteric fermentation methane emissions for lactating cows, as based on Niu et al 2018 equation 34 (for comparison to the current study) [31].

$$CH4 = \frac{-126 + 11.3 \times DMI + 2.3 \times NDF + 28.8 \times MF + 0.148 \times BW}{1000} \times 55.65$$

Where CH4 is MJ/day, DMI is dry matter intake (kg/day), NDF is dietary neutral detergent fiber concentration (% of DM), MF is milk fat concentration (%), and BW is body weight (kg), and 55.65 is the energy content of methane (MJ/kg CH4).

Equation S29. USDA Blue Book (2024) method for estimating enteric fermentation methane emissions for dry cows, as based on Moraes et al 2014 'GE level equations' (for comparison to the current study) [29, 31].

$$CH4 = 2.381 + 0.053 \times GE$$

Where CH4 is MJ/day, GE is dietary gross energy intake (MJ/day).

Equation S30. USDA Blue Book (2024) method for estimating enteric fermentation methane emissions for heifers, as based on Moraes et al 2014 'GE level equations' (for comparison to the current study) [29, 31].

$$CH4 = 1.289 + 0.051 \times GE$$

Where CH4 is MJ/day, GE is dietary gross energy intake (MJ/day).

Table S32. Estimated weighted average and range of dVS, urinary N and fecal N excreted per day across regions in this study.

Cattle Category	dVS kg/day	Fecal N g/day	Urinary N g/day	Total N g/day
Lactating Dairy Cows	5.2[a] Range: 2.1 – 5.8	151[a] Range: 109 – 159	169[a] Range: 138 – 200	320[a] Range: 247 – 353
	[6.1[a] Range: 4.7 – 6.8][b]	NA		[468[a] Range: 404 – 545]
	{7.5}[c]	NA		{392[a] Range: 318 – 456}
Dry Cows	2.4[a] Range: 2.2 – 2.5	187[a] Range: 183 – 207	126[a] Range: 105 – 150	313[a] Range: 274 – 356
	[5.4[a] Range: 5.1 – 5.7][b]			[274[a] Range: 239 – 322]
	{3.8}[c]			{300[a] Range: 262 – 345}
Heifer Replacements	1.1[a] Range: 1.0 – 1.3	128[a] Range: 121 – 131	58[a] Range: 52 – 60	186[a] Range: 173 – 190
	[2.4[a] Range: 2.0 – 2.5][b]			[192[a] Range:183 – 199]
	{4.3}[c]			{185[a] Range: 177 – 191}
Bulls	0.7[a] Range: 0.6 – 0.9	111[a] Range: 99 – 128	59[a] Range: 50 – 72	170[a] Range: 149 – 200
	[2.0[a] Range: 1.7 – 2.2][b]			[173[a] Range: 141 – 217]
	{5.3}[c]			{168[a] Range: 140 – 207}

[ ] indicates comparison using IPCC 20019 guidelines [4] (see Supplemental Information equation 16 and 17). This method is deployed for the EPA U.S. national GHG inventories [33].
{ } indicates comparison using the recent USDA Blue Book (2024) guidelines for quantifying GHG fluxes in agriculture and forestry (see Supplemental Information equation 18-20) [31].
[a] Represents weighted average
[b] Represents total volatile solids
[c] USDA blue book (2024) assumes 11 kg VS/1000 kg animal/day for lactating cows, 5.6 kg VS/1000 kg animal/day for dry cows, and 7.3 kg VS/1000 kg animal/day for heifers [31]

Table S33. Comparison of enteric methane and manure emissions across models, including range across regions and weighted average (in bold).

Model Comparisons	Enteric Methane	Nitrogen Excretion	Manure Methane
This study (average)	**17.4** (16.2 – 18.0) MJ/day[A]<br><br>**0.78** (0.75 – 1.24) MJ/kg FPCM[B]	**320** (247 – 353) g/day[A]	**7.1** (4.8 – 10.3) g/kg FPCM[B]
This study (upper uncertainty)	**21.6 (**19.5 – 22.3) MJ/day[A]<br><br>**0.92** (0.88 – 1.43) MJ/kg FPCM[B]	**480** (406 – 515) g/day[A]	**7.5** (5.3 – 10.9) g/kg FPCM[B]
This study (lower uncertainty)	**13.3** (12.7 – 13.7) MJ/day[A]<br><br>**0.63** (0.61 – 1.04) MJ/kg FPCM[B]	**159** (87 – 190) g/day[A]	**6.6** (4.3 – 9.7) g/kg FPCM[B]
Ellis (2007) DMI model (used in Thoma et al 2013)	**22.6** (19.4 – 23.3) MJ/day[A]<br><br>**0.93** (0.90 – 1.40) MJ/kg FPCM[B]	N/A	N/A
Mills et al (2003) Mits3 model (used in Rotz et al 2021)	**26.7** (24.5 – 27.8) MJ/day[A]<br><br>**1.13** (1.09 – 1.72) MJ/kg FPCM[B]	N/A	N/A

Ellis (2007) forage model (used in Capper and Cady 2020)[A]	**8.63** (8.62 – 8.65) MJ/day[A]<br><br>**0.48** (0.46 – 0.81) MJ/kg FPCM[B]	N/A	N/A
USDA Blue Book (Leytem et al 2024)	**13.8** (11.3 – 14.2) MJ/day[A]<br><br>**0.65** (0.63 – 0.96) MJ/kg FPCM[B]	**392** (318 – 456) g/day[A]	**10.1** (7.7 – 14.0) g/kg FPCM[B]
IPCC 2006/2019 (used in EPA 2023)	*Energy Balance Method*<br>**24.5** (20.0 – 25.5) MJ/day[A]<br><br>**0.97** (0.93 – 1.41) MJ/kg FPCM[B]<br><br>*Simplified Method*<br>**25.8** (21.1 – 26.9) MJ/day[A]<br>Range: MJ/day[A]<br><br>**1.02** (0.98 – 1.48) MJ/kg FPCM[B]	**468** (404 – 546) g/day[A]	**8.6** (7.2 – 11.7) g/kg FPCM[B]
ASAE (2005) model (used in Thoma et al 2013)	N/A	**555** (478 – 598) g/day[A]	N/A
Thoma et al 2013	1.02 – 1.17 MJ/kg FPCM[C]	430 g/day [D]	7.5 - 27 g/kg FPCM[C]

[A] Based on lactating dairy cow diets specified in this study
[B] Based on diets specified in this study across all cattle categories
[C] Based on diets within referenced study across all cattle categories
[D] Based on lactating cow diets within referenced study

Figure S2. Comparison of manure and enteric methane emissions and nitrogen excretion across models [A].

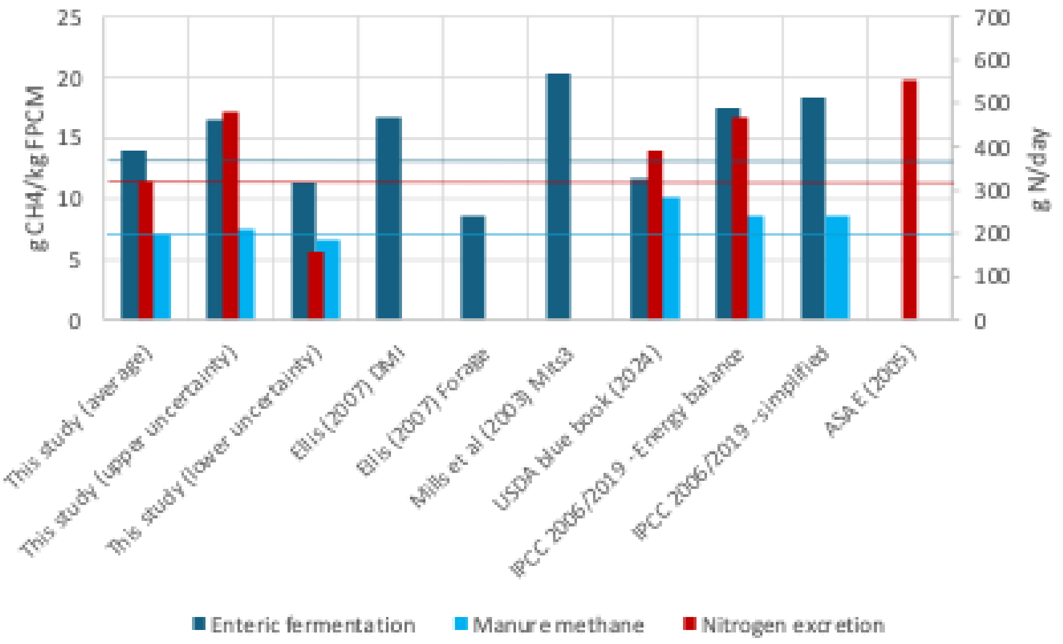

[A] Longitudinal lines are based on this study's average for ease of comparison to the alternative model estimates. Note that the 'upper uncertainty' and 'lower uncertainty' here refers to this study's upper and lower bound standard errors on parameters for estimating manure volatile solids and nitrogen excretion.

Our study estimates enteric methane emissions for lactating dairy cows to range between 16 to 18 MJ CH_4/day across regions, with an uncertainty range spanning from 13 to 22 MJ CH_4/day. These values are relatively comparable to the estimates from the Ellis (2007) dry matter intake parameter model used by Thoma et al. (2013) [37, 38], which range from 19 to 23 MJ CH_4/day. In contrast, the Mills et al. (2003) Mits3 model, applied in the IFSM model in Rotz et al. (2021) [39], and the IPCC 2006/2019 models used in the U.S. EPA national GHG inventories [4, 33], produce higher estimates of 25 to 28 MJ CH_4/day and 20 to 26 MJ CH_4/day, respectively. On the lower end, the Ellis et al (2007) forage model, used in the Capper and Cady (2019) study [1], estimates significantly lower emissions at approximately 8.6 MJ CH_4/day. Similarly, the USDA 'Blue Book' (2024) model [31], which references the Niu et al (2018) study [40] but with a higher root mean square error (RMSE) than the model used in this study, yields a range of 11-14 MJ CH_4/day. When examining total enteric methane over the entire lifecycle, our study estimates an average of 0.78 MJ/kg FPCM across different regions, 24-33% lower compared to the estimates from Thoma et al 2013, largely due to differences in dietary assumptions, growth parameters and the enteric methane models used [38]. Our approach leverages the latest and most reliable enteric methane models, minimizing RMSE as demonstrated by Niu et al (2018) [40], and incorporates a wider variety of dietary inputs across 12 distinct geographic regions, ostensibly resulting in a more refined estimate of enteric methane emissions. Our results suggest that while enteric fermentation remains a significant source of emissions, it may be less of a contributor than previously estimated in studies using less contemporary and less detailed regional analyses.

For manure methane, previous dairy LCAs and the EPA's National GHG Inventory have used total volatile solids (VS) outputs to estimate manure emissions. This approach includes all organic matter, such as lignin, which is resistant to anaerobic digestion and does not contribute to methane production, potentially leading to an overestimation of emissions. Indeed, our study estimates a range of 4.8 to 10.3 g CH_4/kg FPCM, with an uncertainty range spanning between 4.3 to 10.9 g CH_4/kg FPCM. In comparison, the IPCC (2019) model [4] used by the EPA National GHG Inventories [33], and the USDA Blue Book (2024) model [31] yield higher estimates, ranging from 7.2 to 11.7 g CH_4/kg FPCM and 7.7 to 14.0 g CH_4/day, respectively, due to the greater amount of volatile solids assumed (see Table 32). Notably, Thoma et al (2013) reported an even higher range of 7.5 to 27 g CH_4/kg FPCM across five regions [38].

For manure N_2O, our study separates urinary and fecal N excretion, allowing for a more precise calculation of total nitrogen compared to traditional models that rely solely on total nitrogen (TN). This method accounts for the different ways cattle process and excrete nitrogen, resulting in more accurate overall estimates [30]. Additionally, separating urinary N from fecal N allows for more precise estimates of indirect N_2O emissions from volatilization, as only urinary N is subject to this process. In our study, nitrogen excretion for lactating dairy cows ranges from 247 to 353 g/day, with an uncertainty range of 87 to 515 g/day. This aligns reasonably well with estimates from the USDA Blue Book (2024) models [31], which range from 318 to 456 g/day. By contrast, the ASAE (2005) model [36] used by Thoma et al. (2013) [38] and the IPCC models [4] estimate nitrogen excretion to be higher, between 478 to 598 g/day and 404 to 546 g/day, respectively. Overall, our study's lower estimates for CH_4 and N_2O emissions from manure compared to previous studies highlight that while manure management is an important source of emissions, its contribution may be less significant than suggested by earlier assessments.

Figure S3. Uncertainty analysis considering uncertainty bounds in feed emission factors, dry matter intake, enteric fermentation models, dVS models and nitrogen excretion models, and implication for total estimated A) CO_2e emission intensity across regions, B) total MT CO_2e emissions across regions, and C) total MT CO_2e emissions across the US.

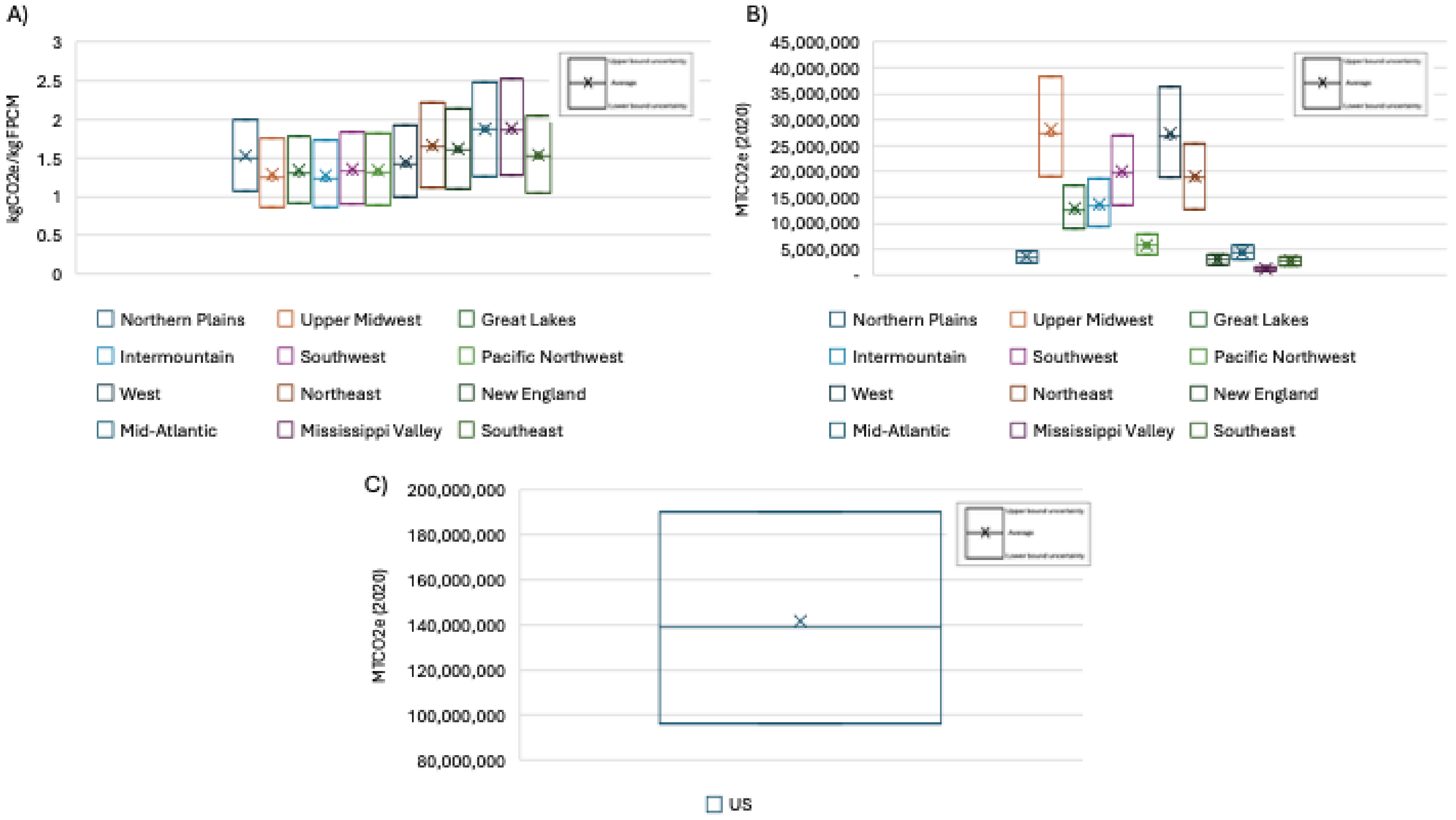

Figure S4. Contribution of emission sources across regions, considering A) upper bound standard deviation, B) average parameter values, C) lower bound standard deviation on feed emission factors, dry matter intake, enteric methane formation, volatile solids and nitrogen excretion.

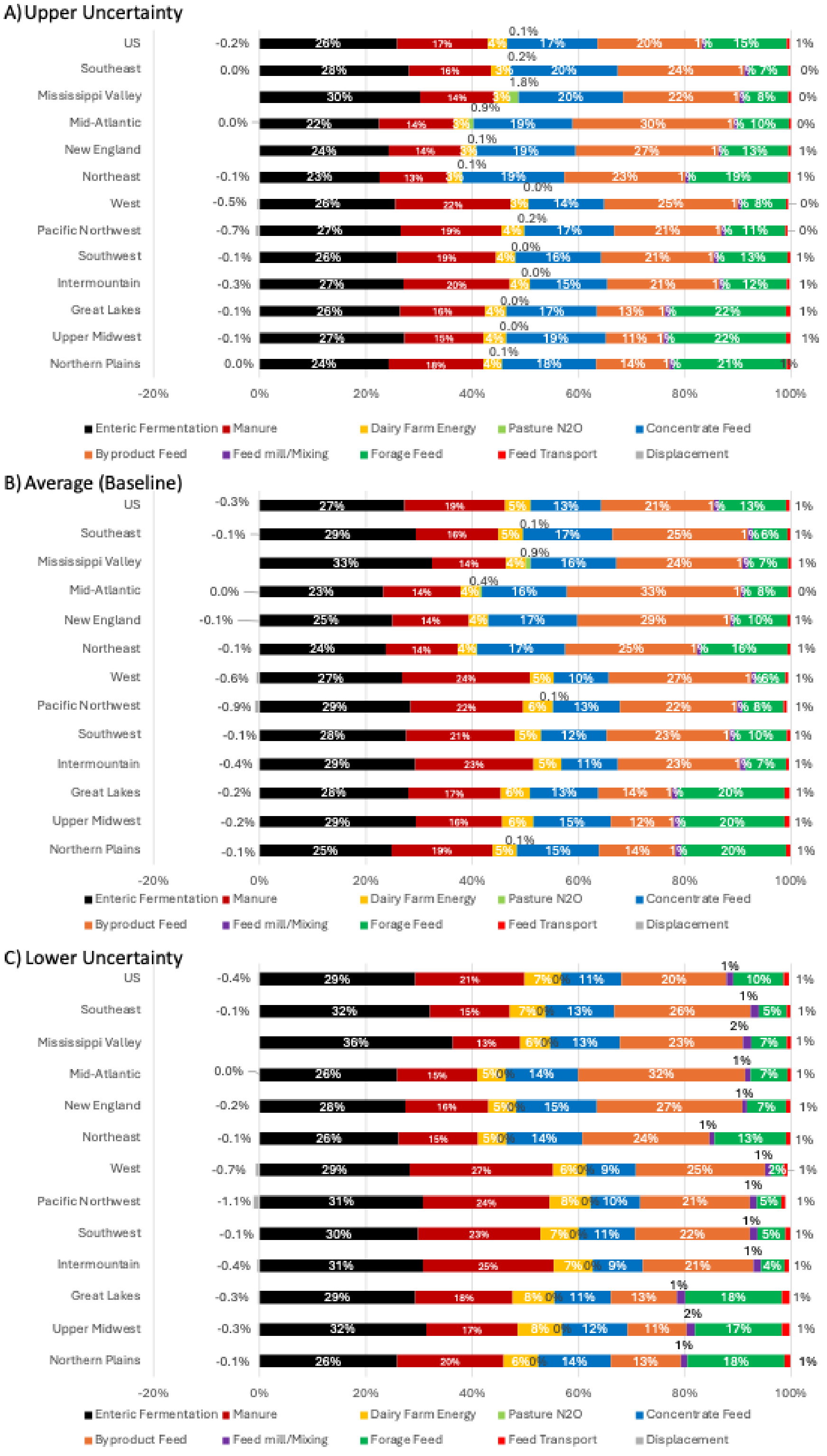

Table S34. Total annual emissions (2020) with upper and lower bound uncertainty considerations.

Uncertainty Parameters	Feed Emission Factors		Feed Dry Matter Intake		Enteric fermentation Methane formation		Manure (VS and N extraction outputs)		All Scenario uncertainty	
Regions	Upper Uncertainty	Lower Uncertainty	Upper Uncertainty	Lower Uncertainty	Upper Uncertainty	Lower Uncertainty	Upper uncertainty	Lower Uncertainty	Upper uncertainty	Lower Uncertainty
Northern Plains	3,889,919	2,936,169	3,742,019	3,085,509	3,569,411	3,258,092	3,498,158	3,329,982	4,520,862	2,431,502
Upper Midwest	32,798,488	23,230,284	30,027,752	24,882,271	28,936,209	25,973,601	28,173,292	26,744,429	38,246,993	19,031,326
Great Lakes	14,729,823	10,735,957	13,712,025	11,346,003	13,177,349	11,880,582	12,853,558	12,207,621	17,168,522	8,825,718
Intermountain	15,894,688	11,438,752	14,613,436	12,068,883	14,071,747	12,610,430	13,708,484	12,977,310	18,592,578	9,335,018
Southwest	23,192,384	16,391,289	21,558,789	17,818,929	20,689,775	18,687,752	20,229,710	19,152,024	27,052,894	13,412,435
Pacific Northwest	6,761,091	4,761,178	6,247,188	5,153,596	6,000,110	5,400,621	5,862,196	5,540,722	7,904,213	3,878,250
West	31,204,811	22,863,964	29,354,660	24,188,711	28,111,403	25,431,716	27,451,734	26,097,506	36,384,813	18,792,614
Northeast	21,990,629	15,250,333	20,640,877	17,025,013	19,633,203	18,032,540	19,220,767	18,449,152	25,378,441	12,693,647
New England	3,399,777	2,442,775	3,259,971	2,688,903	3,104,804	2,844,045	3,039,464	2,910,303	3,935,645	2,028,469
Mid-Atlantic	4,922,523	3,508,860	4,696,744	3,875,108	4,455,829	4,115,994	4,402,060	4,170,484	5,696,364	2,905,194
Mississippi Valley	1,407,220	1,028,271	1,329,283	1,098,611	1,275,804	1,152,078	1,256,467	1,171,690	1,650,200	832,193
Southeast	3,049,640	2,269,370	2,929,355	2,418,897	2,809,828	2,538,402	2,764,832	2,583,941	3,581,551	1,841,675
US	163,240,994	116,857,202	152,112,100	125,650,434	145,835,474	131,925,855	142,460,721	135,335,164	190,113,075	96,008,040

Table S35. Emission intensity (2020) with upper and lower bound uncertainty considerations.

Uncertainty Parameters	Feed Emission Factors		Feed Dry Matter Intake		Enteric fermentation Methane formation		Manure (VS and N extraction outputs)		All Scenario uncertainty	
Regions	Upper Uncertainty	Lower Uncertainty	Upper Uncertainty	Lower Uncertainty	Upper Uncertainty	Lower Uncertainty	Upper uncertainty	Lower Uncertainty	Upper uncertainty	Lower Uncertainty
Northern Plains	1.72	1.29	1.65	1.36	1.57	1.44	1.54	1.47	1.99	1.07
Upper Midwest	1.50	1.06	1.37	1.14	1.32	1.19	1.29	1.22	1.75	0.87
Great Lakes	1.53	1.12	1.43	1.18	1.37	1.24	1.34	1.27	1.79	0.92
Intermountain	1.48	1.06	1.36	1.12	1.31	1.17	1.27	1.21	1.73	0.87
Southwest	1.57	1.11	1.46	1.21	1.40	1.26	1.37	1.30	1.83	0.91
Pacific Northwest	1.56	1.10	1.44	1.19	1.38	1.24	1.35	1.28	1.82	0.89
West	1.65	1.21	1.55	1.28	1.49	1.35	1.45	1.38	1.93	0.99
Northeast	1.92	1.33	1.81	1.49	1.72	1.58	1.68	1.61	2.22	1.11
New England	1.84	1.32	1.77	1.46	1.68	1.54	1.65	1.58	2.13	1.10
Mid-Atlantic	2.15	1.53	2.05	1.69	1.95	1.80	1.92	1.82	2.49	1.27
Mississippi Valley	2.16	1.58	2.04	1.68	1.95	1.76	1.92	1.79	2.53	1.27
Southeast	1.74	1.30	1.67	1.38	1.60	1.45	1.58	1.48	2.04	1.05
US	1.62	1.16	1.51	1.25	1.45	1.31	1.42	1.35	1.89	0.96

Table S36. Comparison of the total cradle-to-farmgate million metric tonnes (MMT) of CO2e emissions and emissions intensity (kgCO_2e/kg FPCM) without displacement credits from biogas utilization, land use change (LUC), and with silage storage N_2O. Values in [ ] represents the percent change in emissions through exclusion of emission source.

Regions	Total		Without Displacement		Without LUC		With silage storage N2O	
	MMT	EF	MMT	EF	MMT	EF	MMT	EF
Northern Plains	3.41	1.51	3.42 [0.1%]	1.51	3.06 [-10.3%]	1.35	3.47 [1.8%]	1.53
Upper Midwest	27.45	1.26	27.51 [0.2%]	1.26	24.27 [-11.6%]	1.11	28.12 [2.4%]	1.29
Great Lakes	12.53	1.31	12.55 [0.2%]	1.31	11.29 [-9.9%]	1.17	12.77 [2.0%]	1.33
Intermountain	13.34	1.24	13.39 [0.4%]	1.24	12.20 [-8.5%]	1.13	13.69 [2.6%]	1.27
Southwest	19.69	1.33	19.71 [0.1%]	1.33	17.88 [-9.2%]	1.21	20.12 [2.2%]	1.36
Pacific Northwest	5.70	1.32	5.75 [0.9%]	1.32	5.14 [-9.8%]	1.18	5.82 [2.1%]	1.34
West	26.77	1.43	26.93 [0.6%]	1.43	24.81 [-7.3%]	1.31	27.18 [1.5%]	1.44
Northeast	18.83	1.65	18.85 [0.1%]	1.65	16.92 [-10.1%]	1.48	19.19 [1.9%]	1.68
New England	2.97	1.61	2.98 [0.1%]	1.61	2.67 [-10.4%]	1.45	3.01 [1.3%]	1.63
Mid-Atlantic	4.29	1.87	4.29 [0.0%]	1.87	3.92 [-8.5%]	1.71	4.32 [0.7%]	1.89
Mississippi Valley	1.21	1.86	1.21 [0.0%]	1.86	1.11 [-8.7%]	1.70	1.22 [0.5%]	1.87
Southeast	2.67	1.53	2.68 [0.1%]	1.53	2.38 [-11.0%]	1.36	2.69 [0.5%]	1.53
US	138.88	1.39	139.26 [0.3%]	1.39	125.52 [-9.6%]	1.25	141.61 [2.0%]	1.41

Table S37. Sensitivity analysis of total 2020 dairy cradle-to-farmgate emissions and contribution by GHG type across IPCC Assessment Reports (AR) GWP100 factors.

GHGs ($MTCO_2e$)	AR6[A]	AR5 with Climate Carbon Feedback [B]	AR5 no Climate Carbon Feedback [C]	AR4[D]
Aggregate CO_2e[E]	45.2M<br>33%	45.2M<br>29%	45.2M<br>32%	45.2M<br>33%
N_2O	14.8M<br>11%	15.5M<br>10%	14.5M<br>10%	15.5M<br>11%
CH_4 (biogenic)	57.4M<br>41%	71.8M<br>47%	59.1M<br>42%	52.8M<br>39%
CH_4 (fossil)	0.6M<br>0.4%	0.7M<br>0.5%	0.6M<br>0.4%	0.5M<br>0.4%
CO2 (LUC)	14.7M<br>11%	14.7M<br>10%	14.7M<br>10%	14.7M<br>11%
CO2 (fossil)	8.3M<br>6%	8.3M<br>5%	8.3M<br>6%	8.3M<br>6%
CO2 (sequestration)	-2.0M<br>-1%	-2.0M<br>-1%	-2.0M<br>-1%	-2.0M<br>-1%
Total	138.88M	154.1M	140.3M	134.9M
$kgCO_2e$/kg FPCM	1.38	1.53	1.40	1.34

[A] CH_4 (biogenic) GWP = 27, CH_4 (fossil) GWP = 29.8, N_2O GWP = 273
[B] CH_4 (biogenic) GWP = 34, CH_4 (fossil) GWP = 36, N_2O GWP = 298
[C] CH_4 (biogenic) GWP = 28, CH_4 (fossil) GWP = 30, N_2O GWP = 265
[D] CH_4 (biogenic and fossil) = 25, N_2O GWP = 298
[E] From feed emissions factors that are unable to be disaggregated by GHG type due to data source limitations.

Table S38. Combined uncertainty in emission intensity and annual total emission estimates in 2007 and 2020.

Uncertainty	Emission intensity (kgCO2e/kg FPCM)		Annual emissions (million MT CO2e)	
	2007	2020	2007	2020
Upper uncertainty	2.20	1.89	174	190
Average	1.59	1.38	126	139
Lower uncertainty	1.05	0.96	83	96

Section S8. Data quality

Table S39. Summary on meeting ISO 14044 data quality criteria requirements (based on goal and scope of this study).

Data quality requirements	Description of fulfillment of criteria in study
Temporal coverage	Study specifies time periods for data collection, comparing emissions from 2020 and 2007. It provides details on the length of time spent in each growth phase and cattle category (e.g. 968 days in 2020 and 1087 days in 2007) and uses the most up-to-date GWP factor data with noted limitations for some feed emission factors.
Geographic coverage	Study considers emissions across 12 regions of the contiguous U.S. with data inputs capturing regional variations in diet, milk production rates, manure practices and other performance metrics and farm practices. Regional differences are accounted for across practices and environmental conditions.
Technological coverage	Study incorporates advancements in methodologies such as regionalization of life cycle inventories, and updated models for enteric fermentation, manure management and crop production. Manure management practices reflect complexity of use across multiple systems. These updates reflect technological representativeness of the data used.
Precision and completeness	Study uses detailed calculations to estimate emissions from each source, accounting for detailed feed diets and interactions with enteric fermentation and manure emissions. Study accounts for comprehensive system boundary given goal and scope, with comprehensive accounting of inputs and outputs at each stage.
Representativeness	Study leverage inputs from USDA census and surveys on agricultural production practices, milk production, milk fat and crude protein, and input from industry experts and aggregate farmer data on feed practices from DMI, supporting representativeness of data inputs.
Consistency	The study applies consistent methodologies across regions and time periods, using the same functional unit (1 kg of fat and protein corrected milk, FPCM) and comparable emission factors and models. This ensures methodological consistency.
Documentation of Data quality	The study provides transparency by documenting the sources of data, assumptions, uncertainties, and limitations.

References

[1] J. Capper and R. Cady, "The effets of improved performance in the U.S. dairy cattle industry on environmental impacts between 2007 and 2017," *Journal of Animal Science,* vol. 98, no. 1, pp. 1-13, 2020.

[2] USDA, "Agricultural Census: Inventory of Milk Cows," 2017.

[3] NASEM, "Nutrient Requirements of Dairy Cattle: 8th revised edition," National Academies of Sciences, Engineering, and Medicine, 2021.

[4] O. Gavrilova, A. Leip, H. Dong, J. MacDonald, C. Bravo, B. Amon, R. Rosale, A. Prado, M. Lima, W. Oyhantcabal, T. van der Weerden and Y. Widiawati, "Chapter 10: Emissions from Livestock and Manure Management," in *2019 Refinement to the 2006 IPCC Guidelines for National Greenhouse Gas Inventories - Volume 4: Agriculture, Forestry, and Other Land Use,* 2019, pp. 10.9-10.180.

[5] International Dairy Federation (IDF), "The IDF global Carbon Footprint standard for the dairy sector," *Bulletin of the International Dairy Federation,* vol. 520, pp. 1-106, 2022.

[6] USDA NASS, "Livestock Slaughter 2020 Summary," United States Department of Agriculture National Agricultural Statistics Service, 2021.

[7] M. Van Amburgh, R. Collao-Saenz, R. Higgs, D. Ross, E. Recktenwald, L. Raffrenator, R. Chase, J. Overto, J. Mills and A. Foskolos, "The Cornell Net Carbohydrate and Protein System: Updates to the model and evaluation of version 6.5," *Journal of Dairy Science,* vol. 98, pp. 6361-6380, 2015.

[8] W. Gaines and F. Davidson, "Relation between percentage fat content and yield of milk," *Bulletin 245, University of Illinois Agriculture Experimental Station,* 1923.

[9] M. de Ondarza and J. Tricarico, "Nutritional contributions and non-CO2 greenhouse as emissions from human-inedible byproduct feeds consumed by dairy cows in the United States," *Journal of Cleaner Production,* vol. 315, no. 128125, p. 107358, 2021.

[10] FARM (Farmers Assuring Responsible Management), 2022. [Online]. Available: https://nationaldairyfarm.com/. [Accessed 3 January 2025].

[11] USDA NASS, "Milk Disposition and Income Final Estimates 2003-2007," *Statistical Bulletin* , vol. 1027, pp. 1-26, 2009.

[12] USDA NASS, "Milk Production, Disposition, and Income 2022 Summary," 2023.

[13] USDA, "Producer Milk Components Report," 2021. [Online]. Available: https://mymarketnews.ams.usda.gov/filerepo/sites/default/files/3462/2021-12-30/551255/ams_3462_00014.pdf. [Accessed 3 January 2025].

[14] USDA ERS, "Dairy Data," 2024. [Online]. Available: https://www.ers.usda.gov/data-products/dairy-data/dairy-data. [Accessed 3 January 2025].

[15] USDA, "Federal Milk Order Market Statistics 2020 Annual Summary," *Statistical Bulletin,* vol. 1021, 2022.

[16] NRC, "Nutrient Requirements of Dairy Cattle," National Academy Press, Washington, DC, 2001.

[17] A. Asselin-Balencon, J. Popp, A. Henderson, M. Heller, G. Thoma and O. Jolliet, "Dairy farm greenhouse gas impacts: A parsimonius model for a farmer's decision support tool," *International Dairy Journal,* vol. 31, pp. S65-S77, 2013.

[18] R. Pelton, T. Lark, S. Spawn, N. Springer and J. Schmitt, "Land use leverage points to reduce GHG emissions in U.S. agricultural supply chains," *Environmental Research Letters,* vol. 16, p. 115002, 2021.

[19] R. Pelton, C. Kazanski, S. Keerthi, K. Racette, S. Gennet, N. Springer, E. Yacobson, M. Wironen, D. Ray, K. Johnson and J. Schmitt, "Greenhouse gas emissions in US beef production can be reduced by up to 30% with the adoption of selected mitigation measures," *Nature Food,* vol. 5, pp. 787-797, 2024.

[20] Argonne National Laboratory, "R&D GREET Model," 2024. [Online]. Available: https://greet.anl.gov. [Accessed 3 January 2025].

[21] GFLI, "Global Metrics for Sustainable Feed," 2024. [Online]. Available: https://globalfeedlca.org. [Accessed 5 October 2024].

[22] Sphera, "LCA for Experts Software," 2024. [Online]. Available: https://about.sphera.com/. [Accessed 3 January 2025].

[23] INRA-CIRAD-AFZ, "Feed Tables," 2024. [Online]. Available: https://www.feedtables.com. [Accessed 3 January 2025].

[24] K. Hales, C. Coppin, Z. Smith, Z. McDaniel, L. Tedeschi, N. Cole and M. Galyean, "Predicting metabolizable energy from digestible energy for growing and finishing beef cattle and relationships to the prediction of methane," *Journal of Animal Science,* vol. 100, no. 3, pp. 1-11, 2022.

[25] W. Weiss and A. Tebbe, "Estimating digestible energy values of feeds and diets and integrating those values into net energy systems," *Translational Animal Science,* vol. 3, no. 3, pp. 953-961, 2019.

[26] Food and Agriculture Organization, "Environmental performance of large ruminant supply chains: Guidelines for Assessment," 2016. [Online]. Available: https://openknowledge.fao.org/items/143aa579-884c-4c0e-8bbd-3a6cc7866636. [Accessed 3 January 2025].

[27] Agricultural Marketing Resource Center, "Productionand Revenue Trends in Corn Ethanol, DDGS, and Corn Distillers Oil," 2018. [Online]. Available: https://www.agmrc.org/renewable-energy/renewable-energy-climate-change-report/renewable-energy-climate-change-report/march-2018-report/production-and-revenue-trends-in-corn-ethanol-ddgs-and-corn-distillers-oil. [Accessed 3 January 2025].

[28] USDA, "Agricultural Census Fertilizre Use and Price," 2019.

[29] L. Moraes, A. Strathe, D. Fadel, D. Casper and E. Kebreab, "Prediction of enteric methane emissions from cattle," *Global Change Biology,* vol. 20, pp. 2140-2148, 2014.

[30] K. Reed, L. Moraes, D. Casper and E. Kebreab, "Predicting nitrogen excretion from cattle," *Journal of Dairy Science*, vol. 98, pp. 3025-3035, 2015.

[31] A. Leytem, S. Archibeque, N. Cole, S. Gunter, A. Hristov, K. Johnson, E. Kebreab, R. Kohn, W. Liao, C. Toureene and J. Tricarico, "Chapter 4: Quantifying greenhouse gas sources and sinks in animal production systems," in *Quantifying greenhouse gas fluxes in agriculture and forestry: Methods for entity-scale inventory*, 2024, pp. 4.1 - 4.64.

[32] US EPA, "Title 40, Chapter 1, Subchapter C, Part 98, Subpart JJ- Manure Management," 2024.

[33] US EPA, "Inventory of U.S. Greenhouse Gas Emissions and Sinks: 1990-2020: Annex 3," United States Environmental Protection Agency, 2022.

[34] J. Greene, J. Wallace, R. Williams, A. Leytem, B. Bock, M. McCully, S. Kaffka, A. Rotz and J. Quinn, "National Greenhouse Gas Emissions Reduction Potential from Adopting Anaerobic Digestion on Large-Scale Dairy Farms in the United States," *Environmental Science & Technology,* vol. 58, pp. 12409-12419, 2024.

[35] K. Hergoualc'h, H. Akiyama, M. Bernoux, N. Chirinda, A. Prado, A. Kasimir, J. MacDonald, S. Ogle, K. Regina and T. van der Weerden, "Chapter 11: N2O emissions from managed soils, and CO2 emissions from lime and urea application," in *2019 Refinement to the 2006 IPCC Guidelines for National Greenhouse Gas Inventories: Volume 4 - Agriculture, Forestry, and Other Land Use*, 2019, pp. 11.1 - 11.41.

[36] ASAE, "Manure Production and Characteristics," American Society of Agricultural Engineers, 2005.

[37] J. Ellis, E. Kebreab, N. Odongo, B. McBride, E. Okine and J. France, "Prediction of Methane Production from Dairy Beef Cattle," *Journal of Dairy Science,* vol. 90, pp. 3456-3467, 2007.

[38] G. Thoma, J. Popp, D. Shonnard, D. Nutter, M. Matlock, R. Ulrich, W. Kellogg, D. Kim, Z. Neiderman, N. Kemper, F. Adom and C. East, "Regional analysis of greenhouse gas emissions from USA dairy farms: A cradle to farm-gate assessment of the American dairy industry circa 2008," *International Dairy Journal,* vol. 31, pp. S29-S40, 2013.

[39] A. Rotz, R. L. A. Stout, G. Feyereisen, H. Waldrip, G. Thoma, M. Holly, D. Bjorneberg, J. Baker, P. Vadas and Kleinman, "Environmental assessment of United States dairy farms," *Journal of Cleaner Production,* vol. 315, no. 128153, pp. 1-13, 2021.

[40] M. Niu, E. Kebreab, A. Hristov, J. Oh, C. Arndt, A. Bannink, A. Bayat, A. Brito, T. Boland, D. Casper, L. Crompton, J. Dijkstra, M. Eugene, P. Garnswothry, M. Haque, A. Hellwing, P. Huhtanen, M. Kreuzer, B. Kuhla, P. Lund and Z. Yu, "Prediction of enteric methane production, yield and intensity in dairy cattle using an intercontinental database," *Global Change Biology,* pp. 3368-3389, 2018.
