# Supplementary material for: Correction to “Spatially Resolved Greenhouse Gas Emissions of U.S. Milk Production in 2020”
Source: Environ Sci Technol. 2025 Oct 21;59(43):23644–9. doi: 10.1021/acs.est.5c12673 (PMC12593345; doi:10.1021/acs.est.5c12673)
Supplement: Supplementary file 1 [file es5c12673_si_001.pdf]

Supplemental Information for:

Correction to “Spatially Resolved Greenhouse Gas Emissions of U.S. Milk Production in 2020”

Rylie Pelton[1,2], Juan Tricarico[3], Fabian Bernal[3], Mary Beth de Ondarza[4], Tim Kurt[3]

Affiliations:
[1]LEIF, LLC, St. Paul, MN 55113, USA
[2] University of Minnesota, Institute on the Environment, St. Paul, MN 55108, USA
[3] Dairy Management Inc., Rosemount, IL, 60018, USA
[4] Paradox Nutrition, LLC, Plattsburgh, NY, 12901, USA

Summary: 27 pages, 25 tables, 3 figures.

Table of Contents

Section S1. Growth and Performance 2
Section S2: Feed 4
Section S6. Results 15
Section S7. Sensitivity & Uncertainty Analysis 16

Section S1. Growth and Performance

Table S1. Lactation rates, milk fat and protein contents, and total annual FPCM produced in 2020 and 2007 (as indicated in brackets [ ]) by region.

Region	Kg milk/cow/day	Kg FPCM/cow/day	Milk crude protein %	Milk fat %	Total FPCM million tonnes
Northern Plains	29.0 [22.2]	30.1 [21.6]	**3.4% [3.2%]**	**4.2% [3.7%]**	**2.3M [1.4M]**
Upper Midwest	29.7 [23.5]	30.2 [22.9]	**3.4% [3.2%]**	**4.1% [3.7%]**	**22.2M [16.9M]**
Great Lakes	30.8 [25.0]	30.8 [24.2]	**3.4% [3.2%]**	**3.9% [3.7%]**	**9.7M [6.8M]**
Intermountain	31.2 [27.5]	31.2 [26.4]	**3.4% [3.3%]**	**3.9% [3.6%]**	**10.9M [7.1M]**
Southwest	30.4 [25.8]	30.7 [24.9]	**3.5% [3.3%]**	**4.0% [3.7%]**	**15.0M [9.8M]**
Pacific Northwest	28.9 [27.0]	29.7 [26.3]	**3.5% [3.3%]**	**4.1% [3.7%]**	**4.4M [3.4M]**
West	29.8 [27.1]	29.9 [26.2]	**3.4% [3.3%]**	**4.0% [3.7%]**	**19.2M [17.6M]**
Northeast	28.6 [23.7]	28.5 [23.1]	**3.3% [3.2%]**	**3.9% [3.7%]**	**11.6M [10.0M]**
New England	26.7 [22.9]	27.0 [22.4]	**3.4% [3.3%]**	**4.1% [3.8%]**	**1.9M [1.8M]**
Mid-Atlantic	24.9 [20.1]	24.9 [19.5]	**3.4% [3.1%]**	**3.9% [3.7%]**	**2.3M [2.8M]**
Mississippi Valley	17.8 [18.6]	17.8 [17.9]	**3.4% [3.3%]**	**3.9% [3.6%]**	**0.7M [1.3M]**
Southeast	26.0 [21.3]	25.1 [20.5]	**3.4% [3.3%]**	**3.7% [3.6%]**	**1.8M [1.5M]**

Note: True protein values used for constructing diets was converted to crude protein for FPCM calculation based on assumptions that true protein is lower than the percentage that is crude protein by about 0.19%.

Table S2. Estimated time spent in each lifecycle stage in 2020 and 2007.

Year	Days spent as calf[A]	Days spent as Heifer Replacement (15 months)[A]	Days spent as Heifer Replacement (24 months)[A]	Total days before first calving[B]	Days as Dry Cow[B,C]	Days in Lactation[B]	Total Lifetime Days/Cow[D]
2020	255	199	235	771	155	968	1894
2007	257	204	244	780	158	966	1904

[A] Based on daily average gain (kg/day) for each phase of the lifecycle (0.76 kg/day for calves, 0.82 for heifers) and typical animal mass (see Supplemental Table 4) associated with each life phase (e.g., [weight of heifer at 24 mo - weight of heifer at 15 mo] / [weight gain/day]).

[B] Average age at first calving, calving intervals, dry period length, based on Dairy Metrics Database in Capper and cady (2020) [1], and average number of lifetime lactations, see Supplemental Table 5.

[C] Total time spent in dry period over lifetime based on average dry period from Dairy Metrics Database [1] and average number of lifetime lactations, see Supplemental Table 5.

[D] Estimated by (1/replacement rate) x 12 month/yr x 30 days/month + age at first calving (days), see Supplemental Table 5.

Table S4. Typical liveweights (kg) by growth phase and cattle category.

Cattle Category	Liveweight kg
Dairy Cows	680 (2020)<br>635 (2007)
Heifer Replacements (15 months)	397
Heifer Replacements (24 months)	590
Weaned Calf	234
Calf birthweight	40
Bulls	959

Table S5. Growth and performance parameters for dairy production.

Key parameters	2020	2007
Dry period per lactation cycle (days)[A]	57	60
Calving Interval (days)[A]	414	426
Number of lifetime lactation[C]	2.71	2.64
Replacement rate (%)[B]	32.1%	32.0%
Average age at first calving (months)[A]	25.7	26.0
Cow/bull ratio[A]	25	25
Number breeding seasons for bulls[A]	4	4
Dairy cow mortality[A]	5.2%	5.3%
Heifer (weaning to breeding) mortality[A]	1.9%	1.9%
Calf (live birth to wean) mortality[A]	6.8%	6.8%

[A] Capper and Cady 2020 [1]
[B] USDA NASS (2024) [2]
[C] Estimated by ((1/replacement rate) x 12 months/yr x 30 days/months)/calving interval (days)

Table S6. Total annual dairy meat production in 2020 and 2007.

Category	2020	2007
Total commercial slaughter head[A]	32,785,700	34,264,000
Percent Steers[A]	49.3%	51.3%
Percent Heifers[A]	29.4%	30.3%
Estimated % Dairy Steers	19.9%	21%
Dairy Cows[A]	9.5%	7.4%
Total Culled Dairy Cows (tonnes liveweight)	2,119,170	1,610,141
Total Dairy Calves sold to feedlots (tonnes liveweight)	1,524,091	1,680,857
Tonnes FPCM	101,915,166	80,523,686
Milk allocation factor	81%	79%

[D] USDA NASS (2021) [3]

Section S2: Feed

Table S7. Formulated 2020 diets (DM basis) for lactating cows, dry cows, replacement heifers, and bulls in each of the 12 U.S. geographical regions.

Cattle Category	Diet Rations	Regions											
		NP	UM	GL	IM	SW	PNW	West	NE	NEG	MA	MV	SE
Lactating Dairy Cows	Corn silage [A]	37%	37%	37%	27%	23%	26%	24%	39%	40%	40%	31%	31%
	Alfalfa [A]	13%	18%	13%	21%	18%	18%	11%	14%	2%	6%	0%	0%
	Grass	1%	1%	2%	2%	6%	5%	6%	8%	20%	10%	9%	9%
	Corn Grain [A]	11%	15%	14%	17%	20%	19%	18%	14%	10%	15%	15%	15%
	Mineral Mix[C]	4%	3%	3%	3%	3%	4%	3%	3%	3%	4%	3%	3%
	Byproduct Blend	35%	25%	31%	30%	30%	29%	38%	22%	25%	26%	40%	40%
Dry Cows	Corn silage [A]	21%	34%	34%	21%	21%	17%	17%	41%	41%	10%	10%	10%
	Alfalfa [A]	18%	15%	15%	18%	18%	27%	27%	22%	22%	0%	0%	0%
	Grass	38%	28%	28%	38%	38%	32%	32%	22%	22%	63%	63%	63%
	Corn Grain [A]	3%	2%	2%	3%	3%	7%	7%	2%	2%	2%	2%	2%
	Mineral Mix[C]	2%	2%	2%	2%	2%	2%	2%	2%	2%	2%	2%	2%
	Byproduct Blend	19%	19%	19%	19%	19%	16%	16%	12%	12%	23%	23%	23%
Replacement Heifers	Corn silage [A]	17%	30%	30%	17%	17%	15%	15%	31%	31%	7%	23%	7%
	Alfalfa [A]	31%	26%	26%	31%	31%	19%	19%	36%	36%	4%	4%	4%
	Grass	26%	22%	22%	26%	26%	32%	32%	18%	18%	49%	49%	49%
	Corn Grain [A]	3%	4%	4%	3%	3%	5%	5%	3%	3%	8%	8%	8%
	Mineral Mix[C]	2%	3%	3%	2%	2%	3%	3%	3%	3%	3%	3%	3%
	Byproduct Blend	20%	15%	15%	20%	20%	25%	25%	10%	10%	30%	30%	30%
Bulls	Corn silage [A]	17%	30%	30%	17%	17%	15%	15%	31%	31%	23%	23%	23%
	Alfalfa [A]	31%	26%	26%	31%	31%	19%	19%	36%	36%	4%	4%	4%
	Grass	26%	22%	22%	26%	26%	32%	32%	18%	18%	49%	49%	49%
	Corn Grain [A]	3%	4%	4%	3%	3%	5%	5%	3%	3%	8%	8%	8%
	Mineral Mix[C]	2%	3%	3%	2%	2%	3%	3%	3%	3%	3%	3%	3%
	Byproduct Blend	20%	15%	15%	20%	20%	25%	25%	10%	10%	30%	30%	30%

Midwest byproduct blend = Northern Plains (NP), Upper Midwest (UM), Great Lakes (GL); West byproduct blend = Intermountain (IM), Southwest (SW), Pacific Northwest (PNW), West; Northeast byproduct blend = Northeast (NE), New England (NEG); South byproduct blend = Mid-Atlantic (MA), Mississippi Valley (MV), Southeast (SE).

[A] GHG emission estimates based on FoodS^3 models considering county scale commodity flows and emission factors [4, 5]

[B] GHG emissions estimated based on FAF5 commodity flows, supply and demand distributions; with spatially estimated emissions at state scale [6].

[C] GHG emissions estimated based on GFLI [7], literature, or other LCA databases reflecting national averages [8].

Table S8. Formulated 2007 diets (DM basis) for lactating cows, dry cows, replacement heifers, and bulls in each of the 12 U.S. geographical regions.

Cattle Category	Diet Rations	Regions											
		NP	UM	GL	IM	SW	PNW	West	NE	NEG	MA	MV	SE
Lactating Dairy Cows	Corn silage [A]	37%	37%	37%	27%	23%	26%	24%	39%	40%	40%	31%	31%
	Alfalfa [A]	13%	18%	13%	21%	18%	18%	11%	14%	2%	6%	0%	0%
	Grass	1%	1%	2%	2%	6%	5%	6%	8%	20%	10%	9%	9%
	Corn Grain [A]	11%	15%	14%	17%	20%	19%	18%	14%	10%	15%	15%	15%
	Mineral Mix[C]	4%	3%	3%	3%	3%	3%	3%	3%	3%	4%	3%	3%
	Byproduct Blend	35%	25%	31%	30%	30%	29%	38%	22%	25%	26%	40%	40%
Dry Cows	Corn silage [A]	21%	34%	34%	21%	21%	17%	17%	41%	41%	10%	10%	10%
	Alfalfa [A]	18%	15%	15%	18%	18%	27%	27%	22%	22%	0%	0%	0%
	Grass	38%	28%	28%	38%	38%	32%	32%	22%	22%	63%	63%	63%
	Corn Grain [A]	3%	2%	2%	3%	3%	7%	7%	2%	2%	2%	2%	2%
	Mineral Mix[C]	2%	2%	2%	2%	2%	2%	2%	2%	2%	2%	2%	2%
	Byproduct Blend	19%	19%	19%	19%	19%	16%	16%	12%	12%	23%	23%	23%
Replacement Heifers	Corn silage [A]	17%	30%	30%	17%	17%	15%	15%	31%	31%	7%	7%	7%
	Alfalfa [A]	31%	26%	26%	31%	31%	19%	19%	36%	36%	4%	4%	4%
	Grass	26%	22%	22%	26%	26%	32%	32%	18%	18%	49%	49%	49%
	Corn Grain [A]	3%	4%	4%	3%	3%	5%	5%	3%	3%	8%	8%	8%
	Mineral Mix[C]	2%	3%	3%	2%	2%	3%	3%	3%	3%	3%	3%	3%
	Byproduct Blend	20%	15%	15%	20%	20%	25%	25%	10%	10%	30%	30%	30%
Bulls	Corn silage [A]	17%	30%	30%	17%	17%	15%	15%	31%	31%	7%	7%	7%
	Alfalfa [A]	31%	26%	26%	31%	31%	19%	19%	36%	36%	4%	4%	4%
	Grass	26%	22%	22%	26%	26%	32%	32%	18%	18%	49%	49%	49%
	Corn Grain [A]	3%	4%	4%	3%	3%	5%	5%	3%	3%	8%	8%	8%
	Mineral Mix[C]	2%	3%	3%	2%	2%	3%	3%	3%	3%	3%	3%	3%
	Byproduct Blend	20%	15%	15%	20%	20%	25%	25%	10%	10%	30%	30%	30%

Midwest byproduct blend = Northern Plains (NP), Upper Midwest (UM), Great Lakes (GL); West byproduct blend = Intermountain (IM), Southwest (SW), Pacific Northwest (PNW), West; Northeast byproduct blend = Northeast (NE), New England (NEG); South byproduct blend = Mid-Atlantic (MA), Mississippi Valley (MV), Southeast (SE).

[A] GHG emission estimates based on FoodS^3 models considering county scale commodity flows and emission factors [4, 5]

[B] GHG emissions estimated based on FAF5 commodity flows, supply and demand distributions; with spatially estimated emissions at state scale [6].

[C] GHG emissions estimated based on GFLI [7], literature, or other LCA databases reflecting national averages [8].

Table S9. Percentage of each feed byproduct (DM basis) within byproduct blends used in diets for lactating cows, dry cows, replacement heifers and bulls in each of the 12 US geographical regions shown in Tables S7 and S8.

Byproduct	Midwest	West	Northeast	South	% Dry Matter
Almond Hulls [A]	0.9%	9.2%	0.0%	0.0%	87%
Bakery Waste [A]	1.6%	0.6%	5.4%	0.0%	95%
Beet Pulp [B]	1.0%	0.7%	2.8%	0.0%	91%
Bloodmeal [B]	1.3%	1.1%	2.1%	3.8%	90%
Brewer's Grain Dry [A]	0.0%	0.3%	0.8%	0.0%	93%
Brewer's Grain Wet [A]	1.1%	0.9%	1.3%	1.8%	25%
Candy [B]	0.0%	0.1%	0.3%	0.0%	89%
Canola Meal [A]	9.1%	22.6%	14.4%	1.7%	88%
Canola Meal Treated[A]	0.0%	0.1%	3.5%	0.0%	95%
Cereal [A]	20.6%	0.5%	0.0%	0.0%	93%
Chocolate [B]	0.0%	0.1%	0.0%	0.0%	89%
Citrus Pulp Dry [A]	1.5%	0.2%	1.2%	2.4%	89%
Citrus Pulp Wet [A]	0.0%	1.6%	0.0%	0.0%	19%
Corn cannery waste [A]	6.6%	0.3%	0.0%	0.0%	22%
DDGS[A]	8.2%	14.7%	6.4%	12.2%	89%
DDG wet [A]	0.6%	5.3%	0.0%	0.0%	32%
Corn germ [B]	0.0%	1.4%	0.0%	0.0%	93%
Corn gluten feed dry [A]	2.5%	2.8%	3.9%	19.7%	90%
Corn gluten feed wet [A]	1.7%	0.2%	0.2%	0.0%	41%
Corn gluten meal [A]	0.3%	0.1%	1.1%	0.0%	92%
Corn starch [B]	0.1%	0.0%	2.5%	0.0%	92%
Corn steep liquor [B]	0.1%	0.4%	0.0%	0.0%	45%
Cottonseed Whole [A]	7.5%	13.4%	3.1%	16.7%	92%
Cottonseed Hulls [B]	0.4%	0.1%	0.0%	0.0%	92%
Cottonseed meal [B]	0.0%	0.4%	0.1%	1.7%	92%
Animal fat [B]	0.2%	0.4%	0.3%	0.0%	99%
Palm fat [B]	1.6%	0.7%	2.5%	0.4%	99%
Vegetable fat [B]	0.0%	0.0%	0.1%	0.0%	99%
Feather meal [B]	0.0%	0.1%	0.1%	0.0%	93%
Fishmeal [B]	0.0%	0.0%	0.0%	0.0%	90%
Hominy [B]	1.2%	0.6%	0.1%	1.2%	88%
Linseed meal [B]	0.0%	1.0%	0.0%	0.0%	88%
Malt sprouts [A]	0.8%	0.5%	0.2%	0.0%	93%
Meat & Bone meal [B]	0.0%	0.0%	0.0%	0.0%	94%
Meat & Bonemeal (MBM)[B]	0.7%	0.0%	0.0%	0.0%	95%
Molasses (beet)[B]	1.8%	0.4%	0.1%	0.0%	75%
Molasses (cane)[A]	2.7%	0.9%	3.9%	0.9%	73%
Oat Hulls [A]	1.1%	0.0%	0.2%	0.0%	93%
Oat Mill Feed [B]	0.0%	0.0%	0.0%	0.0%	90%
Palm Kernel meal [B]	0.0%	0.1%	0.0%	0.0%	92%
Peanut Hulls [B]	0.1%	0.0%	0.0%	0.0%	93%
Peanut meal [B]	0.0%	0.1%	0.0%	0.0%	92%
Potato [B]	0.6%	0.6%	0.0%	0.0%	94%
Rice Bran [B]	0.0%	0.3%	0.0%	0.0%	91%
Rice Hulls [B]	0.0%	0.0%	0.0%	0.0%	93%
Rice Mill Feed [B]	0.0%	0.1%	0.0%	0.0%	90%
Safflower meal [B]	0.0%	0.2%	0.0%	0.0%	94%
Soybean Hulls [A]	1.8%	2.4%	4.6%	4.0%	91%
Soybean Meal [A]	11.9%	5.2%	20.6%	22.7%	90%
Soybean Meal Treated[A]	5.1%	2.8%	6.8%	2.4%	90%
Sugar [B]	0.0%	0.0%	0.5%	0.0%	98%
Sunflower meal [B]	0.0%	0.2%	0.0%	0.0%	93%
Wheat Bran [B]	0.0%	0.4%	0.1%	0.0%	89%
Wheat Distillers Dry [B]	0.0%	0.6%	0.0%	0.0%	92%
Wheat Distillers Wet [B]	0.0%	0.4%	0.0%	0.0%	32%
Wheat flour [B]	0.0%	0.1%	0.0%	0.0%	95%
Wheat Midds [B]	4.5%	0.8%	5.8%	0.0%	89%
Wheat Mill Run [B]	0.2%	1.6%	0.0%	0.0%	95%

Wheat Red dog flour [B]	0.0%	0.1%	2.0%	0.0%	89%
Wheat shorts [B]	0.0%	0.0%	0.0%	0.0%	89%
Whey Dry [A]	0.0%	0.0%	0.0%	0.0%	95%
Whey Liquid [B]	0.2%	0.3%	0.9%	0.0%	7%
Whey condensed [A]	0.3%	1.9%	1.8%	8.3%	20%

Midwest = Northern Plains (NP), Upper Midwest (UM), Great Lakes (GL); West = Intermountain (IM), Southwest (SW), Pacific Northwest (PNW), West; Northeast = Northeast (NE), New England (NEG); South = Mid-Atlantic (MA), Mississippi Valley (MV), Southeast (SE).

[A] See Table S17

[B] Emission factors based on GFLI [7], literature, or other LCA databases reflecting national averages [8].

Table S10. Nutrient composition of 2020 lactating dairy diets for each of the 12 U.S. geographical regions.

Dietary parameters	Regions											
	NP	UM	GL	IM	SW	PNW	West	NE	NEG	MA	MV	SE
Dry Matter Intake (kg/day)	24.1	23.8	24.1	24.7	24.2	23.9	24.8	23.3	22.3	21.6	20.0	22.9
% Dry matter[A]	59.8%	57.4%	59.4%	71.7%	71.8%	71.2%	70.8%	56.1%	56.1%	69.4%	71.7%	71.7%
Organic Matter[A] (OM) % DM	91.4%	91.8%	92.0%	91.1%	91.2%	91.1%	91.3%	91.2%	91.2%	91.6%	91.9%	91.9%
Crude protein (CP)[A] % DM	15.8%	16.2%	15.9%	16.3%	16.4%	16.1%	17.2%	16.3%	15.6%	15.7%	18.1%	18.1%
Ether Extract (EE)[A] % DM	4.2%	4.0%	4.2%	4.5%	4.6%	4.5%	5.1%	3.8%	3.9%	4.1%	4.7%	4.7%
Neutral Detergent Fiber (NDF)[A] % DM	31.5%	32.9%	34.0%	32.7%	32.2%	32.2%	32.6%	33.9%	35.9%	37.2%	35.3%	35.3%
Acid Detergent Fiber (ADF)[A] % DM	19.0%	20.3%	20.7%	21.3%	20.9%	20.8%	20.6%	21.1%	21.6%	22.7%	20.9%	20.9%
Ash[A] % DM	8.6%	8.2%	8.0%	8.9%	8.8%	8.9%	8.7%	8.8%	8.8%	8.5%	8.1%	8.1%
Forage % DM	51.0%	56.2%	51.3%	49.7%	46.8%	48.0%	41.1%	60.5%	61.6%	55.4%	40.8%	40.8%
Metabolizable energy (ME)[A] MJ/kg DM	10.5	10.6	10.5	10.2	10.2	10.2	10.1	10.4	10.3	10.3	10.3	10.2
Gross Energy (GE)[A] MJ/kg DM	17.7	17.5	17.7	17.9	17.9	17.8	18.1	17.5	17.5	17.6	17.9	17.9
Digestible Energy (DE)[B] MJ/kg DM	12.8	12.6	12.7	12.6	12.6	12.6	13.0	12.4	12.4	12.8	13.1	13.1

Northern Plains (NP), Upper Midwest (UM), Great Lakes (GL), Intermountain (IM), Southwest (SW), Pacific Northwest (PNW), West, Northeast (NE), New England (NEG), Mid-Atlantic (MA), Mississippi Valley (MV), Southeast (SE).

[A] Based on nutrient composition of feed ingredients from Higgs et al 2015 [9] and Van Amburgh et al 2015 [10]

[B] Based on Galyean et al 2016 equation 2 [11]

Table S11. Nutrient composition of 2007 lactating dairy diets for each of the 12 U.S. geographical regions.

Dietary parameters	Regions											
	NP	UM	GL	IM	SW	PNW	West	NE	NEG	MA	MV	SE
Dry Matter Intake (kg/day)	21.4	22.2	22.8	22.8	22.6	23.3	23.3	21.8	21.6	20.9	19.5	21.2
% Dry matter[A]	59.8%	57.4%	59.4%	71.7%	71.8%	71.1%	70.8%	56.1%	56.0%	69.4%	71.7%	71.7%
Organic Matter[A] (OM) % DM	91.9%	92.4%	92.5%	91.4%	91.5%	91.5%	91.5%	91.9%	91.8%	92.1%	91.8%	92.0%
Crude protein (CP)[A] % DM	17.3%	17.1%	17.2%	17.3%	17.2%	17.2%	17.4%	17.2%	17.2%	17.2%	18.0%	18.1%
Ether Extract (EE)[A] % DM	4.1%	4.1%	4.2%	4.6%	4.6%	4.6%	5.0%	3.7%	3.9%	4.2%	4.7%	4.7%
Neutral Detergent Fiber (NDF)[A] % DM	35.2%	34.3%	34.3%	35.1%	34.1%	34.3%	34.8%	36.3%	40.5%	36.9%	35.3%	35.4%
Acid Detergent Fiber (ADF)[A] % DM	21.5%	21.3%	20.9%	22.9%	22.2%	22.2%	22.0%	22.7%	24.6%	22.4%	20.9%	20.9%
Ash[A] % DM	8.1%	7.7%	7.5%	8.6%	8.5%	8.5%	8.5%	8.1%	8.2%	7.9%	8.2%	8.0%
Forage % DM	51.0%	56.2%	51.3%	49.7%	46.8%	48.0%	41.1%	60.5%	61.6%	55.4%	40.8%	40.7%
Metabolizable energy (ME)[A] MJ/kg DM	9.9	10.0	9.9	9.8	9.7	9.8	9.7	9.7	9.5	9.8	10.2	10.2
Gross Energy (GE)[A] MJ/kg DM	17.7	17.5	17.7	17.9	17.9	17.9	18.1	17.5	17.5	17.6	17.9	17.9
Digestible Energy (DE)[B] MJ/kg DM	12.8	12.6	12.7	12.6	12.7	12.6	13.0	12.4	12.4	12.8	13.1	13.1

Northern Plains (NP), Upper Midwest (UM), Great Lakes (GL), Intermountain (IM), Southwest (SW), Pacific Northwest (PNW), West, Northeast (NE), New England (NEG), Mid-Atlantic (MA), Mississippi Valley (MV), Southeast (SE).
[A] Based on nutrient composition of feed ingredients from Higgs et al 2015 [9] and Van Amburgh et al 2015 [10]
[B] Based on Galyean et al 2016 equation 2 [11]

Table S12. Nutrient composition of 2020 dry cow diets for each of the 12 U.S. geographical regions.

Dietary parameters	Regions											
	NP	UM	GL	IM	SW	PNW	West	NE	NEG	MA	MV	SE
Dry Matter Intake (kg/day)	13.3	13.3	13.3	13.3	13.3	13.3	13.3	13.3	13.3	13.3	13.3	13.3
% Dry matter[A]	68.5%	59.6%	59.6%	71.2%	71.2%	72.3%	72.3%	46.6%	46.6%	84.5%	83.9%	83.9%
Organic Matter[A] (OM) % DM	91.2%	91.8%	91.8%	91.2%	91.2%	91.1%	91.1%	91.7%	91.7%	91.0%	91.1%	91.1%
Crude protein (CP)[A] % DM	12.8%	12.9%	12.9%	13.4%	13.4%	13.6%	13.6%	13.2%	13.2%	13.3%	13.0%	13.0%
Ether Extract (EE)[A] % DM	3.6%	3.7%	3.7%	4.0%	4.0%	3.9%	3.9%	3.3%	3.3%	3.5%	3.8%	3.8%
Neutral Detergent Fiber (NDF)[A] % DM	51.2%	49.2%	49.2%	52.0%	52.0%	49.3%	49.3%	48.8%	48.8%	55.9%	58.0%	58.0%
Acid Detergent Fiber (ADF)[A] % DM	34.4%	30.9%	30.9%	33.5%	33.5%	32.5%	32.5%	31.5%	31.5%	34.1%	35.4%	35.4%
Ash[A] % DM	8.8%	8.2%	8.2%	8.8%	8.8%	8.9%	8.9%	8.3%	8.3%	9.0%	8.9%	8.9%
Forage % DM	76.8%	77.1%	77.1%	76.8%	76.8%	75.8%	75.8%	84.3%	84.3%	72.9%	72.9%	72.9%
Metabolizable energy (ME)[A] MJ/kg DM	9.0	9.1	9.1	8.8	8.8	8.8	8.8	8.9	8.9	8.7	8.5	8.5
Gross Energy (GE)[A] MJ/kg DM	17.2	17.3	17.3	17.3	17.3	17.3	17.3	17.2	17.2	17.2	17.2	17.2
Digestible Energy (DE)[B] MJ/kg DM	11.5	11.6	11.6	11.5	11.5	11.4	11.4	11.4	11.4	11.9	11.7	11.7

Northern Plains (NP), Upper Midwest (UM), Great Lakes (GL), Intermountain (IM), Southwest (SW), Pacific Northwest (PNW), West, Northeast (NE), New England (NEG), Mid-Atlantic (MA), Mississippi Valley (MV), Southeast (SE).

[A] Based on nutrient composition of feed ingredients from Higgs et al 2015 [9] and Van Amburgh et al 2015 [10]

[B] Based on Galyean et al 2016 equation 2 [11]

Table S13. Nutrient composition of 2007 dry cow diets for each of the 12 U.S. geographical regions.

Dietary parameters	Regions											
	NP	UM	GL	IM	SW	PNW	West	NE	NEG	MA	MV	SE
Dry Matter Intake (kg/day)	13.2	13.2	13.2	13.2	13.2	13.2	13.2	13.2	13.2	13.2	13.2	13.2
% Dry matter[A]	68.5%	59.6%	59.6%	71.2%	71.2%	72.3%	72.3%	46.6%	46.6%	84.5%	83.9%	83.9%
Organic Matter[A] (OM) % DM	91.6%	91.7%	91.7%	91.1%	91.1%	90.8%	90.8%	91.1%	91.1%	91.1%	91.6%	91.6%
Crude protein (CP)[A] % DM	14.0%	13.7%	13.7%	14.6%	14.6%	14.6%	14.6%	13.9%	13.9%	13.7%	14.4%	14.4%
Ether Extract (EE)[A] % DM	3.6%	3.7%	3.7%	4.0%	4.0%	3.9%	3.9%	3.3%	3.3%	3.5%	3.8%	3.8%
Neutral Detergent Fiber (NDF)[A] % DM	49.3%	47.8%	47.8%	50.1%	50.1%	47.7%	47.7%	47.7%	47.7%	55.2%	55.6%	55.6%
Acid Detergent Fiber (ADF)[A] % DM	31.3%	30.1%	30.1%	32.4%	32.4%	31.6%	31.6%	30.9%	30.9%	33.7%	34.0%	34.0%
Ash[A] % DM	8.4%	8.3%	8.3%	9.0%	9.0%	9.2%	9.2%	8.9%	8.9%	8.9%	8.4%	8.4%
Forage % DM	76.8%	77.1%	77.1%	76.8%	76.8%	75.8%	75.8%	84.3%	84.3%	72.9%	72.9%	72.9%
Metabolizable energy (ME)[A] MJ/kg DM	9.2	9.2	9.2	9.0	9.0	8.9	8.9	8.6	8.6	8.8	8.8	8.8
Gross Energy (GE)[A] MJ/kg DM	17.2	17.3	17.3	17.3	17.3	17.3	17.3	17.2	17.2	17.2	17.2	17.2
Digestible Energy (DE)[B] MJ/kg DM	11.5	11.6	11.6	11.5	11.5	11.4	11.4	11.4	11.4	11.9	11.7	11.7

Northern Plains (NP), Upper Midwest (UM), Great Lakes (GL), Intermountain (IM), Southwest (SW), Pacific Northwest (PNW), West, Northeast (NE), New England (NEG), Mid-Atlantic (MA), Mississippi Valley (MV), Southeast (SE).

[A] Based on nutrient composition of feed ingredients from Higgs et al 2015 [9] and Van Amburgh et al 2015 [10]

[B] Based on Galyean et al 2016 equation 2 [11]

Table S14. Nutrient composition of 2020 replacement heifer diets for each of the 12 U.S. geographical regions.

Dietary parameters	Regions											
	NP	UM	GL	IM	SW	PNW	West	NE	NEG	MA	MV	SE
Dry Matter Intake (kg/day)	8.5	8.5	8.5	8.5	8.5	8.5	8.5	8.5	8.5	8.5	8.5	8.5
% Dry matter[A]	80.2%	59.5%	59.5%	80.0%	80.0%	80.9%	80.9%	45.8%	45.8%	85.8%	84.7%	84.7%
Organic Matter[A] (OM) % DM	90%	91%	91%	90%	90%	90%	90%	90%	90%	90%	90%	90%
Crude protein (CP)[A] % DM	14.3%	13.2%	13.2%	15.0%	15.0%	14.9%	14.9%	13.8%	13.8%	15.3%	15.3%	15.3%
Ether Extract (EE)[A] % DM	3.4%	3.5%	3.5%	3.9%	3.9%	4.2%	4.2%	3.5%	3.5%	3.6%	4.2%	4.2%
Neutral Detergent Fiber (NDF)[A] % DM	47.5%	47.1%	47.1%	48.3%	48.3%	47.9%	47.9%	46.9%	46.9%	48.3%	49.3%	49.3%
Acid Detergent Fiber (ADF)[A] % DM	31.2%	30.5%	30.5%	32.2%	32.2%	31.0%	31.0%	31.5%	31.5%	29.5%	30.2%	30.2%
Ash[A] % DM	9.5%	9.3%	9.3%	9.6%	9.6%	9.8%	9.8%	9.9%	9.9%	9.7%	9.6%	9.6%
Forage % DM	74.7%	78.2%	78.2%	74.7%	74.7%	66.3%	66.3%	84.6%	84.6%	59.9%	59.9%	59.9%
Metabolizable energy (ME)[A] MJ/kg DM	8.8	8.9	8.9	8.7	8.7	8.8	8.8	8.6	8.6	8.9	8.9	8.9
Gross Energy (GE)[A] MJ/kg DM	17.1	17.0	17.0	17.4	17.4	17.5	17.5	16.8	16.7	17.5	17.3	17.3
Digestible Energy (DE)[B] MJ/kg DM	11.5	11.7	11.5	11.1	11.1	11.4	12.1	11.0	11.0	13.0	11.7	11.7

Northern Plains (NP), Upper Midwest (UM), Great Lakes (GL), Intermountain (IM), Southwest (SW), Pacific Northwest (PNW), West, Northeast (NE), New England (NEG), Mid-Atlantic (MA), Mississippi Valley (MV), Southeast (SE).

[A] Based on nutrient composition of feed ingredients from Higgs et al 2015 [9] and Van Amburgh et al 2015 [10]

[B] Based on Galyean et al 2016 equation 2 [11]

Table S15. Nutrient composition of 2007 replacement heifer diets for each of the 12 U.S. geographical regions.

Dietary parameters	Regions											
	NP	UM	GL	IM	SW	PNW	West	NE	NEG	MA	MV	SE
Dry Matter Intake (kg/day)	7.9	7.9	7.9	7.9	7.9	7.9	7.9	7.9	7.9	7.9	7.9	7.9
% Dry matter[A]	80.2%	59.5%	59.5%	80.0%	80.0%	80.9%	80.9%	45.8%	45.8%	85.8%	84.7%	84.7%
Organic Matter[A] (OM) % DM	90%	91%	91%	90%	90%	90%	90%	90%	90%	90%	90%	90%
Crude protein (CP)[A] % DM	14.1%	12.7%	12.7%	14.7%	14.7%	14.7%	14.7%	13.3%	13.3%	15.2%	15.2%	15.2%
Ether Extract (EE)[A] % DM	3.4%	3.5%	3.5%	3.9%	3.9%	4.2%	4.2%	3.5%	3.5%	3.6%	4.2%	4.2%
Neutral Detergent Fiber (NDF)[A] % DM	46.1%	44.6%	44.6%	46.9%	46.9%	46.7%	46.7%	44.5%	44.5%	47.8%	48.8%	48.8%
Acid Detergent Fiber (ADF)[A] % DM	30.3%	28.8%	28.8%	31.3%	31.3%	30.2%	30.2%	29.8%	29.8%	29.1%	29.9%	29.9%
Ash[A] % DM	9.6%	9.4%	9.4%	9.7%	9.7%	9.9%	9.9%	9.9%	9.9%	9.7%	9.7%	9.7%
Forage % DM	74.7%	78.2%	78.2%	74.7%	74.7%	66.3%	66.3%	84.6%	84.6%	59.9%	59.9%	59.9%
Metabolizable energy (ME)[A] MJ/kg DM	9.1	9.3	9.3	8.9	8.9	9.0	9.0	9.1	9.1	9.1	9.0	9.0
Gross Energy (GE)[A] MJ/kg DM	17.1	17.0	17.0	17.4	17.4	17.5	17.5	16.8	16.7	17.5	17.3	17.3
Digestible Energy (DE)[B] MJ/kg DM	11.5	11.7	11.5	11.1	11.1	11.4	12.1	11.0	11.0	13.0	11.7	11.7

Northern Plains (NP), Upper Midwest (UM), Great Lakes (GL), Intermountain (IM), Southwest (SW), Pacific Northwest (PNW), West, Northeast (NE), New England (NEG), Mid-Atlantic (MA), Mississippi Valley (MV), Southeast (SE).

[A] Based on nutrient composition of feed ingredients from Higgs et al 2015 [9] and Van Amburgh et al 2015 [10]

[B] Based on Galyean et al 2016 equation 2 [11]

Table S16. Nutrient composition of 2020 bull diets for each of the 12 U.S. geographical regions.

Dietary parameters	Regions											
	NP	UM	GL	IM	SW	PNW	West	NE	NEG	MA	MV	SE
Dry Matter Intake (kg/day)	7.3	7.3	7.3	7.3	7.3	6.6	6.6	6.6	6.6	6.6	6.6	6.6
% Dry matter[A]	80.2%	59.5%	59.5%	80.0%	80.0%	80.9%	80.9%	45.8%	45.8%	85.8%	84.7%	84.7%
Organic Matter[A] (OM) % DM	90%	91%	91%	90%	90%	90%	90%	90%	90%	90%	90%	90%
Crude protein (CP)[A] % DM	14.3%	13.2%	13.2%	15.0%	15.0%	14.9%	14.9%	13.8%	13.8%	15.3%	15.3%	15.3%
Ether Extract (EE)[A] % DM	3.4%	3.5%	3.5%	3.9%	3.9%	4.2%	4.2%	3.5%	3.5%	3.6%	4.2%	4.2%
Neutral Detergent Fiber (NDF)[A] % DM	47.5%	47.1%	47.1%	48.3%	48.3%	47.9%	47.9%	46.9%	46.9%	48.3%	49.3%	49.3%
Acid Detergent Fiber (ADF)[A] % DM	31.2%	30.5%	30.5%	32.2%	32.2%	31.0%	31.0%	31.5%	31.5%	29.5%	30.2%	30.2%
Ash[A] % DM	9.5%	9.3%	9.3%	9.6%	9.6%	9.8%	9.8%	9.9%	9.9%	9.7%	9.6%	9.6%
Forage % DM	74.7%	78.2%	78.2%	74.7%	74.7%	66.3%	66.3%	84.6%	84.6%	59.9%	59.9%	59.9%
Metabolizable energy (ME)[A] MJ/kg DM	8.8	8.9	8.9	8.7	8.7	8.8	8.8	8.6	8.6	8.9	8.9	8.9
Gross Energy (GE)[A] MJ/kg DM	17.1	17.2	17.2	17.5	17.5	17.5	17.5	17.1	17.1	17.3	17.3	17.3
Digestible Energy (DE)[B] MJ/kg DM	11.3	11.4	11.3	11.0	11.0	11.3	12.0	10.7	10.8	12.9	11.6	11.6

Northern Plains (NP), Upper Midwest (UM), Great Lakes (GL), Intermountain (IM), Southwest (SW), Pacific Northwest (PNW), West, Northeast (NE), New England (NEG), Mid-Atlantic (MA), Mississippi Valley (MV), Southeast (SE).

[A] Based on nutrient composition of feed ingredients from Higgs et al 2015 [9] and Van Amburgh et al 2015 [10]

[B] Based on Galyean et al 2016 equation 2 [11]

Table S17. Feed consumption-based emission factors (as-fed basis) and land use change (in brackets [ ]) emission factors (kgCO_2e/tonne) by dairy consumption region in 2020.

Feed Emission Factors [E]	Regions											
	NP	UM	GL	IM	SW	PNW	West	NE	NEG	MA	MV	SE
Corn[A,B]	701 [272]	433 [102]	432 [94]	507 [46]	602 [94]	507 [143]	536 [28]	1014 [667]	1331 [943]	963 [613]	650 [222]	816 [414]
Corn Silage[A,B]	254 [6]	168 [4]	156 [3]	83 [1]	108 [3]	95 [44]	87 [0.5]	163 [33]	132 [61]	182 [27]	185 [7]	159 [35]
Alfalfa[A,B]	246 [690]	179 [578]	227 [887]	134 [124]	134 [305]	129 [129]	104 [216]	165 [558]	129 [198]	168 [463]	164 [335]	127 [345]
Soymeal[A,B]	1114 [279]	1020 [134]	1101 [84]	1062 [119]	1224 [287]	1011 [127]	1082 [138]	1029 [90]	1024 [92]	1048 [208]	1048 [287]	1070 [392]
Soy Hulls[A,B]	229 [102]	245 [110]	250 [114]	254 [117]	249 [112]	270 [126]	253 [117]	242 [108]	240 [107]	249 [114]	246 [110]	253 [116]
DDGS[A,B]	998 [125]	1012 [32]	1015 [31]	965 [99]	989 [46]	917 [105]	989 [66]	925 [111]	733 [206]	989 [16]	1070 [48]	932 [65]
WGS[B,D]	259 [9]	258 [9]	258 [8]	256 [9]	256 [9]	254 [10]	257 [9]	258 [9]	258 [9]	258 [8]	258 [9]	258 [7]
Canola Meal[C]	331 [214]	331 [206]	332 [213]	332 [210]	333 [207]	331 [213]	333 [215]	333 [208]	345 [219]	335 [152]	332 [136]	335 [169]
Corn Gluten Feed Meal (Wet)[B,D]	123 [3]	122 [3]	122 [3]	119 [3]	120 [3]	117 [4]	122 [3]	122 [3]	122 [2]	122 [3]	122 [3]	122 [3]
Corn Gluten Feed Dry[B,D]	353 [7]	350 [7]	350 [7]	343 [8]	345 [8]	338 [9]	350 [7]	350 [7]	349 [5]	350 [7]	350 [7]	350 [7]
Cottonseed[C,D]	302 [6]	291 [6]	293 [6]	296 [5]	292 [7]	296 [6.7]	294 [7]	295 [7]	297 [5]	291 [6]	290 [6]	291 [6]
Cereal/Bakery[C,D]	46	45	45	45	45	43	45	45	45	45	45	45
Molasses Cane[C,D]	386 [304]	467 [302]	467 [302]	470 [302]	468 [300]	467 [302]	468 [302]	468 [302]	569 [298]	468 [302]	468 [302]	468 [302]
Brewers Grain Wet[C,D]	11 [0.8]	12 [1.0]	12 [1.0]	11 [0.9]	11 [0.9]	11 [0.9]	11 [0.9]	12 [1.0]	12 [1.0]	12 [1.0]	11 [0.9]	11 [0.9]
Brewers Grain Dry[C,D]	420 [5]	426 [6]	426 [6]	420 [5]	422 [5]	422 [6]	424 [6]	426 [6]	427 [6]	426 [6]	423 [6]	425 [6]
Citrus Pulp Wet[C,D]	119 [.01]	119 [.01]	121 [.01]	120 [.01]	121 [.01]	119 [.01]	122 [.01]	122 [.01]	119 [.01]	122 [.01]	123 [.01]	122 [.01]
Corn Cannery Residue[C,D]	196	189	189	184	187	187	189	190	192	190	190	190
Almond Hulls[C,D]	79 [64]	79 [64]	79 [64]	79 [64]	79 [64]	79 [64]	79 [64]	79 [64]	79 [64]	79 [64]	79 [64]	79 [64]
Malt Sprouts[C,D]	411 [34]	452 [39]	452 [38]	416 [36]	426 [37]	427 [37]	442 [38]	452 [39]	459 [39]	456 [39]	431 [37]	449 [38]
Whey Condensed[C,D]	534 [33]	532 [33]	532 [33]	532 [33]	532 [33]	532 [33]	532 [33]	532 [33]	536 [33]	532 [33]	532 [33]	532 [33]
Whey Dry[C,D]	2853 [203]	2850 [203]	2850 [203]	2850 [203]	2850 [203]	2849 [203]	2850 [203]	2850 [203]	2858 [203]	2850 [203]	2850 [203]	2850 [203]
Liquid Whey[C,D]	24 [3]	24 [3]	24 [3]	24 [3]	24 [3]	24 [3]	24 [3]	24 [3]	24 [3]	24 [3]	24 [3]	24 [3]

[A] Uses AR6 for direct and indirect N_2O, and AR5 for all upstream manufacturing. Note that upstream manufacturing emissions are primarily driven by CO2 emissions which do not change between AR5 and AR6.
[B] Based on county-scale LCA of crop production [5, 4] with supply chain models from FoodS[3].
[C] Based on national LCA of crop production adjusted for state-specific yields with supply chain models based on FAF5 [6].
[D] Processing emissions estimated based on DMI-commissioned study. Dairy products allocated based on economic allocation.
[E] Transport emissions from feed sourcing included separately. Total emissions intensity from feed purchases (excluding transport) based on addition of unbracketed and bracketed values.

Section S6. Results

Table S29. Total estimated emissions by greenhouse gas (GHG) type for 2020 and 2007.

GHG contribution	Tonnes CO_2e (2020)	Tonnes CO_2e (2007)
CH_4 (biogenic)	63,137,715	55,670,322
Aggregate CO_2e[A]	34,318,643	33,264,256
N_2O	13,890,770	12,394,592
CO_2 (LUC)	13,597,057	13,089,455
CO_2 (fossil)	8,207,251	7,439,817
CH_4 (fossil)	579,132	457,980
CO_2 (sequestration)	(1,758,248)	(1,857,771)
Total	131,972,320	120,458,652
Total without sequestration	133,730,568	122,316,423

[A] Note that aggregate CO_2e cannot be disaggregated due to data limitations, however, because these emissions are from feed inputs, GHGs are likely to be split mostly between CO_2 and N_2O categories.

Table S30. Variation in emission intensity and total emissions across dairy production regions in 2020.

Regions	Kg CO_2e/kg FPCM	Tonnes FPCM	Tonnes CO2e
Northern Plains	1.52	2.3M	3,503,430
Upper Midwest	1.25	22.2M	27,684,227
Great Lakes	1.31	9.7M	12,773,435
Intermountain	1.17	10.9M	12,772,182
Southwest	1.24	15.0M	18,573,499
Pacific Northwest	1.23	4.4M	5,428,662
West	1.32	19.2M	25,331,024
Northeast	1.39	11.6M	16,090,700
New England	1.32	1.9M	2,475,043
Mid-Atlantic	1.45	2.3M	3,365,328
Mississippi Valley	1.88	0.7M	1,241,404
Southeast	1.54	1.8M	2,733,385
**US**	1.29	101.92M	131,972,320

Section S7. Sensitivity & Uncertainty Analysis

Table S32. Estimated weighted average and range of dVS, urinary N and fecal N excreted per day across regions in this study.

Cattle Category	dVS kg/day	Fecal N g/day	Urinary N g/day	Total N g/day
Lactating Dairy Cows	6.9[a] Range: 2.9 – 7.2	158[a] Range: 119 – 167	181[a] Range: 154 – 198	338[a] Range: 283 – 366
	[6.0[a] Range: 4.8 – 6.5][b]	NA	NA	[497[a] Range: 433 – 549]
	{7.5}[c,b]	NA	NA	{416[a] Range: 355 – 451}
Dry Cows	3.4[a] Range: 3.4 – 3.5	161[a] Range: 157 – 164	97[a] Range: 93 – 101	258[a] Range: 250 – 265
	[3.8[a] Range: 3.5 – 3.9][b]	NA	NA	[217[a] Range: 210 – 228]
	{3.8}[c,b]	NA	NA	{248[a] Range: 240 – 255}
Heifer Replacements	1.8[a] Range: 1.78 – 1.80	130[a] Range: 125 – 134	55[a] Range: 49 – 61	185[a] Range: 174 – 194
	[2.4[a] Range: 1.9 – 2.6][b]	NA	NA	[176[a] Range:161 – 190]
	{4.3}[c,b]	NA	NA	{175[a] Range: 163 – 187}
Bulls	1.3[a] Range: 1.2 – 1.4	112[a] Range: 105 – 124	52[a] Range: 48 – 56	164[a] Range: 156 – 176
	[2.1[a] Range: 1.4 – 2.3][b]	NA	NA	[147[a] Range: 132 – 163]
	{5.4}[c,b]	NA	NA	{147[a] Range: 135 – 160}

[ ] indicates comparison using IPCC 2019 guidelines [12] (see Supplemental Information equation 16 and 17). This method is deployed for the EPA U.S. national GHG inventories [13].
{ } indicates comparison using the recent USDA Blue Book (2024) guidelines for quantifying GHG fluxes in agriculture and forestry (see Supplemental Information equation 18-20) [14].
[a] Represents weighted average
[b] Represents total volatile solids
[c] USDA blue book (2024) assumes 11 kg VS/1000 kg animal/day for lactating cows, 5.6 kg VS/1000 kg animal/day for dry cows, and 7.3 kg VS/1000 kg animal/day for heifers [14]

Table S33. Comparison of enteric methane and manure emissions across models, including range across regions and weighted average (in bold).

Model Comparisons	Enteric Methane	Nitrogen Excretion	Manure Methane
This study (average)	**17.4** (16.4 – 18.0) MJ/day[A]<br><br>**0.76** (0.73 – 1.22) MJ/kg FPCM[B]	**338** (288 – 366) g/day[A]	**9.2** (6.3 – 11.4) g/kg FPCM[B]
This study (upper uncertainty)	**23.7** (21.5 – 24.6) MJ/day[A]<br><br>**0.98** (0.95 – 1.54) MJ/kg FPCM[B]	**379** (323 – 409) g/day[A]	**10.6** (7.6 – 14.8) g/kg FPCM[B]
This study (lower uncertainty)	**11.9** (11.5 – 12.3) MJ/day[A]<br><br>**0.56** (0.54 – 0.93) MJ/kg FPCM[B]	**298** (252 – 323) g/day[A]	**7.7** (5.1 – 10.8) g/kg FPCM[B]
Ellis (2007) DMI model (used in Thoma et al 2013)	**22.6** (19.4 – 23.3) MJ/day[A]<br><br>**0.92** (0.89 – 1.39) MJ/kg FPCM[B]	N/A	N/A
Mills et al (2003) Mits3 model (used in Rotz et al 2021)	**27.2** (25.1 – 27.7) MJ/day[A]<br><br>**1.12** (1.09 – 1.79) MJ/kg FPCM[B]	N/A	N/A

Ellis (2007) forage model (used in Capper and Cady 2020)[A]	**8.63** (8.62 – 8.65) MJ/day[A]<br><br>**0.48** (0.45 – 0.79) MJ/kg FPCM[B]	N/A	N/A
USDA Blue Book (Leytem et al 2024)	**13.8** (11.3 – 14.3) MJ/day[A]<br><br>**0.64** (0.62 – 0.95) MJ/kg FPCM[B]	**416** (355 – 451) g/day[A]	**10.0** (7.6 – 13.8) g/kg FPCM[B]
IPCC 2006/2019 (used in EPA 2023)	*Energy Balance Method*<br>**24.4** (21.5 – 25.7) MJ/day[A]<br><br>**0.95** (0.92 – 1.46) MJ/kg FPCM[B]<br><br>*Simplified Method*<br>**25.4** (22.2 – 26.1) MJ/day[A]<br><br>**1.00** (0.97 – 1.52) MJ/kg FPCM[B]	497 (433 – 549) g/day[A]	**8.3** (6.1 – 11.4) g/kg FPCM[B]
ASAE (2005) model (used in Thoma et al 2013)	N/A	**570** (522 – 595) g/day[A]	N/A
Thoma et al 2013	1.02 – 1.17 MJ/kg FPCM[C]	430 g/day [D]	7.5 - 27 g/kg FPCM[C]

[A] Based on lactating dairy cow diets specified in this study
[B] Based on diets specified in this study across all cattle categories
[C] Based on diets within referenced study across all cattle categories
[D] Based on lactating cow diets within referenced study

Figure S2. Comparison of manure and enteric methane emissions and nitrogen excretion across models [A].

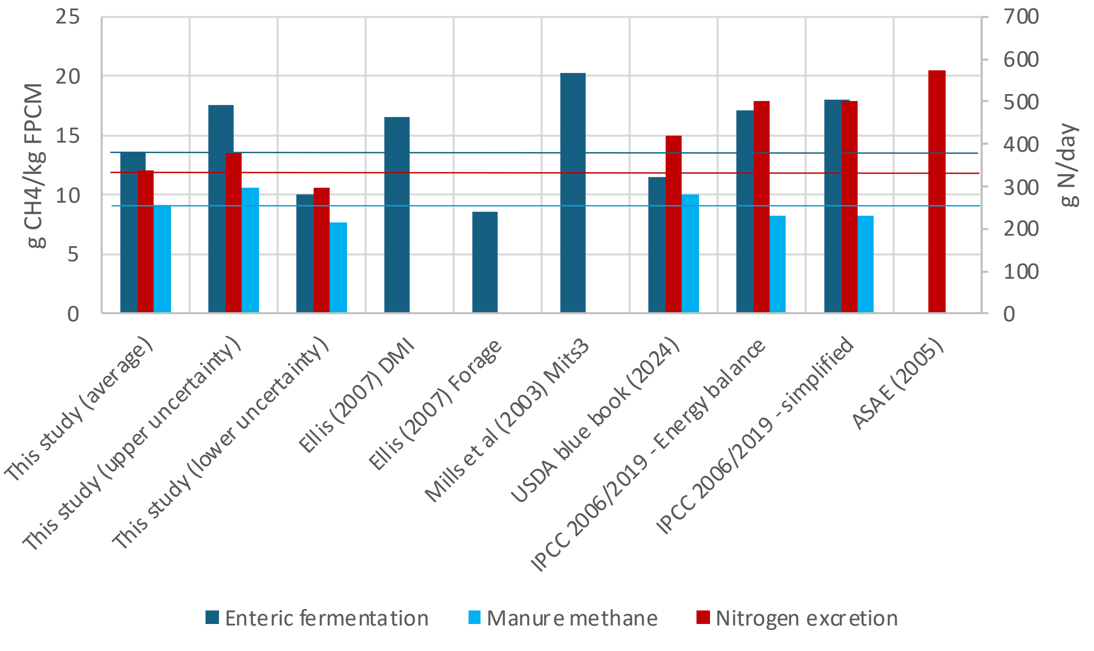

[A] Longitudinal lines are based on this study's average for ease of comparison to the alternative model estimates.
Note that the 'upper uncertainty' and 'lower uncertainty' here refers to this study's upper and lower bound standard errors on parameters for estimating manure volatile solids and nitrogen excretion.

Our study estimates enteric methane emissions for lactating dairy cows to range between 16 to 18 MJ CH_4/day across regions, with an uncertainty range spanning from 12 to 25 MJ CH_4/day. These values are relatively comparable to the estimates from the Ellis (2007) dry matter intake parameter model used by Thoma et al. (2013) [15, 16], which range from 19 to 23 MJ CH_4/day. In contrast, the Mills et al. (2003) Mits3 model, applied in the IFSM model in Rotz et al. (2021) [17], and the IPCC 2006/2019 models used in the U.S. EPA national GHG inventories [12, 13], produce higher estimates of 25 to 28 MJ CH_4/day and 22 to 26 MJ CH_4/day, respectively. On the lower end, the Ellis et al (2007) forage model, used in the Capper and Cady (2019) study [1], estimates significantly lower emissions at approximately 8.6 MJ CH_4/day. Similarly, the USDA 'Blue Book' (2024) model [14], which references the Niu et al (2018) study [18] but with a higher root mean square error (RMSE) than the model used in this study, yields a range of 11-14 MJ CH_4/day. When examining total enteric methane over the entire lifecycle, our study estimates an average of 0.76 MJ/kg FPCM across different regions, 25-35% lower compared to the estimates from Thoma et al 2013, largely due to differences in dietary assumptions, growth parameters and the enteric methane models used [16]. Our approach leverages the latest and most reliable enteric methane models, minimizing RMSE as demonstrated by Niu et al (2018) [18], and incorporates a wider variety of dietary inputs across 12 distinct geographic regions, ostensibly resulting in a more refined estimate of enteric methane emissions. Our results suggest that while enteric fermentation remains a significant source of emissions, it may be less of a contributor than previously estimated in studies using less contemporary and less detailed regional analyses.

For manure methane, previous dairy LCAs and the EPA's National GHG Inventory have used total volatile solids (VS) outputs to estimate manure emissions. This approach includes all organic matter, such as lignin, which is resistant to anaerobic digestion and does not contribute to methane production, potentially leading to an overestimation of emissions. Indeed, our study estimates a range of 6.3 to 11.4 g CH_4/kg FPCM, with an uncertainty range spanning between 5.1 to 14.8 g CH_4/kg FPCM across regions, which aligns well with the IPCC (2019) model [12] used by the EPA National GHG Inventories [13], ranging from 6.1 to 11.4 g CH_4/kg FPCM. In comparison, the USDA Blue Book (2024) model [14] yield higher estimates, with 7.6 to 13.8 g CH_4/kg FPCM, respectively, due to the greater amount of volatile solids assumed (see Table 32). Notably, Thoma et al (2013) reported an even higher range of 7.5 to 27 g CH_4/kg FPCM across five regions [16].

For manure N_2O, our study separates urinary and fecal N excretion, allowing for a more precise calculation of total nitrogen compared to traditional models that rely solely on total nitrogen (TN). This method accounts for the different ways cattle process and excrete nitrogen, resulting in more accurate overall estimates [19]. Additionally, separating urinary N from fecal N allows for more precise estimates of indirect N_2O emissions from volatilization, as only urinary N is subject to this process. In our study, nitrogen excretion for lactating dairy cows ranges from 288 to 366 g/day across regions, with an uncertainty range of 252 to 409 g/day. This aligns reasonably well with estimates from the USDA Blue Book (2024) models [14], which range from 355 to 451 g/day. By contrast, the ASAE (2005) model [20] used by Thoma et al. (2013) [16] and the IPCC models [12] estimate nitrogen excretion to be higher, between 522 to 595 g/day and 433 to 549 g/day, respectively. Overall, our study's lower estimates for CH_4 and N_2O emissions from manure compared to previous studies highlight that while manure management is an important source of emissions, its contribution may be less significant than suggested by earlier assessments.

Figure S3. Uncertainty analysis considering uncertainty bounds in feed emission factors, dry matter intake, enteric fermentation models, dVS models and nitrogen excretion models, and implication for total estimated A) CO_2e emission intensity across regions, B) total MT CO_2e emissions across regions, and C) total MT CO_2e emissions across the US.

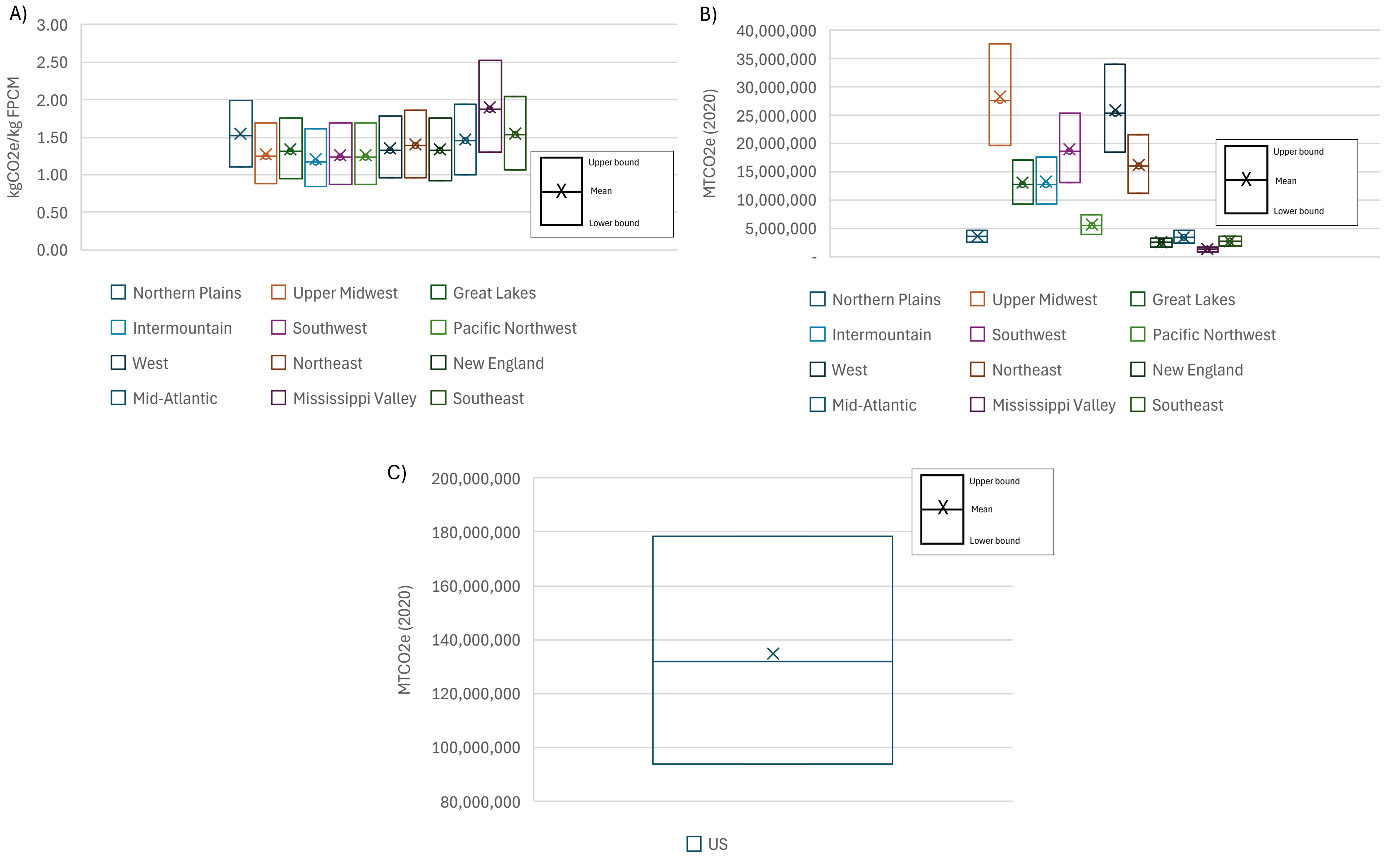

Figure S4. Contribution of emission sources across regions, considering A) upper bound standard deviation, B) average parameter values, C) lower bound standard deviation on feed emission factors, dry matter intake, enteric methane formation, volatile solids and nitrogen excretion.

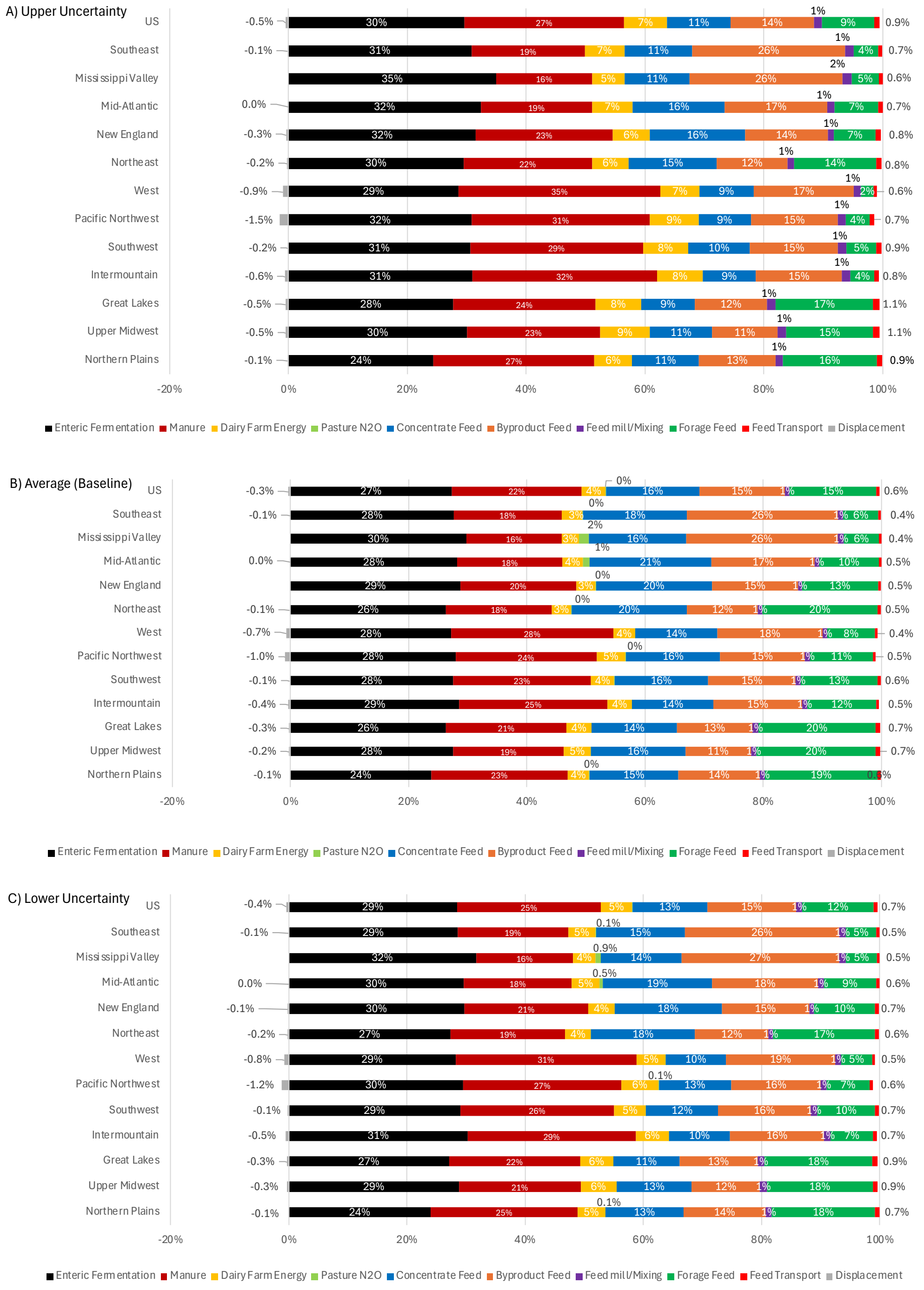

Table S34. Total annual MT CO_2e emissions (2020) with upper and lower bound uncertainty considerations.

Uncertainty Parameters	Feed Emission Factors		Feed Dry Matter Intake		Enteric fermentation Methane formation		Manure (VS and N extraction outputs)		All Scenario uncertainty	
Regions	Upper Uncertainty	Lower Uncertainty	Upper Uncertainty	Lower Uncertainty	Upper Uncertainty	Lower Uncertainty	Upper uncertainty	Lower Uncertainty	Upper uncertainty	Lower Uncertainty
Northern Plains	3,934,586	3,068,257	3,840,311	3,166,585	3,659,569	3,347,292	3,591,517	3,416,269	4,574,259	2,547,392
Upper Midwest	32,167,041	23,841,634	30,276,971	25,091,773	29,170,240	26,198,215	28,424,284	26,953,621	37,578,610	19,563,025
Great Lakes	14,628,898	11,155,195	13,979,370	11,567,628	13,423,968	12,122,902	13,109,010	12,441,846	17,070,491	9,193,827
Intermountain	14,973,003	11,303,042	13,984,011	11,560,500	13,505,772	12,038,593	13,147,161	12,401,327	17,586,960	9,207,959
Southwest	21,644,551	15,955,825	20,326,663	16,820,533	19,578,472	17,568,525	19,123,140	18,028,846	25,359,267	13,015,807
Pacific Northwest	6,332,307	4,700,043	5,946,686	4,910,693	5,729,598	5,127,725	5,593,257	5,266,347	7,435,433	3,821,574
West	29,052,942	22,426,590	27,762,932	22,899,375	26,676,412	23,985,636	26,027,351	24,641,664	34,035,065	18,388,190
Northeast	18,533,975	13,483,414	17,622,390	14,559,162	16,893,335	15,288,065	16,488,252	15,697,500	21,587,176	11,094,979
New England	2,809,792	2,118,239	2,710,258	2,239,854	2,605,773	2,344,314	2,541,427	2,409,569	3,288,149	1,735,270
Mid-Atlantic	3,843,672	2,850,654	3,683,069	3,047,622	3,535,885	3,194,772	3,480,767	3,250,826	4,508,587	2,314,241
Mississippi Valley	1,424,511	1,059,296	1,359,114	1,123,708	1,303,455	1,179,353	1,284,349	1,198,841	1,669,539	860,108
Southeast	3,087,276	2,337,014	2,993,624	2,473,175	2,869,586	2,597,185	2,825,227	2,642,268	3,623,868	1,902,328
US	152,432,552	114,299,203	144,485,400	119,460,607	138,952,063	124,992,577	135,635,742	128,348,923	178,317,403	93,644,701

Table S35. Emission intensity (2020) kg CO_2e/kg FPCM with upper and lower bound uncertainty considerations.

Uncertainty Parameters	Feed Emission Factors		Feed Dry Matter Intake		Enteric fermentation Methane formation		Manure (VS and N extraction outputs)		All Scenario uncertainty	
Regions	Upper Uncertainty	Lower Uncertainty	Upper Uncertainty	Lower Uncertainty	Upper Uncertainty	Lower Uncertainty	Upper uncertainty	Lower Uncertainty	Upper uncertainty	Lower Uncertainty
Northern Plains	1.71	1.34	1.67	1.38	1.59	1.46	1.56	1.49	1.99	1.11
Upper Midwest	1.45	1.07	1.37	1.13	1.32	1.18	1.28	1.22	1.69	0.88
Great Lakes	1.50	1.14	1.43	1.19	1.38	1.24	1.35	1.28	1.75	0.94
Intermountain	1.37	1.04	1.28	1.06	1.24	1.10	1.20	1.14	1.61	0.84
Southwest	1.44	1.06	1.36	1.12	1.31	1.17	1.28	1.20	1.69	0.87
Pacific Northwest	1.44	1.07	1.35	1.12	1.30	1.16	1.27	1.20	1.69	0.87
West	1.52	1.17	1.45	1.20	1.39	1.25	1.36	1.29	1.78	0.96
Northeast	1.60	1.16	1.52	1.26	1.46	1.32	1.42	1.35	1.86	0.96
New England	1.50	1.13	1.45	1.20	1.39	1.25	1.36	1.29	1.76	0.93
Mid-Atlantic	1.65	1.23	1.59	1.31	1.52	1.38	1.50	1.40	1.94	1.00
Mississippi Valley	2.15	1.60	2.05	1.70	1.97	1.78	1.94	1.81	2.52	1.30
Southeast	1.74	1.32	1.68	1.39	1.61	1.46	1.59	1.49	2.04	1.07
US	1.50	1.12	1.42	1.17	1.36	1.23	1.33	1.26	1.75	0.92

Table S36. Comparison of the total cradle-to-farmgate million metric tonnes (MMT) of CO_2e emissions and emissions intensity (kgCO_2e/kg FPCM) without displacement credits from biogas utilization, land use change (LUC), and with silage storage N_2O. Values in [ ] represents the percent change in emissions through exclusion of emission source.

Regions	Total		Without Displacement		Without LUC		With silage storage N2O	
	MMT	EF	MMT	EF	MMT	EF	MMT	EF
Northern Plains	3.50	1.52	3.51 [0.1%]	1.53	3.20 [-8.7%]	1.39	3.56 [1.7%]	1.55
Upper Midwest	27.68	1.25	27.78 [0.3%]	1.25	24.84 [-10.3%]	1.12	28.35 [2.4%]	1.28
Great Lakes	12.77	1.31	12.82 [0.3%]	1.32	11.67 [-8.6%]	1.20	13.02 [1.9%]	1.34
Intermountain	12.77	1.17	12.84 [0.5%]	1.18	11.64 [-8.9%]	1.07	13.12 [2.7%]	1.20
Southwest	18.57	1.24	18.60 [0.1%]	1.24	16.74 [-9.9%]	1.12	19.01 [2.4%]	1.27
Pacific Northwest	5.43	1.23	5.49 [1.2%]	1.25	4.88 [-10.2%]	1.11	5.55 [2.2%]	1.26
West	25.33	1.32	25.54 [0.8%]	1.33	23.34 [-7.9%]	1.22	25.74 [1.6%]	1.34
Northeast	16.09	1.39	16.12 [0.2%]	1.39	14.04 [-12.7%]	1.21	16.45 [2.2%]	1.42
New England	2.48	1.32	2.48 [0.1%]	1.33	2.14 [-13.7%]	1.14	2.51 [1.6%]	1.34
Mid-Atlantic	3.37	1.45	3.37 [0.0%]	1.45	2.93 [-12.8%]	1.26	3.40 [0.9%]	1.46
Mississippi Valley	1.24	1.88	1.24 [0.0%]	1.88	1.15 [-7.3%]	1.74	1.25 [0.5%]	1.88
Southeast	2.73	1.54	2.74 [0.1%]	1.54	2.47 [-9.8%]	1.39	2.75 [0.4%]	1.54
US	131.97	1.29	132.50 [0.4%]	1.30	118.95 [-9.9%]	1.17	134.71 [2.1%]	1.32

Table S37. Sensitivity analysis of total 2020 dairy cradle-to-farmgate emissions and contribution by GHG type across IPCC Assessment Reports (AR) GWP100 factors.

GHGs (MTCO_2e)	AR6[A]	AR5 with Climate Carbon Feedback [B]	AR5 no Climate Carbon Feedback [C]	AR4[D]
Aggregate CO_2e[E]	34.3M<br>26%	34.3M<br>23%	34.3M<br>26%	34.3M<br>27%
N_2O	13.9M<br>11%	14.6M<br>10%	13.7M<br>10%	14.6M<br>11%
CH_4 (biogenic)	63.1M<br>48%	78.9M<br>53%	65.0M<br>49%	58.0M<br>46%
CH_4 (fossil)	0.6M<br>0.4%	0.7M<br>0.5%	0.6M<br>0.4%	0.5M<br>0.4%
CO2 (LUC)	13.6M<br>10%	13.6M<br>9%	13.6M<br>10%	13.6M<br>11%
CO2 (fossil)	8.2M<br>6%	8.2M<br>6%	8.2M<br>6%	8.2M<br>6%
CO2 (sequestration)	-1.8M<br>-1%	-1.8M<br>-1%	-1.8M<br>-1%	-1.8M<br>-1%
Total	132.0M	148.6M	133.8M	127.5M
kgCO_2e/kg FPCM	1.29	1.46	1.31	1.25

[A] CH_4 (biogenic) GWP = 27, CH_4 (fossil) GWP = 29.8, N_2O GWP = 273
[B] CH_4 (biogenic) GWP = 34, CH_4 (fossil) GWP = 36, N_2O GWP = 298
[C] CH_4 (biogenic) GWP = 28, CH_4 (fossil) GWP = 30, N_2O GWP = 265
[D] CH_4 (biogenic and fossil) = 25, N_2O GWP = 298
[E] From feed emissions factors that are unable to be disaggregated by GHG type due to data source limitations.

Table S38. Combined uncertainty in emission intensity and annual total emission estimates in 2007 and 2020.

Uncertainty	Emission intensity (kgCO_2e/kg FPCM)		Annual emissions (million MT CO_2e)	
	2007	2020	2007	2020
Upper uncertainty	2.09	1.75	168	178
Average	1.50	1.29	121	132
Lower uncertainty	1.01	0.92	81	94

References

[1] J. Capper and R. Cady, "The effects of improved performance in the U.S. dairy cattle industry on environmental impacts between 2007 and 2017," *Journal of Animal Science,* vol. 98, no. 1, pp. 1-13, 2020.

[2] USDA NASS, "Cattle," United States Department of Agriculture National Agricultural Statistics Service (NASS), 2024.

[3] USDA NASS, "Livestock Slaughter 2020 Summary," United States Department of Agriculture National Agricultural Statistics Service, 2021.

[4] R. Pelton, T. Lark, S. Spawn, N. Springer and J. Schmitt, "Land use leverage points to reduce GHG emissions in U.S. agricultural supply chains," *Environmental Research Letters,* vol. 16, p. 115002, 2021.

[5] R. Pelton, C. Kazanski, S. Keerthi, K. Racette, S. Gennet, N. Springer, E. Yacobson, M. Wironen, D. Ray, K. Johnson and J. Schmitt, "Greenhouse gas emissions in US beef production can be reduced by up to 30% with the adoption of selected mitigation measures," *Nature Food,* vol. 5, pp. 787-797, 2024.

[6] Argonne National Laboratory, "R&D GREET Model," 2024. [Online]. Available: https://greet.anl.gov. [Accessed 3 January 2025].

[7] GFLI, "Global Metrics for Sustainable Feed," 2024. [Online]. Available: https://globalfeedlca.org. [Accessed 5 October 2024].

[8] Sphera, "LCA for Experts Software," 2024. [Online]. Available: https://about.sphera.com/. [Accessed 3 January 2025].

[9] R. Higgs, L. Chase, D. Ross and M. Van Amburgh, "Updating the Cornell Net Carbohydrate and Protein System feed library and analyzing model sensitivity to feed inputs," *Journal of Dairy Science,* vol. 98, no. 9, pp. 6340-6360, 2015.

[10] M. Van Amburgh and E. e. a. Collao-Saenz, "The Cornell Net Carbohydrate and Protein System: Updates to the model and evaluation of version 6.5," *Journal of Dairy Science,* vol. 98, no. 9, pp. 6361-6380, 2015.

[11] M. Galyean, N. Cole, L. Tedeschi and M. Branine, "Efficiency of converting digestible energy to metabolizable energy and reevaluation of the California Net Energy System maintenance requirements and equations for predicting dietary net energy values for beef cattle1," *Journal of Animal Science,* vol. 94, pp. 1329-0223, 2016.

[12] O. Gavrilova, A. Leip, H. Dong, J. MacDonald, C. Bravo, B. Amon, R. Rosale, A. Prado, M. Lima, W. Oyhantcabal, T. van der Weerden and Y. Widiawati, "Chapter 10: Emissions from Livestock and Manure Management," in *2019 Refinement to the 2006 IPCC Guidelines for National Greenhouse Gas Inventories - Volume 4: Agriculture, Forestry, and Other Land Use*, 2019, pp. 10.9-10.180.

[13] US EPA, "Inventory of U.S. Greenhouse Gas Emissions and Sinks: 1990-2020: Annex 3," United States Environmental Protection Agency, 2022.

[14] A. Leytem, S. Archibeque, N. Cole, S. Gunter, A. Hristov, K. Johnson, E. Kebreab, R. Kohn, W. Liao, C. Toureene and J. Tricarico, "Chapter 4: Quantifying greenhouse gas sources and sinks in animal production systems," in *Quantifying greenhouse gas fluxes in agriculture and forestry: Methods for entity-scale inventory*, 2024, pp. 4.1 - 4.64.

[15] J. Ellis, E. Kebreab, N. Odongo, B. McBride, E. Okine and J. France, "Prediction of Methane Production from Dairy Beef Cattle," *Journal of Dairy Science,* vol. 90, pp. 3456-3467, 2007.

[16] G. Thoma, J. Popp, D. Shonnard, D. Nutter, M. Matlock, R. Ulrich, W. Kellogg, D. Kim, Z. Neiderman, N. Kemper, F. Adom and C. East, "Regional analysis of greenhouse gas emissions from USA dairy farms: A cradle to farm-gate assessment of the American dairy industry circa 2008," *International Dairy Journal,* vol. 31, pp. S29-S40, 2013.

[17] A. Rotz, R. L. A. Stout, G. Feyereisen, H. Waldrip, G. Thoma, M. Holly, D. Bjorneberg, J. Baker, P. Vadas and Kleinman, "Environmental assessment of United States dairy farms," *Journal of Cleaner Production,* vol. 315, no. 128153, pp. 1-13, 2021.

[18] M. Niu, E. Kebreab, A. Hristov, J. Oh, C. Arndt, A. Bannink, A. Bayat, A. Brito, T. Boland, D. Casper, L. Crompton, J. Dijkstra, M. Eugene, P. Garnswothry, M. Haque, A. Hellwing, P. Huhtanen, M. Kreuzer, B. Kuhla, P. Lund and Z. Yu, "Prediction of enteric methane production, yield and intensity in dairy cattle using an intercontinental database," *Global Change Biology,* pp. 3368-3389, 2018.

[19] K. Reed, L. Moraes, D. Casper and E. Kebreab, "Predicting nitrogen excretion from cattle," *Journal of Dairy Science,* vol. 98, pp. 3025-3035, 2015.

[20] ASAE, "Manure Production and Characteristics," American Society of Agricultural Engineers, 2005.

[21] A. Asselin-Balencon, J. Popp, A. Henderson, M. Heller, G. Thoma and O. Jolliet, "Dairy farm greenhouse gas impacts: A parsimonius model for a farmer's decision support tool," *International Dairy Journal,* vol. 31, pp. S65-S77, 2013.

[22] M. de Ondarza and J. Tricarico, "Nutritional contributions and non-CO2 greenhouse as emissions from human-inedible byproduct feeds consumed by dairy cows in the United States," *Journal of Cleaner Production,* vol. 315, no. 128125, p. 107358, 2021.

[23] W. Gaines and F. Davidson, "Relation between percentage fat content and yield of milk," *Bulletin 245, University of Illinois Agriculture Experimental Station,* 1923.

[24] NASEM, "Nutrient Requirements of Dairy Cattle: 8th revised edition," National Academies of Sciences, Engineering, and Medicine, 2021.

[25] NRC, "Nutrient Requirements of Dairy Cattle," National Academy Press, Washington, DC, 2001.

[26] USDA, "Federal Milk Order Market Statistics 2020 Annual Summary," *Statistical Bulletin,* vol. 1021, 2022.

[27] USDA NASS, "Milk Disposition and Income Final Estimates 2003-2007," *Statistical Bulletin ,* vol. 1027, pp. 1-26, 2009.

[28] USDA NASS, "Milk Production, Disposition, and Income 2022 Summary," 2023.

[29] USDA, "Agricultural Census: Inventory of Milk Cows," 2017.

[30] International Dairy Federation (IDF), "The IDF global Carbon Footprint standard for the dairy sector," *Bulletin of the International Dairy Federation,* vol. 520, pp. 1-106, 2022.

[31] USDA, "Agricultural Census Fertilizre Use and Price," 2019.

[32] L. Moraes, A. Strathe, D. Fadel, D. Casper and E. Kebreab, "Prediction of enteric methane emissions from cattle," *Global Change Biology,* vol. 20, pp. 2140-2148, 2014.

[33] US EPA, "Title 40, Chapter 1, Subchapter C, Part 98, Subpart JJ- Manure Management," 2024.

[34] K. Hales, C. Coppin, Z. Smith, Z. McDaniel, L. Tedeschi, N. Cole and M. Galyean, "Predicting metabolizable energy from digestible energy for growing and finishing beef cattle and relationships to the prediction of methane," *Journal of Animal Science,* vol. 100, no. 3, pp. 1-11, 2022.

[35] W. Weiss and A. Tebbe, "Estimating digestible energy values of feeds and diets and integrating those values into net energy systems," *Translational Animal Science,* vol. 3, no. 3, pp. 953-961, 2019.

[36] J. Greene, J. Wallace, R. Williams, A. Leytem, B. Bock, M. McCully, S. Kaffka, A. Rotz and J. Quinn, "National Greenhouse Gas Emissions Reduction Potential from Adopting Anaerobic Digestion on Large-Scale Dairy Farms in the United States," *Environmental Science & Technology,* vol. 58, pp. 12409-12419, 2024.

[37] FARM (Farmers Assuring Responsible Management), 2022. [Online]. Available: https://nationaldairyfarm.com/. [Accessed 3 January 2025].

[38] USDA, "Producer Milk Components Report," 2021. [Online]. Available: https://mymarketnews.ams.usda.gov/filerepo/sites/default/files/3462/2021-12-30/551255/ams_3462_00014.pdf. [Accessed 3 January 2025].

[39] USDA ERS, "Dairy Data," 2024. [Online]. Available: https://www.ers.usda.gov/data-products/dairy-data/dairy-data. [Accessed 3 January 2025].

[40] INRA-CIRAD-AFZ, "Feed Tables," 2024. [Online]. Available: https://www.feedtables.com. [Accessed 3 January 2025].

[41] Food and Agriculture Organization, "Environmental performance of large ruminant supply chains: Guidelines for Assessment," 2016. [Online]. Available: https://openknowledge.fao.org/items/143aa579-884c-4c0e-8bbd-3a6cc7866636. [Accessed 3 January 2025].

[42] Agricultural Marketing Resource Center, "Productionand Revenue Trends in Corn Ethanol, DDGS, and Corn Distillers Oil," 2018. [Online]. Available: https://www.agmrc.org/renewable-energy/renewable-energy-climate-change-report/renewable-energy-climate-change-report/march-2018-report/production-and-revenue-trends-in-corn-ethanol-ddgs-and-corn-distillers-oil. [Accessed 3 January 2025].

[43] K. Hergoualc'h, H. Akiyama, M. Bernoux, N. Chirinda, A. Prado, A. Kasimir, J. MacDonald, S. Ogle, K. Regina and T. van der Weerden, "Chapter 11: N2O emissions from managed soils, and CO2 emissions from lime and urea application," in *2019 Refinement to the 2006 IPCC Guidelines for National Greenhouse Gas Inventories: Volume 4 - Agriculture, Forestry, and Other Land Use*, 2019, pp. 11.1 - 11.41.
